# A *cis*-regulatory element promoting increased transcription at low temperature in cultured ectothermic *Drosophila* cells

**DOI:** 10.1186/s12864-021-08057-4

**Published:** 2021-10-28

**Authors:** Yu Bai, Emmanuel Caussinus, Stefano Leo, Fritz Bosshardt, Faina Myachina, Gregor Rot, Mark D. Robinson, Christian F. Lehner

**Affiliations:** 1 Department of Molecular Life Sciences, University of Zurich, Winterthurerstrasse 190, 8057 Zurich, Switzerland; 2 SIB Swiss Institute of Bioinformatics, University of Zurich, Winterthurerstrasse 190, 8057 Zurich, Switzerland

**Keywords:** Cool temperature acclimation, Ectotherm, *Drosophila*, S2R+ cells, Transcriptional control, *cis*-regulatory element (CRE), *His2Av*, *Ets97D*, NRF-2/GABP, *pastrel (pst)*

## Abstract

**Background:**

Temperature change affects the myriad of concurrent cellular processes in a non-uniform, disruptive manner. While endothermic organisms minimize the challenge of ambient temperature variation by keeping the core body temperature constant, cells of many ectothermic species maintain homeostatic function within a considerable temperature range. The cellular mechanisms enabling temperature acclimation in ectotherms are still poorly understood. At the transcriptional level, the heat shock response has been analyzed extensively. The opposite, the response to sub-optimal temperature, has received lesser attention in particular in animal species. The tissue specificity of transcriptional responses to cool temperature has not been addressed and it is not clear whether a prominent general response occurs. *Cis*-regulatory elements (CREs), which mediate increased transcription at cool temperature, and responsible transcription factors are largely unknown.

**Results:**

The ectotherm *Drosophila melanogaster* with a presumed temperature optimum around 25 °C was used for transcriptomic analyses of effects of temperatures at the lower end of the readily tolerated range (14–29 °C). Comparative analyses with adult flies and cell culture lines indicated a striking degree of cell-type specificity in the transcriptional response to cool. To identify potential *cis*-regulatory elements (CREs) for transcriptional upregulation at cool temperature, we analyzed temperature effects on DNA accessibility in chromatin of S2R+ cells. Candidate *cis*-regulatory elements (CREs) were evaluated with a novel reporter assay for accurate assessment of their temperature-dependency. Robust transcriptional upregulation at low temperature could be demonstrated for a fragment from the *pastrel* gene, which expresses more transcript and protein at reduced temperatures. This CRE is controlled by the JAK/STAT signaling pathway and antagonizing activities of the transcription factors Pointed and Ets97D.

**Conclusion:**

Beyond a rich data resource for future analyses of transcriptional control within the readily tolerated range of an ectothermic animal, a novel reporter assay permitting quantitative characterization of CRE temperature dependence was developed. Our identification and functional dissection of the *pst*_E1 enhancer demonstrate the utility of resources and assay. The functional characterization of this CoolUp enhancer provides initial mechanistic insights into transcriptional upregulation induced by a shift to temperatures at the lower end of the readily tolerated range.

**Supplementary Information:**

The online version contains supplementary material available at 10.1186/s12864-021-08057-4.

## Background

Many habitats on earth experience temperature fluctuations of variable scale and temporal dynamics. Life in these thermally unstable habitats is a major challenge because temperature change affects all biological processes, but importantly not in a uniform manner. In general, diffusion is less temperature-dependent than enzymatic reactions, and the latter have individual temperature profiles. Thus, temperature acclimation is predicted to require complex regulation of cellular processes. Endothermic organisms, like humans, largely circumvent this challenge by keeping the core body temperature constant at the expense of metabolic energy. However, the large majority of organisms (microorganisms, plants and most animals including the model organism *Drosophila melanogaster*) are ectotherms. Their cells function over an often surprisingly wide range of ambient temperature. The cellular mechanisms enabling temperature acclimation are still poorly understood.

At the transcriptional level, considerable progress has been made in case of the heat shock response (HSR), which was discovered early on. Polytene chromosomes from salivary glands of *Drosophila busckii* larvae were observed to display a distinct chromosome puffing pattern in response to elevated temperature [1]. This evidence for transcriptional induction eventually led to the molecular isolation and characterization of the highly conserved *Hsp* genes [2, 3]. Their products, the heat shock proteins (HSPs), function primarily as chaperones, which prevent or reverse protein misfolding and provide an environment for proper protein folding. The transcriptional activation of *Hsp* genes in response to heat shocks has served as an important experimental paradigm for research on the molecular mechanisms of transcriptional control. Heat shocks activate the transcription factor HSF1 and thereby cause a release of paused RNA polymerase II from sites downstream of the *Hsp* promoters into productive elongation. The HSR can be induced by stressors other than heat shock (like oxidative or heavy metal stress, glucose depletion). *Hsp* genes are also induced during recovery from severe cold shock [4–8]. However, HSF1 is not responsible for transcriptional induction of all of the many heat-shock induced genes in mammals and yeast [9, 10]. More than half of the heat shock-induced genes are activated in a HSF1-independent manner in mammalian cells [9]. In addition, heat shock represses more genes than it induces, and the heat-induced repression is entirely HSF1-independent [9].

In comparison to acclimation to elevated temperature, the opposite, i.e., the cellular response to temperature decrease has received less attention, in particular in animal organisms. For the relatively immotile microbial and plant organisms, cold is less avoidable and transcriptional responses have been characterized more extensively [11–14]. In plants, considerable insight concerning crucial transcription factors (TFs) and their regulation by upstream thermosensors for responses to both extreme cold and more modest cool temperatures has been obtained. The ICE-CBF TFs regulate cold response (COR) genes important for cold tolerance in many plant species [14]. In case of vernalization, the process by which wintertime chill stimulates springtime flowering, epigenetic repression of the *FLC* locus by Polycomb factors is central in *Arabidopsis thaliana* [15, 16]. In thermomorphogenesis, i.e., the morphological changes according to ambient temperature, transcription factors (TFs) of the PIF family, which are controlled by both light and temperature, are crucial [17, 18]. Beyond PIFs, HSF family proteins also induce a large part of the warm transcriptome via eviction of + 1 nucleosomes containing the histone H2A variant H2A.Z in temperature responsive genes [19, 20].

To characterize transcriptional responses to low temperature within the readily tolerated range in an ectothermic animal, we have chosen *Drosophila melanogaster*. For this fly, the temperature range for successful completion of the entire life cycle is commonly reported to be 14 to 29 °C, and 25 °C is considered to be optimal [21]. *D. melanogaster* and closely related species have already been used extensively for research on effects and responses to low temperature [22, 23]. Considerable progress concerns interactions of ambient temperature with the circadian system, which involves transcriptional and post-transcriptional control [24, 25]. In regard to transcriptional control, a majority of the published literature concerns exposure to severe cold, but a few studies have also reported transcriptome analyses within the readily tolerated range of 14–29 °C [26–30]. These studies with adult females, ovaries and adult fly heads have detected extensive transcriptome changes.

Analysis of whole animals and organs likely augments the complexity of transcriptomic analyses, as distinct tissue - and cell types might express specific or even opposite responses to low temperature in case of particular pathways. Indeed, our comparisons with adult male flies and two cultured cell lines described here reveal a surprising degree of cell-type specificity in the transcriptional response to temperatures at the lower end of the readily tolerated range. Therefore, to reduce response complexity, analyses were focused on the *D. melanogaster* S2R+ cell line [31]. Temperature-dependence of the transcriptome was analyzed using DNA microarrays and 3′ RNA-Seq, and compared with that in adult male flies and another cell line. The temporal dynamics of the S2R+ cell transcriptome after a shift to 14 °C was also analyzed. Combined with our data on temperature-dependence of DNA accessibility in chromatin acquired with ATAC-Seq [32], we identified candidate *cis*-regulatory elements (CREs) driving transcriptional upregulation at low temperature. These CREs were further analyzed with a novel reporter assay for accurate evaluation of their temperature-dependency. Robust transcriptional upregulation at low temperature could be demonstrated in particular for a fragment from the *pastrel* (*pst*) gene, and its activity was found to be controlled by the JAK/STAT signaling pathway and the antagonizing activities of the transcription factors Pointed and Ets97D.

## Results

### The temperature range from 14 to 29 °C is readily tolerated by *Drosophila* S2R+ cells

**Fig. 1:**
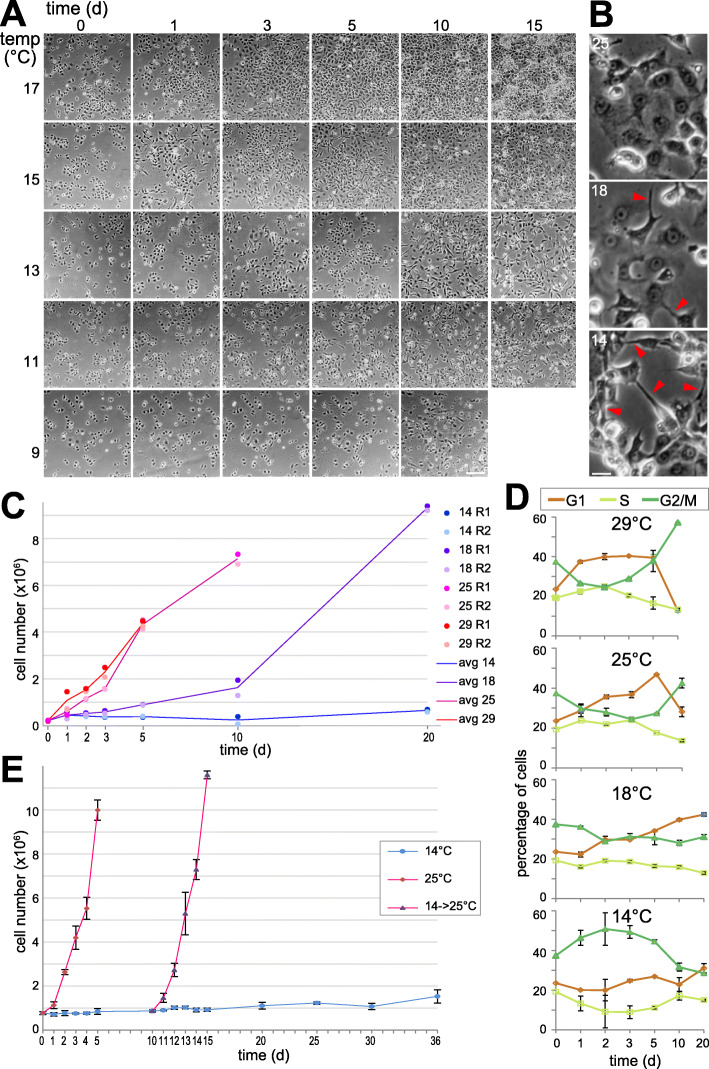
Proliferation of S2R+ cells at suboptimal temperatures. (**A**, **B**) For microscopic evaluation of cell proliferation, cells were plated in aliquots and shifted to the indicated suboptimal temperatures. Phase contrast images of the same regions were acquired at the indicated times after downshift. (**B**) High magnification views of cells grown to comparable cell density at the indicated temperatures illustrate increased cell aggregation and longer extensions (arrowheads) at low temperature. Scale bar = 50 μm (**A**) and 10 μm (**B**). (**C**) Cells were counted to assess cell proliferation at different temperatures (14, 18, 25 and 29 °C). Culture aliquots were shifted to the different target temperatures, followed by counting at the indicated times after the shift. Two replicates (R1 and R2) were analyzed for each temperature and time point. Counts of viable cells in the two replicates and their mean are displayed. (**D**) Temperature effects on the cell cycle profile. Additional culture aliquots beyond those used for the counting shown in panel (**C**) were analyzed by flow cytometry after DNA staining for identification of cells in the G1, S and G2/M phase. Mean and standard deviation (s.d.) are displayed (*n* = 2). (**E**) Immediate recovery of cell proliferation rate at 25 °C after prolonged incubation at 14 °C. Culture aliquots were shifted to either 14 or 25 °C. Moreover, after 10 days of incubation at 14 °C, some aliquots were transferred back to 25 °C. Mean and s.d. of the counts of viable cells are displayed (*n* = 3)

*Drosophila* cell lines, including S2R+ cells, are usually cultured around the presumed optimal temperature of 25 °C. To assess the range of suboptimal temperatures permissive for proliferation of S2R+ cells (Fig. 1A), replicate cultures were plated, followed by incubation at different temperatures (17, 15, 13, 11, and 9 °C). To monitor cell proliferation after the temperature shift, phase contrast micrographs of the same culture regions were taken at intervals (day 0, 1, 3, 5, 10 and 15). At 9, 11 and 13 °C, a marginal increase in cell numbers was apparent. However, in parallel a clear increase in cell debris was observed in particular at 9 °C, indicating substantial cell death (Fig. 1A). At 13 °C, cells were more spindle shaped at the late time points and a noticeable fraction of cells had vacuoles in the end (day 15). At 15 °C, cell proliferation was evident with only mild effects on cell morphology. Even stronger proliferation was observed at 17 °C, without obvious effects on cell morphology.

For further characterization, we analyzed replicate cultures after shifts to different temperatures (29, 25, 18 and 14 °C) by counting the number of live and dead cells apart from phase contrast microscopy. Moreover, flow cytometry was used for monitoring cell cycle profiles. Again, S2R+ cells were more spindle shaped overall with a greater cell aggregation tendency at the lowest temperature (14 °C) (Fig. 1B). The number of live cells did not increase steadily at this lowest temperature (Fig. 1C). After an initial doubling within the first day, live cell numbers slowly decreased back to initial values, followed by another minor increase. This eventual increase of live cells was observed in three independent experiments (fold change between day 5 and 20 = 3.2, 1.9 and 1.4). Flow cytometric analysis of cell cycle profiles over time indicated that S2R+ cells arrest in the G2 phase when entering the stationary phase after growth at 29 or 25 °C (Fig. 1D). At 14 °C, however, G2 cell enrichment was observed early after the temperature shift. At the latest time points (10 and 20 days after the shift) cell cycle profiles were similar to those at the higher temperatures (18, 25, and 29 °C) during the proliferative phase. For a further clarification whether S2R+ cells tolerate 14 °C, we analyzed the dynamics of cell proliferation when returning cultures back to 25 °C after 10 days of incubation at 14 °C (Fig. 1E). Cell numbers increased rapidly after the transfer back to the optimal temperature, as fast as in cells never exposed to low temperature, confirming that 14 °C does not result in substantial irreversible damage (Fig. 1E). Moreover, the percentage of dead cells at 14 °C was constant over time and only slightly higher compared to 25 °C (11.9% +/− 5.5 and 8.5% +/− 5.0, respectively, averaged over all replicates and time points from 4 independent experiments, i.e., *n* = 56 and 97, respectively). Overall, these findings suggest that S2R+ cells acclimate after a transfer from 25 to 14 °C and resume cell proliferation at a very low rate eventually.

### Temperature effects on the S2R+ cell transcriptome

**Fig. 2:**
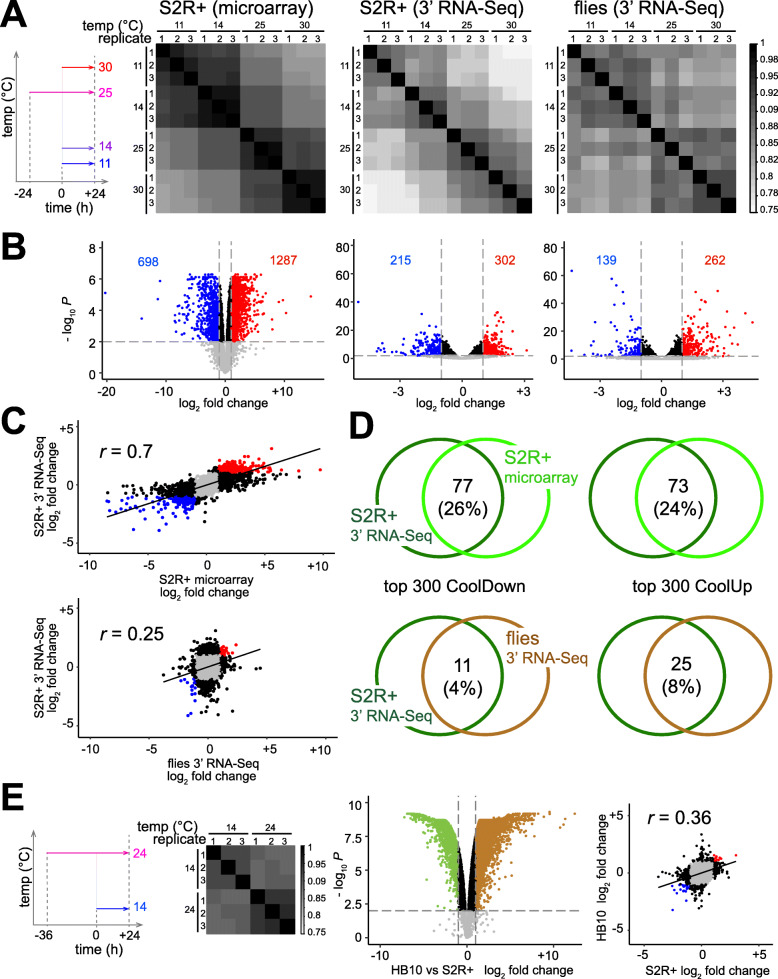
Temperature dependence of transcriptome in S2R+ and HB10 cells and adult male flies. (**A**, **B**) Aliquots of S2R+ cells and adult males were shifted to the indicated temperatures (11, 14, 25 and 30 °C) before RNA isolation (n = 3). A first experiment with S2R+ cells was analyzed with DNA microarrays, a second with S2R+ cells and adult males using 3′ RNA-Seq. (**A**) Pearson’s correlation coefficients after pairwise comparisons revealed maximal similarities between replicates from the same temperature and greater similarity of the transcriptomes at the lower temperatures (11 and 14 °C) compared to the higher temperatures (25 and 30 °C). (**B**) The number of CoolUp genes (blue dots) and CoolDown genes (red dots) with significantly different expression at the lower compared to the higher temperatures (FDR < 0.01; fold change ≥2). (**C, D**) Limited similarity of the transcriptome response to temperature change in S2R + cells and adult male flies. (**C**) Scatter plots of the fold changes of transcript levels (lower versus higher temperatures) either observed in the two experiments with S2R+ cells (top) or in the two 3′ RNA-Seq experiments with S2R+ cells and adult male flies (bottom). *r* = Pearson’s correlation coefficient. (**D**) Overlap among the top 300 CoolUp (left side) and CoolDown (right side) genes either in the two experiments with S2R+ cells (top) or in the two 3′ RNA-Seq experiments with S2R+ cells and adult male flies (bottom). (**E**) Transcriptome changes in response to low temperature in HB10 cells. Culture aliquots were shifted to the indicated temperatures (14 and 24 °C) before expression profiling. Pearson’s correlation coefficients obtained after pairwise comparison of the different samples, a volcano plot and a scatter plot are displayed

To analyze the effects of suboptimal temperatures on the S2R+ cell transcriptome, we used DNA microarrays for initial analyses. Aliquots of cells were plated at different temperatures (11, 14, 25, and 30 °C) (Fig. 2A). Twenty-four hours after the temperature shift, RNA was isolated and analyzed. Three replicate experiments were performed (Supplementary Table S1, Additional File 1). Differences between replicates of the same temperature treatment were minimal, and temperature effects were clearly apparent (Fig. 2A). For a first estimate of the temperature regulated transcriptome, the number of differentially expressed (DE) genes was determined by comparing the average expression level at the high temperatures (25 and 30 °C) with that at the low temperatures (11 and 14 °C). Among 7350 clearly expressed genes, 698 (9%) had increased (CoolUp genes) and 1287 (18%) reduced (CoolDown genes) transcript levels in the cool conditions (fold change ≥2, false discovery rate (FDR) < 0.01) (Fig. 2B; Supplementary Table S2, Additional File 2).

For validation of the microarray data, we repeated the experiment and analyzed transcript levels of selected genes by quantitative reverse transcriptase polymerase chain reactions (qRT-PCR). The results were well correlated with the microarray data (Supplementary Fig. S1, Additional File 3), indicating their reliability.

When mining the microarray data for strong CoolUp genes with plausible acclimation function, we noticed that temperature appeared to affect polyadenylation in ten out of 35 genes. In the ten genes, only the transcript isoforms terminating at the most distal polyadenylation site (PAS) were CoolUp, in contrast to transcript variants resulting from more proximal polyadenylation (Supplementary Fig. S2, Additional File 4). This observation raised the possibility that polyadenylation might be inefficient globally at low temperature, thereby favoring more distal PASs. To clarify temperature effects on alternative polyadenylation (APA), we applied 3′ RNA-Seq [33] after exposure of S2R+ cells to different temperatures (11, 14, 25, and 30 °C) as in the DNA microarray analysis. Moreover, for a comparison between cultured cells and flies, we included RNA samples isolated from adult males after exposure to these temperatures (11, 14, 25, and 30 °C) for 24 h. Before describing temperature effects on APA, we comment on the comparisons of the data from microarrays and 3′ RNA-Seq and on differences between cell types and adult males.

Similar as with the microarray data, the three replicates at a given temperature were generally more strongly correlated than different temperatures in case of the 3′ RNA-Seq data from S2R+ cells (Fig. 2A; Supplementary Table S3, Additional File 5). DE genes were identified as before with the microarray data. Among a total of 7133 clearly expressed genes, 215 (3%) were CoolUp and 302 (4%) CoolDown (Fig. 2B; Supplementary Table S2, Additional File 2). In case of adult males, 139 (1%) CoolUp and 262 (2%) CoolDown genes were found among the 13,168 clearly expressed genes (Fig. 2B; Supplementary Table S2, Additional File 2). Thus, the same arbitrary thresholds (fold change ≥2, FDR < 0.01) resulted in more DE genes in case of the microarray data from S2R+ cells compared to the 3′ RNA-Seq data from both S2R+ cells and flies. The overall correlation between replicates from the same temperature was less pronounced in adult males compared to S2R+ cells (Fig. 2A). However, the difference between the lower temperatures (11 and 14 °C) and the higher temperatures (25 and 30 °C) was also evident in adult males (Fig. 2A; Supplementary Table S4, Additional File 6).

For further comparison, the overall correlation of the observed fold changes of all the genes/probes with clearly detectable expression in the different experiments was determined (Fig. 2C). The comparison between the two experiments with S2R+ cells (analyzed by microarray and 3′ RNA-Seq, respectively) yielded a considerably higher correlation coefficient than the comparison between S2R+ cells and male adult flies (both analyzed by 3′ RNA-Seq and hence not affected by platform-specific differences) (Fig. 2C). This finding emphasized that the transcriptome response to temperature change is strikingly different in adult flies compared to the highly reproducible response in S2R+ cells. This conclusion was further confirmed by the extent of overlap among the top DE genes (Fig. 2D). The overlap among the top 300 CoolDown genes was found to be far greater in case of the comparison of the two S2R+ cell experiments (26%) than the corresponding overlap between S2R+ cells and adult male flies (4%) (Fig. 2D). Comparable findings were also made in case of the top 300 CoolUp genes (Fig. 2D).

Cell-type specificity of the transcriptome responses to low temperatures was further indicated by analysis of an additional cell line, HB10. This cell line had been recently established from transgenic embryos with a method exploiting ubiquitous expression *UASt-ras85D*^*V12*^ driven by *Act5C-GAL4* [34]. Aliquots of HB10 cells were shifted to 14 and 24 °C for 24 h before transcriptome analysis by DNA microarrays (Fig. 2E; Supplementary Table S5, Additional File 7). Although HB10 and S2R+ are both derived from embryos, they are distinct cell lines, as indicated by the comparison of their optimal temperature transcriptomes (Fig. 2E). S2R+ cells are most similar to hemocytes, as previously reported [35]. In contrast, HB10 cells displayed a transcriptome similar to that of adult muscle precursors (AMPs), as typically observed for cell lines generated with this particular method [36, 37]. Illustrating this cell-type difference, the genes for the TFs Pannier (Pnr), Serpent (Srp) and Twist (Twi) were within the top 20 DE genes when comparing S2R+ and HB10 cells at the optimal temperature. Pnr and Srp, which are master regulators of hemocyte development [38], were high in S2R+ cells and low in HB10 cells. In contrast, Twi, which is crucial for mesoderm formation and remains strongly expressed especially in AMPs [37], was high in HB10 cells and low in S2R+ cells. The transcriptome changes in response to temperature downshift to 14 °C were surprisingly distinct in S2R+ and HB10 cells. The overall correlation between the observed fold changes (expression at optimal temperature versus 14 °C) in S2R+ and HB10 cells (Fig. 2E) was almost as low as that between S2R+ cells and flies (Fig. 2C). The overlap among the top 300 CoolUp and CoolDown genes was 20% in both cases. The overlap was further reduced to 1% by filtering for genes that were CoolUp or CoolDown not only in both cell types but also in adult males.

As intended, the 3′ RNA-Seq data allowed clarification whether low temperature globally suppresses polyadenylation efficiency. First, PAS were identified and assigned to genes. Thereafter, genes with multiple PASs were identified (APA genes) (Supplementary Table S6, Additional File 8). The number of APA genes was 2773 in S2R+ cells. These APA genes were associated with two or more of the 14,669 PASs detected in total (Supplementary Fig. S3, Additional File 9). The APA genes represent 37% of the significantly expressed 7495 genes. In case of adult males, the number of significantly expressed genes (10757) and of PASs (22378), as well as the fraction of APA genes (40%, i.e., 4303) was higher than in S2R+ cells, as expected. An identification of genes subject to temperature-dependent APA regulation was performed as described previously [39]. In S2R+ cells, 812 (29%) of the APA genes displayed significant temperature-dependent changes in opposite directions at the two most strongly changing PASs. The genes with temperature-dependent APA were distributed rather equally onto the two classes with either the distal preferred over the proximal PAS at low temperature (class I, 47%) or vice versa (class II, 53%) (Supplementary Fig. S3, Additional File 9). Similar results were obtained with adult males, where 662 (15%) of the APA genes displayed APA regulation by temperature. In this case, 49% of the APA genes were class I and 51% class II (Supplementary Fig. S3, Additional File 9).

In conclusion, temperature change is clearly accompanied by extensive changes in APA in *D. melanogaster*. Moreover, our results indicate that low temperature does not result in a global inhibition of polyadenylation. Preference for the most distal over the most proximal PAS was observed at a frequency comparable to the opposite.

APA usually results in sequence changes in 3′ untranslated regions, potentially altering target sites for miRNAs and RNA binding protein sites with consequences for mRNA stability, translation and localization [40–42]. Some cases of APA also change the coding region. In addition, APA can affect formation of RNA secondary structure, which are of crucial importance for thermosensing in plants, bacteria and viruses [18, 43, 44]. The broad spectrum of potential APA consequences thwarts reliable bioinformatic predictions of physiological effects. Extensive experimental analyses will therefore be required to clarify the physiological significance of the observed temperature effects on APA. Moreover, effects on splicing, which are likely to augment the complexity of the transcriptome response to low temperature, will yet have to be analyzed comprehensively.

### Functional implications of transcriptome responses to low temperature

**Fig. 3:**
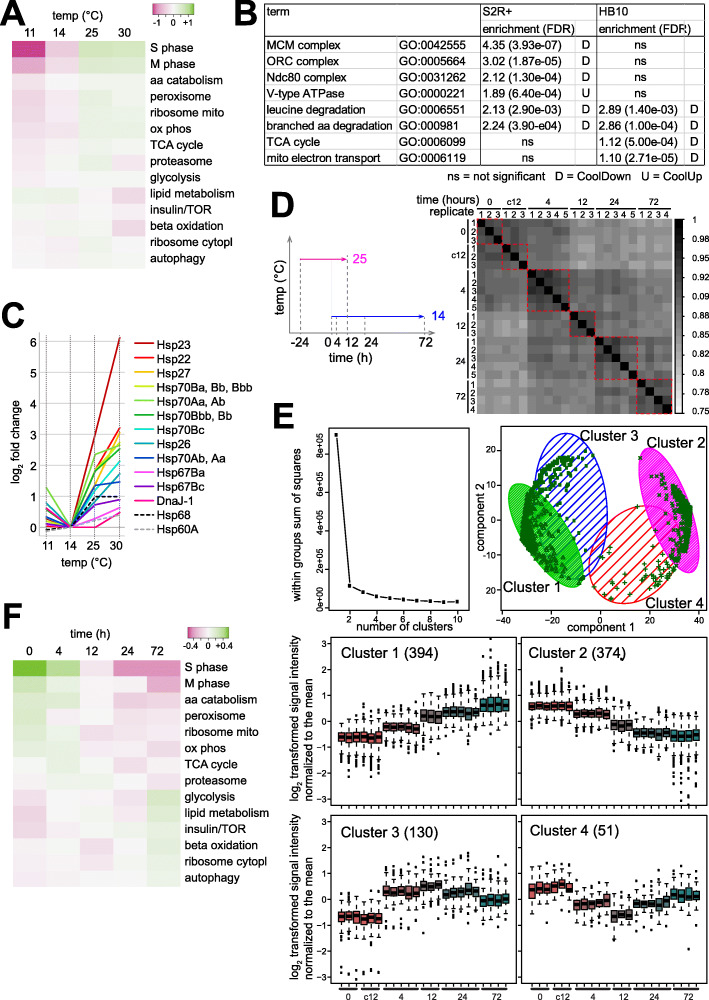
Cool temperature effects in S2R+ cells on cell cycle and stress genes. (**A**) Temperature dependence of transcript levels of genes functionally associated with central cellular processes. Microarray data of the S2R+ cell transcriptome at the indicated temperatures was used for an analysis with curated sets of genes associated with the indicated cellular processes. (**B**) Enrichment of gene ontology (GO) terms by temperature-regulated genes reveal cell-type specific differences between S2R+ and HB10 cells. (**C**) Expression of *Hsp* genes is minimal at 14 °C in S2R+ cells. (**D**) Time course analysis of the transcriptional response to a 25- > 14 °C temperature downshift in S2R+ cells using microarrays. Culture aliquots were analyzed at the indicated times after downshift (0, 4, 12, 24 and 72 h), as well as samples maintained at 25 °C for an additional 12 h (c12). The Pearson’s correlation coefficients obtained after pairwise comparison of the different samples revealed maximal similarities between the three to five replicates from the same time point (red dashed squares). (**E**) Gene clusters with similar temporal expression profiles in response to a 25- > 14 °C temperature downshift identified by k-means clustering using probes with differential expression over time. Plot (top left) obtained by the elbow method [45] after analysis of clusters resulting with increasing *k*. Additional plots describe the clusters obtained at *k* = 4. Temporal profiles of signal intensities revealed steady increase and decrease in cluster 1 and 2, or transient increase and decrease in the two partially separated clusters 3 and 4. Numbers of probes assigned to the four clusters are given. (**F**) Temporal profile of transcript levels from genes with functional association to central cellular processes

Enrichment of functional annotations by DE genes can often provide physiological insights. In case of S2R+ cells, the CoolDown genes were strongly enriched for functional annotations linked to DNA replication, mitosis and cell cycle progression. Indeed, inspection of the data for curated sets of bona fide S phase and M phase genes clearly confirmed reduced transcript levels at 14 °C and even more strongly at 11 °C (Fig. 3A; Supplementary Fig. S4, Additional File 10). Compared to 25 °C, the S and M phase genes were 3.8- and 2.5-fold down at 11 °C on average. The marked downregulation of cell cycle genes at low temperature agreed well with the cell cycle profiles (Fig. 1D), which revealed a marked reduction of the fraction of S phase cells at the corresponding time after shift to 14 °C.

Beyond cell cycle progression, the DE genes regulated by temperature in S2R+ cells did not strongly enrich additional functional annotations. For further corroboration that a temperature downshift to 14 °C does not have pronounced transcriptional effects on central cellular pathways, we analyzed average expression of curated sets of genes with functions in these pathways (Fig. 3A). The results clearly confirmed that other central pathways were far less affected than cell cycle progression by temperature change within the tolerated range (Fig. 3A).

The functional annotations in DE genes further confirmed the high cell-type specificity of the transcriptome response to temperature. In stark contrast to the strong enrichment of cell cycle genes in S2R+ cells, they were not enriched in HB10 cells (Fig. 3B). Similarly, genes encoding subunits of the V-type ATPase were considerably CoolUp in S2R+ cells, but slightly CoolDown in HB10 cells. A further example for a discordant response concerns genes encoding proteins of the cytoplasmic ribosome. Although marginally affected, they were overall CoolUp in S2R+ cells and CoolDown in HB10 cells. Similarly, GO terms associated with the tricarboxylic acid cycle and mitochondrial respiration were clearly enriched among the CoolDown genes of HB10 cells, but not in S2R+ cells. Among GO terms with clear enrichment (enrichment score ≥ 1), only two were found to be shared between S2R+ and HB10 cells, and both these shared terms are linked to degradation of branched amino acids (Leu, Val, Ile). Among the 61 genes subject to CoolUp regulation in both S2R+ and HB10 cells, while not causing strong enrichment of functional associations, we noted the presence of some with crucial roles in the control of glucose and lipid metabolism (like *Adipokinetic hormone* (*Akh*), *Insulin-like receptor* (*Inr*), *Pdk1*, *Hnf4*, *Mef2*, *ewg*/*NRF1*, *Pfrx* [46–49]. Overall, our transcriptome analyses indicate that the effect of temperature on transcript levels is surprisingly cell-type specific. We were unable to identify a set of genes that clearly and invariably responds at the transcriptional level to temperature change within the tolerated range in all three samples (adult male flies, S2R+ and HB10 cells).

To assess the kind and extent of stress that might be triggered in S2R+ cells by a temperature downshift to 14 °C, we focused on known stress response genes. Cellular stress is predicted to increase sharply as temperature goes beyond the readily tolerated range. The heat shock protein genes (*Hsps*) are among the most extensively characterized stress response genes. While originally implicated in the response to high temperature, *Hsps* are also upregulated in response to severe cold in adult *D. melanogaster* [4–8]. Therefore, the temperature-dependence of *Hsp* transcript levels should also be informative concerning the lower bound of S2R+ cells’ tolerated temperature range. If *Hsp* transcript levels were higher at 14 compared to 25 °C, the former temperature would clearly appear as a stressful condition. However, transcript levels of all 12 *Hsp* genes that are induced by heat shocks in cultured *Drosophila* cells [50, 51] were found to decrease not only from 30 to 25, but also from 25 to 14 °C (Fig. 3C; [52]). Strikingly, from 14 to 11 °C, the transcript levels of 10 of the 12 *Hsp* genes increased, rather than dropping further (Fig. 3C). Thus, according to the transcript profiles of *Hsp* genes, 14 °C appears to be within the well-tolerated range, in contrast to 11 °C.

For further exploration of the extent of cellular stress imposed by 14 °C, we performed a time-resolved analysis of the S2R+ transcriptome after a shift from 25 °C to this suboptimal temperature. In the wild, *D. melanogaster* is presumably confronted mostly with relatively slow changes in ambient temperature. Hence, adaptation of *Drosophila* cells for coping with rapid extensive step changes in temperature might be limited. Accordingly, an instant shift of S2R+ cells from 25 to 14 °C is predicted to be stressful initially. However, if 14 °C were indeed within the well-tolerated range, cellular acclimation should succeed eventually, and reduce or even obliterate cellular stress. Genes characterized by a transient transcript increase might therefore represent “stress genes”. In contrast, genes displaying persistent upregulation would qualify as “acclimation genes”; their regulation by temperature might be required for continuous homeostatic cell function at the suboptimal temperature. To study S2R+ transcriptome dynamics after a 25 to 14 °C shift, we isolated RNA at different time points after the shift (0, 12, 24 and 72 h) for probing DNA microarrays. Moreover, cells kept for 12 h at 25 °C rather than shifted to 14 °C were included as well (t12_25 samples). Previous cell counting had indicated that equal cell densities were reached after 12 h at 25 °C and after 72 h at 14 °C, respectively. As some DNA microarrays failed to yield data of acceptable quality, the experiment was replicated so that valid results from at least three and up to five distinct biological replicates were available for each time point (Supplementary Table S7, Additional File 11). Not surprisingly, cells maintained at 25 °C and sampled at t = 0 or 12 h, displayed an extremely similar transcriptome. To increase statistical robustness in the identification of genes responding to the 14 °C shift, we treated the t12_25 data as additional t0 replicates. Compared to this initial time point, a total of 949 probes displayed signals affected by the temperature downshift (fold change > 2 at one or more time points after the shift). We applied k-means clustering of these differentially expressed (DE) probes for an identification of clusters of coregulated genes. As clearly indicated by the elbow method (Fig. 3E) [45], DE probes were segregated into two main clusters, which could not be partitioned readily into additional distinct subclusters. However, enrichment of GO terms associated with the resulting clusters was observed to be maximal at k = 4, providing some physiological support for a division into four clusters.

Among the four clusters, the two major clusters 1 and 2 contained DE probes with an overall gradual increase or decrease of signals after the temperature downshift (394 and 374 probes, respectively) (Fig. 3E). In contrast, the minor clusters 3 and 4 contained DE probes with a transient increase (130) or decrease (51) (Fig. 3E). The analysis of functional associations revealed by far the strongest and highly significant enrichment for cell cycle genes in case of cluster 2 (lowest FDR = 4.15e-26). The transcript levels of bona fide S phase and M phase genes clearly confirmed the downregulation (Fig. 3F; Supplementary Fig. S4, Additional File 10), in further agreement with the initial transcriptome analyses 24 h after a shift to various target temperatures (Fig. 3A). In contrast to cluster 2, enrichment of functional association terms was modest in case of the other three clusters (FDR at least 3.20e-21 fold higher compared to cluster 2). Cluster 1 with gradual upregulation included genes with functions in extracellular matrix, cell adhesion and migration with modest but significant enrichment (FDR < 0.05), consistent with increased spindle shape and clumping of S2R+ cells at 14 °C (Fig. 1B). Cluster 1 also contained *betaTub97EF*, one of the few CoolUp genes common to both S2R+ and HB10 cells, which encodes a beta tubulin isoform shown to stabilize microtubules [53]. However, the GO term “response to stress” was not enriched by the DE probes in cluster 1.

In contrast, stress genes were enriched, although marginally (*p* value 0.0065), in cluster 3 with 95 transient CoolUp genes. Cluster 3 genes implicated in stress included *smp-30*, identified early on as cold stress-induced [54], *Hsp* genes, but only two (*Hsp26*, *DnaJ-1*), as well as *Keap1* (a conserved negative regulator of the response to oxidative stress) [55, 56] and *Ets21C* (induced by the stress-responsive JNK pathway) [57]. Overall, therefore, the clustering results were consistent with an occurrence of transient cellular stress and stress gene upregulation after the rapid step change from 25 to 14 °C. However, the apparent stress response was strikingly limited.

For further assessment of cool temperature stress effects, we focused on stress response genes identified by previous transcriptomic analyses, in which adult flies or larvae had been exposed to various stressors (starvation; oxidative stress: paraquat, hydrogen peroxide, and hyperoxia; endoplasmatic reticulum (ER) stress: tunicamycin; bacterial and fungal infection) [58–61]. Our time course data did not reveal transient or persistent upregulation of such stress response genes (Supplementary Fig. S5, Additional File 12). Similarly, the *Hsp* transcripts overall decreased over time with a minimum at 72 h, the last time point analyzed after the temperature downshift (Supplementary Fig. S5, Additional File 12). We also used antibodies against phosphorylated active forms of JNK and p38a/b MAP kinases, which are well known to be activated in response to many different types of stress including heat shock [62]. A transient activation was observed for both kinases with a maximum at 24 h after the temperature downshift (Supplementary Fig. S6, Additional File 13). In conclusion, S2R+ cells respond to a rapid step change from 25 to 14 °C with a transient induction of stress response pathways. However, at least at the transcriptional level, the accompanying induction of known stress genes remains hardly detectable, indicating that 14 °C is likely still within the well-tolerated range.

Acclimation to 14 °C appears incomplete at 24 h in S2R+ cells according to the temporal dynamics of the apparent stress response. Therefore, we inspected the time course data also for the selected genes acting in central metabolic pathways. This revealed changes in overall expression of genes in several functional networks that continued beyond 24 h (Fig. 3F). However, compared to cell cycle genes, the overall expression changes of genes in other central pathways were clearly more limited.

### Temperature effects on DNA accessibility in chromatin of S2R+ cells

Elegant research with plants has revealed a potentially conserved mechanism for transcriptional control of temperature-regulated genes [19, 20]. Around half of the transcriptome response to temperature was shown to be regulated by nucleosomes containing H2A.Z at the + 1 position in *Arabidopsis thaliana*. Reduction of H2A.Z nucleosomes in mutants resulted in a transcriptome at low temperature that corresponded largely to that normally observed at warm temperature. Similar findings in budding yeast with mutations in the homologous *HTZ1* gene suggested conservation of the crucial role of this histone H2A variant in the control of temperature-regulated genes. To evaluate whether the *Drosophila* homolog His2Av is equally central for transcriptional control in response to temperature change, we depleted it by RNA interference in S2R+ cells and analyzed the effect on the transcriptome after a temperature shift to different temperatures (14, 25 and 30 °C) (Supplementary Fig. S7, Additional File 14). Depletion reduced His2Av to around 30% of normal levels (Supplementary Fig. S7, Additional File 14). His2Av depletion had a pronounced effect on the transcriptome (Supplementary Fig. S7, Additional File 14; Supplementary Table S8, Additional File 15). However, His2Av depletion did not transform the cool temperature transcriptome towards the warm transcriptome (Supplementary Fig. S7, Additional File 14), suggesting that unlike in plants His2Av might not have a prominent role in the temperature-dependent control of transcription in S2R+ cells.

**Fig. 4:**
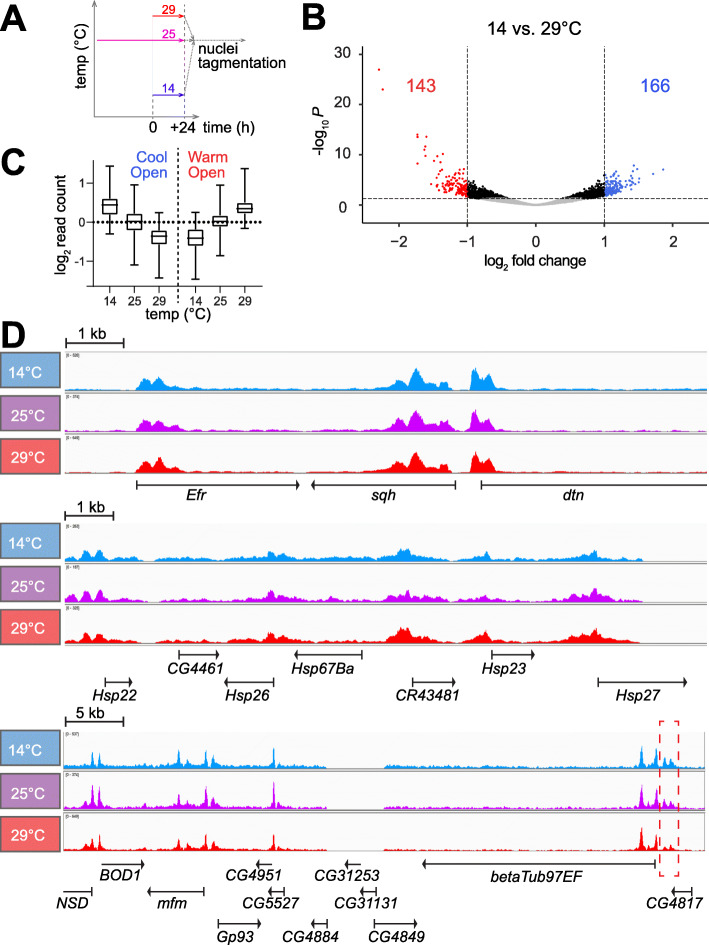
Temperature effects on DNA accessibility in nuclear chromatin of S2R+ cells. (**A**) Culture aliquots were shifted to the indicated temperatures and 24 h later analyzed by ATAC-Seq involving tagmentation in crude nuclei always at 25 °C. Three biological replicates were analyzed. (**B**) Volcano plots illustrate fold changes of read counts in ATAC-Seq peaks when comparing 14 with 29 °C. Peaks with insignificant change (FDR ≥ 0.05) are shown in grey, those with significant but limited change (FC ≤ 2) in black, and those with a strong change (FC > 2) in either blue (CoolOpen) or red (WarmOpen). (**C**) CoolOpen and WarmOpen regions (see panel B) have intermediate accessibility at 25 °C on average. (**D**) Browser tracks display read counts obtained by ATAC-Seq at the indicated temperatures within selected genome regions. While the region shown in the top panel does not include temperature-regulated genes (including *sqh*), the region shown in middle panel contains small *Hsp* genes with transcript levels that were most strongly CoolDown. The bottom panel includes *betaTub97EF* with strongly CoolUp transcript levels [53]. Just upstream of this *betaTub97EF* gene, a CoolOpen region was apparent (dashed red rectangle). In contrast, at most modest accessibility alterations appear to be induced by temperature change in the other regions

To identify genomic regions with temperature-dependent DNA accessibility, we used the assay for transposase-accessible chromatin using sequencing (ATAC-Seq) [32]. S2R+ cells were plated in aliquots at 25 °C and shifted 36 h later for an additional 24 h of growth at either 14, 25 or 29 °C (Fig. 4A). Thereafter, DNA accessibility was probed by tagmentation. All samples were tagmented at 25 °C, avoiding data convolution by temperature effects on enzymatic activity of Tn5 transposase, but precluding detection of potential, rapidly reversible accessibility change. Three replicate experiments were performed. Accessible genome regions were identified as peaks of mapped ATAC-Seq reads. In total, 31,745 peaks and 17,175 consensus peaks (overlapping in at least two of the nine samples) were identified (Supplementary Table S9, Additional File 16). As expected [32], transcription start sites (TSS) were highly enriched within the accessible ATAC-Seq peaks (Supplementary Fig. S8, Additional File 17). For identification of regions with temperature-dependent accessibility, we compared read counts in consensus peaks at 14 and 29 °C. In total, 2317 (12.5%) with a significant difference in accessibility were found (FDR < 0.05) (Supplementary Table S9, Additional File 16). The direction of change was not biased. The numbers of peaks with increased or decreased accessibility at 14 compared to 29 °C were very similar (1189 and 1128, respectively). The numbers of “CoolOpen” and “WarmOpen” regions with strong accessibility changes (FC > 2) were also comparable (166 and 143, respectively) (Fig. 4B). At 25 °C, these regions had an intermediate accessibility overall (Fig. 4C). Moreover, the fraction of consensus peaks with statistically significant temperature-dependent accessibility was dramatically lower when 25 °C was compared to 29 °C (0.03%) or to 14 °C (0.03%), emphasizing that temperature change within the tolerated range results in limited and gradual accessibility differences.

Visual inspection with a genome browser confirmed that the overwhelming majority of the ATAC-Seq peaks were not affected by temperature, as illustrated (Fig. 4D) by a region including the *cis*-regulatory elements (CRE) of the *sqh* gene used for control in subsequent analyses. According to our expression profiling, transcript levels of *sqh* and the flanking genes were at most marginally affected by temperature. However, even in regions with genes generating transcripts with levels responding strongly to temperature, parallel changes in DNA accessibility were rarely evident. The region including the *Hsp23* gene is presented for example (Fig. 4D). This region includes additional *Hsp* genes (like *Hsp22*, *Hsp26*, *Hsp27* and *Hsp67Ba*). As shown (Fig. 3C), all these *Hsp* genes have transcript levels correlated with temperature within the range of 14 to 30 °C; *Hsp23* was in fact the strongest CoolDown gene genome-wide (fold change 14 °C vs 30 °C = 69.7). Nevertheless, DNA accessibility in chromatin did not appear to be affected by temperature in the *Hsp23* region (Fig. 4D). In case of the *Hsp70* genes, which are CoolDown as well (Fig. 3C), we also failed to detect significant accessibility changes (data not shown). Similarly, in case of strong CoolUp genes, a clear majority did not display significant changes in DNA accessibility in parallel with transcript levels. We conclude that with the given sensitivity of our ATAC-Seq analysis the transcriptome response to temperature is not strictly coupled with parallel changes in DNA accessibility at affected genes. However, we definitely also observed examples of correlated changes in transcript levels and DNA accessibility. In case of *betaTub97EF*, which is strongly CoolUp in S2R+ cells [53], an increase in DNA accessibility was detected at low temperature within a region just upstream of the transcriptional start site (Fig. 4D).

### Assay for accurate analysis of temperature effects on CREs

**Fig. 5:**
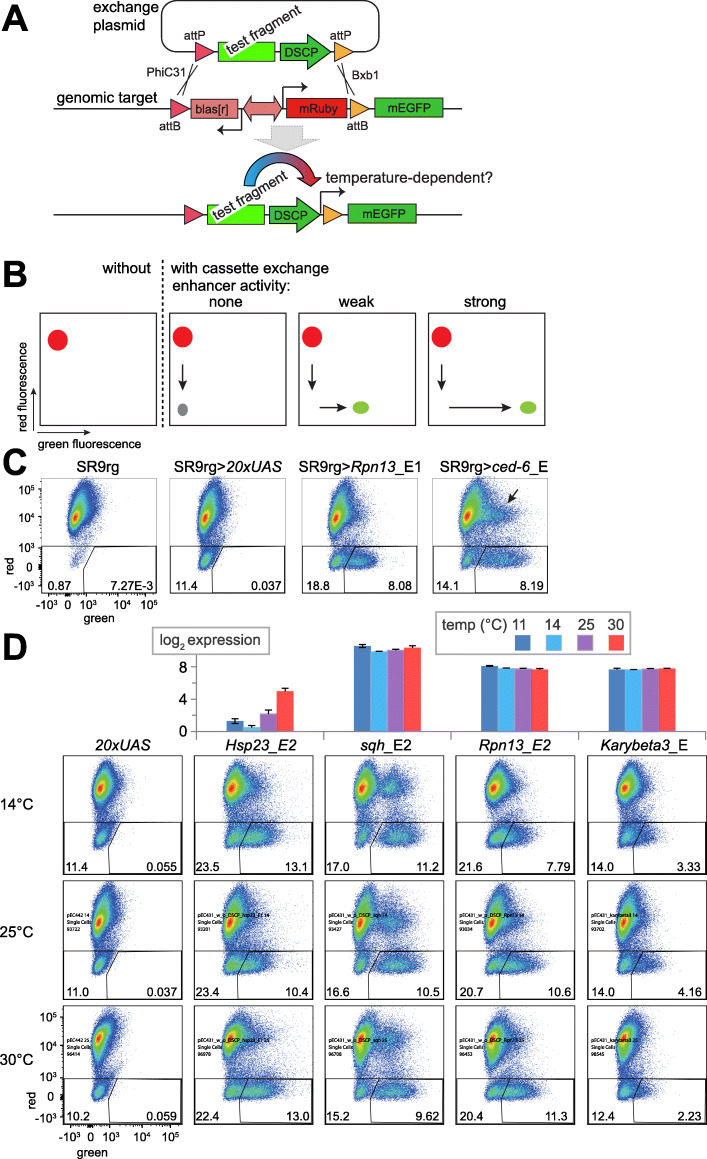
Assay for analysis of temperature dependence of CREs. (**A**) Scheme of assay involving an engineered target locus in SR9rg cells for directional RMCE. After insertion of a candidate CRE into an exchange plasmid and cotransfection with a dual integrase expression plasmid (not shown) for production of the PhiC31 and Bxb1 integrases, the chromosomal cassette (with bidirectional *blas*^*r*^ and *mRuby* marker genes) can be replaced with the exchange plasmid cassette where the CRE is in front of the DSCP promoter. After RMCE, CRE activity can drive expression of green fluorescence in the resulting cell population, which can be cultured in aliquots at different temperatures before analysis by flow cytometry. (**B**) Flow cytometric analysis of enhancer activity of test fragments after RMCE with SR9rg cells. Cartoons of scatter plots with red and green fluorescence intensity along x and y axis depict expected results (from left to right): SR9rg cells before RMCE express mRuby but not mEGFP. RMCE with a test fragment lacking enhancer activity will generate a cell population with neither red nor green fluorescence (grey spot). In contrast, an enhancer fragment will result in a population that expresses only green fluorescence with an intensity depending on enhancer activity. (**C)** Validation of the SR9rg assay system. Scatter plots after flow cytometric analysis (from left to right): SR9rg cells before and after RMCE with the test fragments *20 × UAS*, *Rpn13*_E1 and *ced-6*_E. Analyzed windows and percentage of cells therein are indicated. In the rightmost scatter plot, a population of cells expressing both red and green fluorescence is indicated (arrow). (**D**) Temperature-dependence of CRE activity. Test fragments from genes with temperature-regulated transcript levels (*Hsp23*) or without (*sqh*, *Rpn13*, *Karybeta3*), as indicated by 3′ RNA-Seq (bar diagrams). After RMCE, aliquots of the cells were shifted to 14, 25 or 30 °C before flow cytometric analysis

For a further characterization of the mechanisms controlling the transcriptional response of CoolUp genes, an assay for accurate analysis of temperature effects on CREs appeared to be indispensable. The widely used dual luciferase assay was found to be problematic. The activity of Renilla luciferase expressed from a constitutive promoter (*Act5C* in our case), which is typically used for correction of assay variabilities including transfection efficiencies, was found to depend strongly on the temperature, at which cells were cultured before assaying. As an alternative with hopefully sufficient sensitivity for a detection of the gradual and limited transcriptional changes generally observed within the tolerated temperature range, we generated a cell line, in which GFP reporter transgenes could be assembled efficiently by site-directed chromosomal integration of a test DNA fragment, allowing measurement of its CRE activity by flow cytometry (Fig. 5A). Integration of the test fragment was achieved by directional recombinase-mediated cassette exchange (RMCE) with the two integrases PhiC31 and Bxb1. For RMCE, the test fragment was inserted in front of the *Drosophila* synthetic core promoter (DSCP) [63] between the two distinct integrase target sites. The resulting exchange plasmid was subsequently co-transfected into a special recipient cell line along with a dual integrase expression plasmid (pCo-PhiC31_Bxb1). The recipient cell line (SR9rg), a cloned S2R+ derivative, carried a chromosomal target locus for RMCE, which contained a constitutive mRuby transgene between the two integrase target sites, as well as a promoter-less mEGFP gene on one side just outside of the exchange region (Fig. 5A). Therefore, RMCE with test fragments that have enhancer activity will convert SR9rg cells from red into green fluorescent cells (Fig. 5B). Beyond the chromosomal RMCE target locus, the SR9rg cells were also transgenic for *MtnA_P-cas9*, allowing inducible *cas9* expression by addition of CuSO_4_ to the cell culture medium. Actually, the *cas9* construct had been stably integrated already before chromosomal insertion of the RMCE target locus, because this latter step was intended to be achieved by CRISPR/cas9-directed homologous recombination repair. However, SR9rg cells were found to have off-target integrations of the RMCE target sequences (see Materials and Methods).

For validation that SR9rg cells permit quantitative analyses of temperature effects on CREs, we first generated four exchange plasmids with distinct test fragments (Fig. 5C). A first test fragment (*reg 069*) does not have enhancer activity in S2 cells according to a genome-wide analysis by STARR-Seq and subsequent confirmation with luciferase assays [64]. Similarly, as second fragment (*20 × UAS*) with binding sites for the yeast transcription factor Gal4 was predicted to lack enhancer activity in *Drosophila* cells based on the extensive experience with the GAL4/UAS system in flies [65]. In contrast, the *ced-6*_E fragment has strong enhancer activity according to STARR-Seq and luciferase assays [66]. Finally, a fragment upstream of the *Rpn13* transcription start site was chosen; this region appeared likely to have enhancer activity based on position and accessibility according to ATAC-Seq. Each exchange plasmid was co-transfected along with pCo-PhiC31_Bxb1 into SR9rg cells for RMCE. Four weeks later, the resulting cell populations were analyzed by flow cytometry, along with non-transfected SR9rg cells. As expected, the large majority of the non-transfected SR9rg cells displayed red but not green fluorescence (Fig. 5C). The minor fraction of SR9rg cells without red fluorescence (0.8–2%) might reflect genetic instability, for example loss of the chromosome with the mRuby gene. After SR9rg transfection with only pCo-PhiC31_Bxb1 (but no exchange plasmid), the fraction of cells lacking red fluorescence increased slightly (to about 3%; data not shown), perhaps because of a low level of illegitimate integrase activity [67]. After RMCE with the *reg 069* and *20 × UAS* exchange plasmids, the proportion of cells without red fluorescence was further increased (Fig. 5C, data not shown), indicating that 10% of the analyzed cells were products of successful cassette exchange. A comparable increase in the fraction of cells without red fluorescence was also obtained after RMCE with the *Rpn13*_E1 and *ced-6*_E plasmids (Fig. 5C). Importantly, most cells lacking red fluorescence in these latter samples displayed increased green fluorescence (Fig. 5C). Average green fluorescence in SR9rg > *Rpn13*_E1 was lower compared to SR9rg > *ced-6*_E (Fig. 5C), indicating that enhancer activities can be assessed quantitatively with the cassette exchange system.

In a second validation step, we addressed whether temperature effects on CREs can be analyzed after RMCE with SR9rg cells (Fig. 5D). Temperature dependence of enhancer activity has hardly been studied in *Drosophila*. So far, a bona fide CoolUp enhancer has not yet been described to our knowledge. However, CREs from the *Hsp70* genes were shown to be heat shock responsive. Similarly, temperature dependence of CREs from *Hsp23*, the strongest CoolDown gene in S2R+ cells, has also been characterized to some extent [68]. The *Hsp23* CREs promote increased transcription at elevated temperature [68]. Therefore, an exchange plasmid with a *Hsp23* CRE was generated. For comparison, we used exchange plasmids containing CREs from the loci *Karybeta3, Rpn13* and *sqh*. These genes do not respond to temperature change according to our expression profiling (Fig. 5D). After transfection for RMCE and 3 weeks of culture, the resulting cell populations were split into three aliquots and shifted 24 h later to different temperatures (14, 25 and 30 °C). Flow cytometry was performed 24 h after the shift. In case of the SR9rg > *Hsp23*_E2 cell population, the cells lacking red fluorescence were found to have more intense green fluorescence after incubation at 30 °C compared to 25 °C, and to a minor extent also when compared to 14 °C (Fig. 5D). In comparison, the difference in green fluorescence intensity at 30 compared to 25 °C was less pronounced in the cell populations lacking red fluorescence in case of the CREs from *sqh*, *Rpn13* and *Karybeta3* (Fig. 5D). In conclusion, our validation indicated that the RMCE system might be suitable for an evaluation of temperature effects on CREs. We note that enhancers with an assumed temperature-independent activity might behave as weakly CoolDown in our assay, as suggested by the comparison of green fluorescence intensities at 25 and 14 °C in case of the CREs from *sqh*, *Rpn13* and *Karybeta3*. Alternatively, these enhancers might in fact be weakly CoolDown rather than temperature-invariant.

### A fragment from the *pst* locus confers robust transcriptional upregulation at suboptimal temperature

**Fig. 6:**
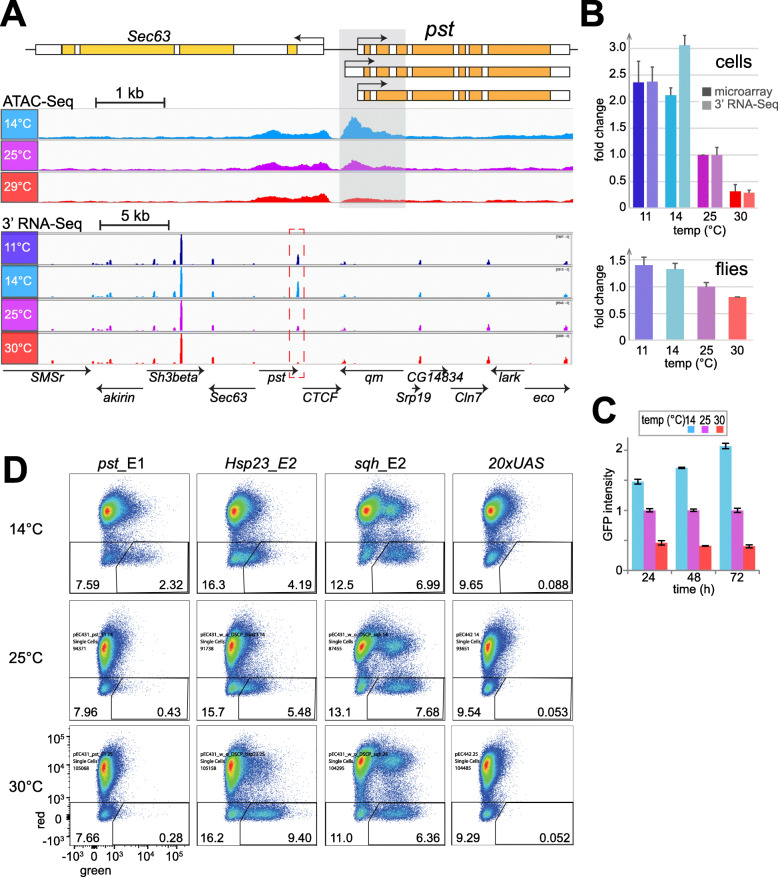
A fragment from CoolUp gene *pastrel* with increased enhancer activity at low temperature. (**A**) Increased DNA accessibility at low temperature in region (grey shading) in the 5′ region of *pst*, a gene with higher transcript levels at low temperature. The genomic *pst* region is shown schematically (top), as well as browser tracks obtained from S2R+ cells at the indicated temperatures (average of three biological replicates) by either ATAC-Seq data (middle) or 3′ RNA-Seq (bottom). (**B**) Quantification of *pst* transcript levels at the indicated temperatures in S2R+ cells (top) and adult male flies (bottom). Results from two independent analyses, by microarray and 3′ RNA-Seq, respectively, are displayed in case of S2R+ cells. The data for flies was obtained by 3′ RNA-Seq. Average of three biological replicates and s.d. are shown, relative to expression at 25 °C, which was set to 1. (**C**) Quantification of EGFP-Pst protein expression levels at the indicated temperatures by flow cytometry. Culture aliquots of S2R + _g-EGFP-*pst* cells were shifted to the indicated temperatures and analyzed at the indicated times after the shift. Average of three biological replicates and s.d. are shown, relative to expression at 25 °C, which was set to 1. (**D**) Temperature dependence of the enhancer activity of the *pst*_E1 fragment (shaded region in panel A) analyzed after RMCE with SR9rg cells. For comparison the fragments *Hsp23*_E2, *sqh*_E2 and *20 × UAS* were analyzed in parallel. After RMCE, cells were shifted eventually to the indicated temperatures and 48 h later analyzed by flow cytometry

RMCE with SR9rg cells was used for an analysis of potential CoolUp enhancers, i.e., enhancers with higher activity at low temperature. Candidate CoolUp enhancer fragments were selected based on our data from expression profiling and ATAC-Seq. For example, ATAC-Seq had revealed a region around the transcriptional start site of *pastrel* (*pst*) with an increased accessibility at 14 compared to 25 and 30 °C (Fig. 6A). The levels of *pst* transcripts were inversely correlated with temperature, in contrast to neighboring genes within the surrounding 40 kb (Fig. 6A, B). This inverse correlation was not only observed in S2R+ cells, but also in adult male flies, where *pst* was also CoolUp but not as pronounced (Fig. 6B). In HB10 cells, however, *pst* did not appear to be affected by temperature. Flow cytometric analyses with stably transformed S2R+ cells expressing an N-terminally tagged EGFP-Pst fusion protein under control of the *pst cis*-regulatory region demonstrated that *pst* expression is also CoolUp at the protein level (Fig. 6C).

To analyze whether the subregion from the *pst* locus that was characterized by increased accessibility at low temperature might confer CoolUp transcription, a corresponding fragment was inserted into the exchange plasmid and used for RMCE with SR9rg cells. The resulting SR9rg > *pst*_E1 cell population was incubated at different temperatures (14, 25, and 30 °C) and analyzed by flow cytometry. In parallel, RMCE with the fragments *Hsp23*_E2, *sqh*_E2 and *20 × UAS* was repeated and the resulting cell populations were subject to identical temperature treatment. The results obtained for the SR9rg > *pst*_E1 cell population revealed an inverse correlation of incubation temperature and green fluorescence in cells lacking red fluorescence. Green fluorescence was more prominent after incubation at 14 °C, compared to 25 and 30 °C (Fig. 6D). Conversely, as previously observed (Fig. 5), *Hsp23*_E2 resulted in increased green fluorescence after incubation at high temperature (Fig. 6D), while the *sqh*_E2 behaved as a strong enhancer largely unaffected by temperature, and *20 × UAS* failed to display enhancer activity at any temperature. The enhancer activity of *pst*_E1 at different temperatures was robust; it was observed in all of a total of 18 independent transfections and flow cytometric analyses. It is concluded therefore that the *pst_*E1 fragment includes CREs that promote increased transcription at low temperature.

Beyond the *pst_*E1 region, we selected 31 additional regions with putative temperature-responsive CREs for analysis after RMCE with SR9rg cells (Supplementary Fig. S9, Additional File 18). Three of these were selected because ATAC-Seq and expression profiling had suggested a potential presence of WarmUp enhancers. Reflecting our main aim, a greater number of regions with potential CoolUp enhancers was selected. Twenty eight such candidate regions were chosen applying somewhat variable criteria: 9 were CoolUp and CoolOpen, 5 were primarily CoolUp and 14 primarily CoolOpen. Several distinct subfragments were tested for some regions. Some fragments were tested in both orientations. Moreover, the DSCP promoter was omitted in case of some of the fragments, which already included an endogenous promoter region. As a result, the total number of assays for identification of enhancers with temperature-dependent activity was 48. The success rate was very low. The majority of the analyzed fragments (32, i.e., 66%), representing 26 distinct regions, displayed no or only marginal enhancer activity. Nine fragments from six distinct regions appeared to have enhancer activity, but only four were clearly temperature-dependent. One of the three regions selected as potential WarmUp CREs functioned as expected (*Prx2540-1*_E1) (Supplementary Fig. S9, Additional File 18). In case of the 28 regions with putative CoolUp CREs, only one (*RNaseX25*) stimulated increased transcription at low temperature. This CoolUp activity was observed with three overlapping fragments (*RNaseX25*_E1, *RNaseX25*_E2 and *RNaseX25*_E3) (Supplementary Fig. S9, Additional File 18).

In conclusion, only two CoolUp enhancers, one from *RNaseX25* and one from *pst*, were identified with our approach.

### The CoolUp CRE from *pst* is regulated by the JAK/STAT pathway and ETS family transcription factors

**Fig. 7:**
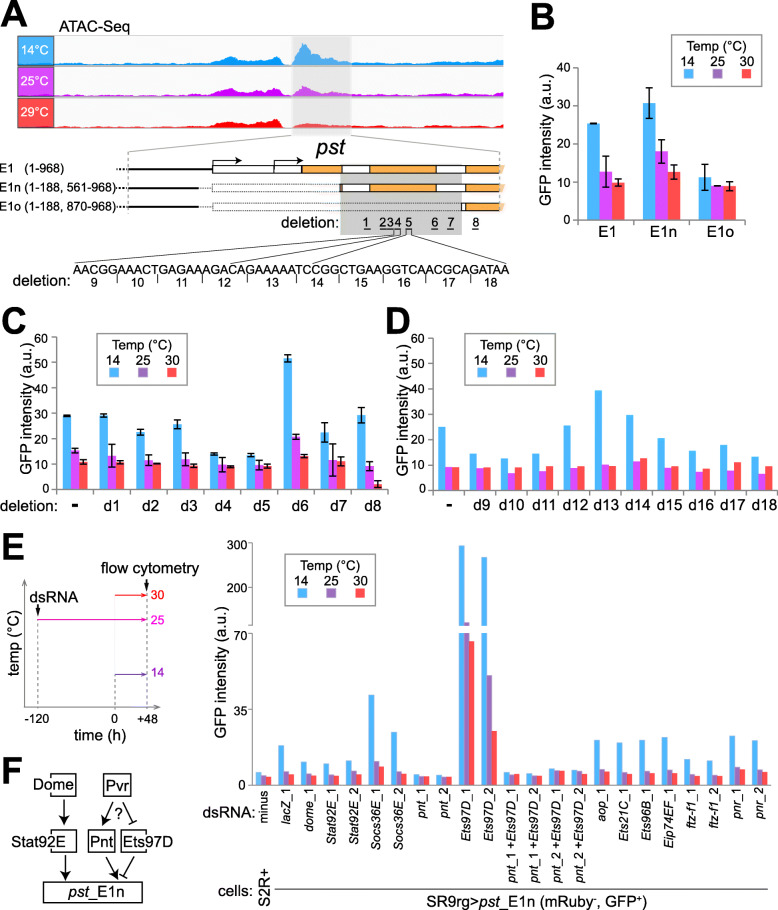
Characterization of the CoolUp enhancer from *pastrel.* (**A**) The CoolUp enhancer activity of the CoolOpen region in the *pst* 5′ region identified by ATAC-Seq (*pst*_E1, light grey shading) was further analyzed with terminal and internal deletion series. Comparison of E1n and E1o revealed an internal region essential for enhancer activity (dark grey shading). The internal deletions d1–8 eliminate predicted transcription factor binding sites. The consecutive 5 bp deletions d9–18 scan the d4-d5 region. (**B-D**) Comparison of enhancer activity of E1 and derived fragments at the indicated temperatures as detected after RMCE with SR9rg cells. Bar diagrams display the median GFP signal intensity in the scatter plot window with cells expressing green but not red fluorescence. Analysis of the fragments E1, E1n and E1o (**B**), E1n derivatives carrying one of the deletions d1–8 (**C**), or one of the deletions d9–18 (**D**). Average of duplicates +/− s.d. shown in (B), and values from a single experiment in (D). (**E**) Characterization of the role of transcription factors (TFs) with predicted binding sites in E1n. The indicated TFs were depleted in SR9rg > *pst*_E1n (mRuby^−^, GFP^+^) cells, followed by a shift of culture aliquots to the indicated temperature and subsequent flow cytometric analysis. Bar diagram represents median GFP intensity of the cell population lacking red fluorescence. In most cases, two independent dsRNA preparations generated from distinct amplicons (*xy*_1 and *xy*_2) were used for depletion. Untreated S2R+ cells and SR9rg > *pst*_E1n (mRuby^−^, GFP^+^) cells treated with *lacZ* dsRNA were used as negative and positive control, respectively. (**F**) Schematic summary model for the control of *pst*_E1 CoolUp enhancer activity. The JAK/STAT signaling pathway (with the transmembrane receptor Dome and the downstream TF STAT92E) acts positively. The TFs Pnt and Ets97D act as competing activator and repressor, respectively, presumably downstream of the Pvr receptor tyrosine kinase

To delineate subregions within *pst*_E1 (968 bp) that are important for increased GFP reporter expression at low temperature, we analyzed a series of *pst*_E1 derivatives with truncations and central deletions after RMCE with SR9rg cells at distinct temperatures (Supplementary Fig. 10, Additional File 19). A central region (372 bp) was found to be dispensable for CoolUp activity, but not the terminal regions (Fig. 7; Supplementary Fig. 10, Additional File 19). Interestingly, CoolUp enhancer activity was also almost completely abolished by a larger central deletion (Fig. 7), indicating that essential enhancer sequences are located within a 308 bp region (Fig. 7, dark grey shading). While this central region is clearly essential, some terminal sequences might still contribute to the overall activity.

Further dissection of the essential 308 bp region was guided by a bioinformatic prediction of binding sites for known transcription factors (TFs) [69], which turned out to be clustered in seven subregions, each approximately 20 bp in length. To evaluate their importance, we analyzed a series of exchange plasmids with corresponding deletions (E1n_d1 to d7). An additional deletion (d8) outside the essential 308 bp region was made for analysis of a predicted STAT92E binding site. Deletion d8 eliminated only 3 bp, comprising the distal half of the STAT92E binding palindrom, in order to keep a predicted overlapping FOXO binding site intact. Flow cytometry indicated that deletion d5 eliminated enhancer activity (Fig. 7). Deletions d7, d4 and d8 also reduced enhancer activity although primarily at low temperature (Fig. 7). Conversely, the d6 deletion increased enhancer activity at all temperatures (Fig. 7).

As the d5 region had proven to be of particular importance, we generated an additional deletion series (E1n_d9 to d18) covering also the adjacent d4 region. Deletions were only 5 bp in length. Deletions d9–11 and d16–18 were found to decrease enhancer activity partially.

Overall, our dissection of *pst*_E1 with terminal truncations and internal deletions of varying size suggested that its CoolUp enhancer activity involves a complex interplay of multiple TFs.

For identification of TFs acting at the CoolUp enhancer from the *pst* locus, we depleted candidate TFs in SR9rg > *pst*_E1n cells by RNAi and analyzed the effects on GFP levels by flow cytometry. We selected TFs with binding sites predicted to be located in functionally important enhancer regions, as well as TFs encoded by genes with temperature-regulated transcript levels (Supplementary Fig. S11, Additional File 20). Since several ETS family members [70] were among the selected TFs (Aop, Ets21C and Ets97D), we included additional ETS proteins with predicted binding sites outside the essential region of *pst*_E1n, if expressed in S2R+ cells (Ets96B, Pnt and Eip74EF). As deletion d8 had supported an involvement of Stat92E, we selected beyond this TF also other proteins acting in the JAK/STAT signaling pathway [71], the transmembrane receptor Dome and the negative feedback regulator Socs36E for depletion experiments.

In a first experiment (Supplementary Fig. S12, Additional File 21), where the SR9rg > *pst*_E1n cells were shifted after four days in the presence of dsRNA to either 14 or 25 °C before flow cytometry, depletion of components of the JAK/STAT signaling pathway (Dome, Stat92E and Socs36E) and of some TFs (Pnt, Ets97D and Ftz-f1) had clear effects on *pst*_E1n enhancer activity. For confirmation, we performed a repetition experiment, in which we also examined additional temperature conditions (14, 25 and 30 °C). Moreover, in case of factors implicated as functionally relevant by the first experiment, we included dsRNA treatment with a second distinct amplicon. Finally, we also performed double depletion of Pnt and Ets97D, because single depletions of these two ETS family members with overlapping predicted binding sites had given antagonistic effects in the first experiment.

The results of the second depletion experiments clearly confirmed those of the first. Accordingly, we conclude that the TFs Ftz-f1, Stat92E and Pnt function as positive, and Ets97D as negative regulator of *pst*_E1n enhancer activity (Fig. 7F). We point out that depletion of these TFs in SR9rg > *ced-6*_E cells, where GFP expression is driven by *ced-6*_E instead of *pst*_E1n, resulted (Supplementary Fig. S12, Additional File 21) in either no effect on GFP fluorescence (in case of Ftz-f1 and Stat92E) or in far milder effects than those observed for *pst*_E1n (in case of Pnt and Ets97B). Thus, the identified TFs (Ftz-f1, Stat92E, and Pnt) presumably act at least to a considerable extent in a direct manner on *pst*_E1n enhancer activity.

The involvement of Stat92E in the control of *pst*_E1n enhancer activity appears to reflect its established function in JAK/STAT signaling. Depletion of additional JAK/STAT pathway proteins also had effects on *pst*_E1n enhancer activity, in directions consistent with their established functions. Dome, the upstream transmembrane receptor, qualified as a positive regulator and the negative feedback component Socs36E as an inhibitor. Expression of *dome* and *Stat92E* is not altered by temperature change in S2R+ cells or at most mildly according to our expression profiling (Supplementary Fig. S11, Additional File 20). In contrast, we note that *Socs36E*, as well as *upd2* and *upd3* are clearly CoolUp (Supplementary Fig. S11, Additional File 20) (and *upd1* expression was absent in S2R+ cells). The three *upd* genes code for the secreted ligand proteins that bind to the Dome receptor and activate JAK/STAT signaling in *Drosophila* [71].

Ets97D appears to counteract the positive regulator Pnt. Ets97D depletion resulted in strong stimulation of *pst*_E1n enhancer activity (about 15 fold at 14 °C) (Fig. 7E). However, if in addition to Ets97D, Pnt was also depleted, enhancer activity was eliminated (Fig. 7E). Therefore, the strong stimulation of enhancer activity resulting from Ets97D depletion is entirely dependent on Pnt. As these two ETS family TFs have overlapping DNA binding specificity [72], we propose that repressive Ets97D prevents binding of activating Pnt by competition for overlapping binding sites. Interestingly, the Pnt-P2 isoform is known to act as a transactivator downstream of various receptor tyrosine kinases (RTKs) (EGFR, Sevenless, Pvr and the FGFRs Heartless and Breathless) also by antagonizing an ETS family repressor, although not Ets97D, but rather Aop/Yan [73]. Depletion of Aop, however, even though effective (Supplementary Fig. S13, Additional File 22) did not affect *pst*_E1n enhancer activity (Fig. 7E; Supplementary Fig. S12, Additional File 21). As the transcripts coding for the Pvr ligands Pvf2 and Pvf3 were CoolUp in S2R+ cells, we analyzed the effect of depletion of Pvf1, 2 and 3 as well as Pvr on *pst*_E1n enhancer activity (Supplementary Fig. S11; Additional File 20). Pvr was found to be required for enhancer activity (Supplementary Fig. S11; Additional File 20), suggesting that Pvr signaling might regulate Pnt activity and thereby also *pst*_E1n enhancer activity.

Overall, the results observed after depletion of TFs with predicted binding sites within *pst*_E1n confirmed that the function of this CoolUp enhancer depends on multiple TFs. Moreover, some of these TFs are likely regulated by signaling pathways that respond to secreted ligands. Depletion of Stat92E, Pnt and Ets97D in SR9rg > *pst*_E1n cells affected the level of transcripts derived from the endogenous *pst* gene in a manner analogous to the effects on the *pst_*E1n EGFP reporter (Supplementary Fig. S13, Additional File 22). However, the effects were less pronounced, indicating the importance of additional regulatory inputs for transcriptional control of the endogenous *pst* gene.

## Discussion

The molecular mechanisms that allow cells in ectothermic animals a successful acclimation to variable environmental temperatures are likely complex. To avoid the complexities potentially caused by cell type- and tissue-specific responses in whole animals, we have focused here on S2R+ cells, a cell line derived from *Drosophila melanogaster* embryos. Moreover, we have studied transcriptional responses to temperatures below the optimum, as these have received less attention than the extensively studied heat shock response. However, rather than effects of extreme cold, our work concerns transcriptional responses to cool temperature within the readily tolerated range. The lower limit of this range in case of S2R+ cells appears to be similar as for flies. For both S2R+ cell proliferation and *D. melanogaster* propagation over generations, the lower limits are at around 14 °C. Based on our analysis of the transcriptome and stress-activated kinases (JNK and p38), a rapid drop in ambient temperature from the optimal temperature (25 °C) down to 14 °C results in only a mild transient stress response in S2R+ cells. Eventual acclimation to this low temperature appears to evolve over several days with mostly gradual increases or decreases, respectively, in the transcript levels of hundreds of “CoolUp” and “CoolDown” genes. While CoolDown genes were mostly cell cycle genes, consistent with an almost complete halt of S2R+ cell proliferation at 14 °C, CoolUp genes serve highly diverse functions. Interestingly, transcriptional changes were not accompanied by evident changes in chromatin organization detectable by ATAC-Seq, except for a minority of the temperature-regulated genes. To identify and characterize *cis*-regulatory elements (CREs) responsible for upregulation of CoolUp genes at 14 °C, a reporter assay was developed where test DNA fragments were chromosomally integrated upstream of the *Drosophila* synthetic core promoter (DSCP) and the EGFP coding region by RMCE in SR9rg cells, an engineered clonal S2R+ derivative. Temperature-dependence of candidate CREs was assessed by flow cytometry after culturing aliquots of the resulting reporter cell population at distinct temperatures. Thereby, only two out of 29 candidate CoolUp CREs were found to result in a robust increase in reporter EGFP expression at 14 °C in comparison to the optimal temperature. By additional characterization of one of these two, a fragment from the *pastrel (pst)* locus, the TFs Stat92E, Pnt and Ets97D were found to be crucial for the function of this CoolUp enhancer. We suggest that the activity of these TFs is controlled by JAK/STAT and RTK signaling pathways.

Some caveats should not be overlooked in the interpretation of our transcriptome data obtained with DNA microarrays and 3′ RNA-Seq. The temperature-associated changes in the level of a specific transcript, as detected by these methods, must not necessarily reflect gene-specific regulation of its rate of transcription. Post-transcriptional effects can modulate the stability and thereby the levels of transcripts. While gene-specific regulation of transcription is generally an important and often dominant mode of control, the evaluation of its significance in acclimation will require more work and attention to issues that are less or even irrelevant during isothermal regulation of biological processes. Temperature change has global effects on DNA and RNA structure. Their magnitude and significance are difficult to predict. Resulting or independent global effects on rates of transcriptional initiation and elongation with secondary effects on availability of RNA polymerase, splicing and termination might be associated with temperature change. Although our findings concerning *pst* argue that gene-specific transcriptional regulation contributes to acclimation, global effects and post-transcriptional levels might be important as well.

While intriguing, the similarity of what we have designated the “readily tolerated temperature range” in S2R+ cells and flies should not be overrated. The genetic stability in *Drosophila* cell culture lines is clearly lower than in flies. Genome resequencing of close to twenty distinct cell lines has exposed extensive copy number changes [74]. While mostly cell line-specific, some changes were common to many lines, including a high copy region encompassing *Pvr*, which has been suggested to reflect selection for provision of protection against apoptosis by this gene. Clearly, drift and altered selection pressure during S2R+ cell propagation might have modified some of the responses to temperature. However, *Drosophila* cell lines retain characteristics of the tissue of origin according to transcriptome comparisons between cell lines and tissues [35]. Accordingly, the low similarity in the transcriptional response to temperature between adult male flies, S2R+ and HB10 cells that we have observed, might reflect genetic or epigenetic differences or both. It will be of considerable interest to corroborate the apparent cell-type specificity in the transcriptome response to cool temperature with future direct comparisons between distinct tissues and cell types of the organism. Technically, single cell RNA-Seq methodology should ease such future experiments. In any case, our comparisons indicate a high plasticity in this response, precluding instant wide-ranging generalizations. Our 3′ RNA-Seq analyses with S2R+ cells and adult males, as well as previous RNA-Seq studies with adult flies [27, 28, 30], emphasize that acclimation within the readily tolerated temperature range is accompanied by transcriptome changes beyond transcript abundance. Extensive changes in alternative mRNA splicing and polyadenylation further increase the response complexity.

Based on the transcriptional response of genes previously shown to be strongly induced by various types of stress (heat, oxidative, ER failure, infection and starvation), incubation of S2R+ cells at 14 °C appears to be at most mildly stressful. However, progression through the cell cycle was severely inhibited after a shift to 14 °C. Transcript levels of S- and M phase genes were strongly decreased and flow cytometry revealed a transient G2 arrest. S2R+ cells also stall in G2 when reaching high density during growth at the optimal temperature. Transient cell cycle arrest in G2 is frequently observed in *D. melanogaster* during normal development and in response to stress [75–79]. In contrast to S2R+ cells, cell cycle genes were not downregulated when HB10 cells were grown at 14 °C. This cell line, which we have generated from embryos using transgenic transformation with activated Ras [80], has a considerably higher cell doubling time at the optimal temperature compared to S2R+ cells. As HB10 cells were generated recently, they have not been exposed to selection for rapid proliferation in culture as intense as the S2R+ cells, which have been passaged during the past 20 years. The distinct history might well be responsible for the striking difference in temperature-dependence of cell cycle gene expression in these two cell lines. It is not excluded, however, that cell-type differences contribute as well. S2R+ cells are most similar to hemocytes and HB10 cells to adult muscle precursors. Importantly, the differences in the temperature-dependent transcriptome of S2R+ and HB10 cells go far beyond cell cycle genes.

To evaluate whether temperature-dependent transcriptome change is linked to alteration of DNA accessibility in chromatin, we have applied ATAC-Seq after incubation of S2R+ cells at different temperatures. Around 2300 ATAC-Seq peaks were significantly affected by temperature when comparing 14 with 29 °C. Although numerically greater than the number of temperature-regulated genes, such differentially accessible regions were often conspicuously absent from chromosomal locations with differentially expressed genes. The extensive differences in *Hsp* transcript levels for example were associated with at most marginal changes in chromatin accessibility. This observation is in full agreement with the findings concerning the molecular mechanisms, which mediate heat shock-induced upregulation of *Hsp70* expression [2]. Extensive analyses have clearly established that the regulated step consists in the release of RNA polymerase II from a promoter-proximal pause site into productive elongation. While the recruitment of RNA polymerase is linked to prominent chromatin opening within the *cis*-regulatory region around the TSS, all subsequent steps (initiation, pausing, release and elongation) occur with minimal changes in DNA accessibility [2]. The *Hsp70* promoter region is therefore already highly accessible in the non-induced state. This paradigm is known to govern the regulation of very a substantial fraction of all genes [2, 81]. Conceivably, it applies to many of the genes that are subject to temperature-dependent regulation within the range of 14–30 °C. Although we failed to detect significant changes in DNA accessibility at most of the temperature-regulated loci, there were also clear exceptions which displayed correlated changes in DNA accessibility and transcript levels in response to temperature change. Moreover, in case of the *pst* locus, a region with CoolUp DNA accessibility was also found to have CoolUp enhancer activity.

For precise quantitative analyses of temperature effects on the activity of candidate CREs, we have developed a novel reporter assay. In this assay, DNA fragments of interest are first inserted into an exchange plasmid, which is then co-transfected with an integrase expression plasmid for RMCE-mediated transfer of the fragment into engineered target sites in the genome of SR9rg cells. This targeted integration strategy increases assay reproducibility compared to analyses after transient transfection with or without selection of random integrations. With our assay, comparison of an original with a mutated derivative of an enhancer fragment for example is far less convoluted by unavoidable variabilities in transfection efficiency, integration site and copy number. Recently, several similar *Drosophila* cell lines allowing targeted RMCE-mediated integration of DNA fragments were described [67, 82–84]. These cell lines carry single target site for non-directional RMCE. In contrast, our SR9rg cell line has multiple target sites, which can be disadvantageous for certain applications, but our target sequence includes special features designed for enhancer analysis. Directional RMCE provides control over fragment orientation. Moreover, since the EGFP reporter coding region is part of the target site and hence absent from the exchange plasmid for test fragment delivery, random off-target integrations of this exchange plasmid cannot contribute to reporter expression. Therefore, no selection against such variable off-target integrations is required. This is highly advantageous because in cultured *Drosophila* cells random integrations occur frequently and methods for selection against them are inefficient [67, 82] (Y.B and C.F.L., unpublished observations). For analysis of CREs, the design of our target site results in the following constraints. For analyses of enhancer activity, the positioning of test fragments downstream of the reporter gene is usually preferred. In our assay system, test fragments are necessarily upstream, as we aimed for an approach that permits an analysis also of promoter activities. However, if the test fragment includes a TSS and a downstream ATG motif, the latter needs to be in frame with the EGFP coding sequence and free of downstream stop codons, or else potential transcriptional stimulation by the test fragment cannot be detected. Problems with TSSs followed by out-of-frame ATGs and premature stop codons might be bypassed by inversion of a given test fragment in front of the DSCP, although interference by promoter competition might then arise. The fraction of putative CoolUp CREs that might have compromised our assay because of promoter competition is likely substantial, as our selection of candidate CRE fragments was based to a large part on ATAC-Seq peaks covering the region with annotated TSSs. We suggest that the observed low rate of successful validation of putative CoolUp CREs is explained in part by such technical problems.

Apart from some limitations as discussed, our reporter assay involving flow cytometric EGFP signal quantification with SR9rg cells after integration of test DNA fragments by RMCE is highly reproducible. Moreover, by incubation of culture aliquots at different temperatures before flow cytometry, temperature-dependence of CREs can be assessed accurately. Thereby, we have been able to demonstrate that the *pst*_E1 fragment stimulates EGFP reporter expression more effectively at 14 °C than at 25 and 30 °C. We are not aware of any other CoolUp enhancers previously identified in *D. melanogaster*, and the number described in other animal species appears to be low [85, 86]. By further analysis with the *pst*_E1n subfragment, we have uncovered an involvement of the JAK/STAT pathway and the ETS family proteins Ets97D and Pnt in the function of this CoolUp enhancer. In particular, Ets97D and Pnt depletion resulted in striking but opposite effects. Ets97D depletion boosted enhancer activity strongly, while Pnt depletion abolished it, as also double depletion of Ets97D and Pnt. Therefore, Ets97D appears to act as a repressor at *pst*_E1n that prevents the activator Pnt from inducing transcription by competition for the overlapping binding sites.

Pnt is known to function as a key TF activated by RTK signaling [87–89]. *Drosophila* RTKs (including EGFR, Heartless/FGFR, Breathless/FGFR, Torso, Sevenless) signal via the ras/MAP kinase cascade, resulting in activated MAPK, which phosphorylates the Pnt-P2 isoform. Thereby Pnt-P2’s function as a transcriptional activator is stimulated. Moreover, activated MAPK also phosphorylates the ETS family protein Aop/Yan and thereby inhibits Aop/Yan’s repressive Pnt-P2-antagonizing function. The role of Aop/Yan appears to have been taken over by Ets97D in case of *pst*_E1n. Ets97D (also named D-elg or Delg) has been proposed to function as a subunit of a TF complex homologous to mammalian NRF-2/GABP which has been implicated primarily in the regulation of housekeeping genes involved in ribosomal and mitochondrial biogenesis [90]. The proposal that *Drosophila* Ets97D corresponds to GABPα is supported by functional characterizations, which have also revealed an Ets97D requirement for the growth promotion of Cyclin D-Cdk4 [91, 92], as well as for normal oogenesis [93]. The apparent growth-promoting role of Ets97D is intriguing in the context of temperature acclimation. Interestingly, Pnt has also been implicated very recently in the control of metabolic gene expression [94].

Future work will be required for an identification of the cellular “thermometer” acting at the top of the pathways responsible for the increased activity of the *pst*_E1n CoolUp enhancer at low temperature. The endogenous *pst* gene is also expressed at higher levels at low temperature, as shown by our analysis of transcript and protein levels. Depletion of proteins crucial for *pst*_E1n reporter expression was found to have the same effects on *pst* transcript levels, although less pronounced and with some exceptions (*Socs36E* and *Pvr*). Thus, its transcriptional control might involve some additional regulatory inputs beyond those acting on *pst*_E1n.

The function of *pastrel* (*pst*) is not understood at the molecular level. The gene was named after one of Pavlov’s dogs because of an olfactory conditioning defect associated with a P element insertion in *pst* [95]. Knockdown of *pst* in larval muscles impairs the formation of neuromuscular junctions [96]. More recently, *pst* function has also been linked with susceptibility to infection with *Drosophila* C Virus (DCV) [97]. The *pst* gene appears to be highly polymorphic in natural populations, and some of these polymorphisms are associated with either increased or decreased DCV susceptibility [98]. Genes with sequence similarity to *pst* can be detected in insects but not in vertebrates. By analysing the Pst amino acid sequence using I-TASSER [99], we obtained evidence for structural similarity with Vps35, a subunit of the retromer complex, which is involved primarily in selection of transmembrane proteins for transport between endosome and trans Golgi network or plasma membrane. We have made an initial attempt to assess *pst* function in acclimation of *D. melanogaster* to cool temperatures by generating null alleles using CRISPR/cas. Somewhat surprisingly, these mutations did not prevent development into fertile adults and did not cause obvious developmental cold sensitivity. Clearly, additional work will be required to resolve whether and how *pst* contributes to acclimation to low temperature.

## Conclusions

We are providing a rich data resource for future analyses of the transcriptional regulation of genes within the readily tolerated range in the ectotherm animal *D. melanogaster*. Moreover, we have developed a novel reporter assay that permits the characterization of the temperature dependence of the activity of CREs. Our identification and functional dissection of the *pst*_E1 enhancer demonstrates the utility of data resources and assay. Our results concerning the function of this CoolUp enhancer provides initial mechanistic insights into transcriptional upregulation induced by a shift to temperatures at the lower end of the readily tolerated range.

## Methods

### Cell culture

S2R+ cells [31] and S2R + _*MtnA*p-*His2Av*-mRFP_hygro cells [100] were cultured in Schneider’s medium (Gibco, cat# 21720, Thermo Fisher Scientific, Waltham, MA), supplemented with 10% fetal bovine serum (Gibco, cat# 10500–064) and 1% Penicillin-Streptomycin (Gibco, cat# 15140). Cells were cultured at 25 °C, unless otherwise noted. The number of live and dead cells was determined with an automated cell counter (Countess, Invitrogen, Thermo Fisher Scientific, Waltham, MA) after harvesting cells with 1 ml trypsin-EDTA (Gibco, cat# 25050–014) and staining with trypan blue (0.2% final concentration).

For the analysis of temperature effects on the proliferation of S2R+ cells, we plated aliquots of S2R+ cells (2 ml of suspension with 1 × 10^5^ cells/ml) in round cell culture dishes (35 mm diameter). Four aliquots were plated for each time point and temperature to be analyzed. Two of these aliquots were eventually used for cell counting and for the analysis of the cell cycle profile by flow cytometry. The other two aliquots were used for live imaging by phase contrast microscopy. All aliquots were first incubated for 24 h at 25 °C. Thereafter (t = 0), aliquots were shifted into incubators pre-equilibrated to different temperatures (14, 18, 25 and 29 °C) and incubated until analysis at the chosen time points. For experiments where temperature effects were analyzed only by phase contrast microscopy, cells were plated in 24 well plates, one plate for each temperature (9, 11, 13, 15 and 17 °C) in three wells per plate.

The cell line HB10 was generated as described [80] by dissociation of embryos that were collected for 2 hours and aged for 6 hours at 25 °C. The embryos were collected from a cross of *yw*; *P{UAS-Ras85D.V12}2*; *P{CaSpeR-CoStart_attP_STOP}32.1* males with *yw*; *P{w[+mC] = Act5C-GAL4}17bFO1*/*TM6B, Tb* virgins. Fly lines with *P{UAS-Ras85D.V12}2* (# 64196) and *P{w[+mC] = Act5C-GAL4}17bFO1* (# 3954) were obtained from the Bloomington *Drosophila* Stock Center (Indiana University, Bloomington, IN, USA). HB10 cells were grown in the same culture medium as the S2R+ cells and passaged once or twice each week. The HB10 cells were used for expression profiling after 31 passages.

S2R+ cell transfections were performed using FuGENE HD (Promega, cat# E2311) in 6 well plates or 25-cm^2^ flasks. In a 25-cm^2^ flask, 2.6 × 10^6^ cells were plated in 4 ml complete medium and incubated at 25 °C. One hour after plating, 200 μl transfection mix (2 μg plasmid DNA and 8 μl FuGENE HD in Schneider’s medium) was added. To establish stably transfected cell lines, either 25 μg/ml blasticidin or 300 μg/ml hygromycin was added 2 days after transfection, unless otherwise noted. S2R + _EGFP-pst cells were generated by selection of stable integrations after transfection with pCaSpeR4-gEGFP-pst-blas^r^ (see below), and similarly, SR9 cells, which express *cas9* under control of the *MtnA* promoter, after transfection with pMT-cas9-hygro (see below). For induction of *cas9* expression, CuSO_4_ was added to the culture medium (final concentration 500 μM) 24 h before transfection with gRNA and repair template plasmids (see below).

Single cell cloning was done with S2R+ feeder cells in 96 transwell co-culture plates (Corning, cat# CLS3380). First, 150 μl of a S2R+ cell suspension (3 × 10^5^ cells/ml) were plated in the bottom compartments below insert. Eighty μl of complete medium were then added into each insert. After 24 h at 25 °C, single cells were sorted by fluorescence-activated cell sorting (FACS) into the inserts. To minimize evaporation, plates were closed with parafilm and cultured in a plastic box with moistened tissue paper. When the feeder cells reached confluency, the upper insert plate was transferred onto a plate with fresh feeder cells prepared in an accessory plate (Corning, cat# CLS3382). Complete medium was added to the upper inserts every few days. When the clones had reached 100% confluence, cells were harvested by pipetting up and down. For expansion of the clonal population, cells were first transferred into a well of a 24-well plate and cultured in complete medium. Cloning efficiency after 8 weeks incubation was 40–50%. The characterization of the SR9rg clonal line is described in detail below.

Transfection of SR9rg for RMCE was performed in 6-well plates. 1 × 10^6^ cells were plated in 2 ml complete medium and incubated for 1 h at 25 °C before addition of 100 μl of transfection mix (500 ng exchange plasmid and 500 ng pCo-Bxb1_PhiC31, 4 μl FuGENE HD in Schneider’s medium). Whenever the transfected cells reached 100% confluence, they were passaged (1:4–5). Three weeks post-transfection, cells were collected and resuspended in complete medium at a concentration of 1 × 10^6^ cells/ml. Thereafter, aliquots of cells were plated in 6-well plates. The number of cells seeded was adapted to the temperature, to which they were shifted eventually (30 and 25 °C: 1 × 10^6^ cells; 14 °C: 2 × 10^6^ cells) in order to generate comparable cell densities at the time of cell harvesting. After seeding, the cells were cultured for an additional 24 h at 25 °C before shifting to either 14, 25 or 30 °C. After 48 h of incubation at these variable temperatures, cells were harvested and re-suspended in phosphate buffered saline (PBS) (Gibco, cat# 10010–015). Cells were filtered with a Cell-Strainer (Falcon, cat# 352235) and kept on ice until analysis by flow cytometry.

Depletion of candidate transcription factors with predicted binding sites within *pst*_E1n was performed with SR9rg > *pst*_E1n (mRuby^−^, GFP^+^) cells. This cell population was obtained by FACS (see below), selecting cells with significant levels of green fluorescence and only background levels of red fluorescence from SR9rg > *pst*_E1n cells. After sorting by FACS, the selected cells were expanded at 25 °C and frozen in aliquots. Depletion of the JNK protein kinase encoded by the *basket* (*bsk*) gene in S2R+ cells and treatment with bacterial lipopolysaccharides (LPS) were done as described previously [52].

### Flow cytometry and fluorescence activated cell sorting

Fluorescence activated cell sorting (FACS) and flow cytometry were carried out at the Cytometry Facility at the Irchel campus of the University of Zurich.

For determination of the cell cycle profiles of S2R+ cell populations after incubation at different temperatures, cells were harvested and fixed with 95% ethanol. Fixed cells were stored at 4 °C (for maximally 3 weeks) before analysis by flow cytometry. Cells were resuspended in 1 ml PBS. To degrade RNA and stain DNA, 25 μl of RNase A stock solution (1 mg/ml) and 25 μl of propidium iodide stock solution (1 mg/ml, Fluka, cat# 81845) were added. After incubation at 4 °C overnight, flow cytometric analysis of DNA content was completed using a BD FACSCanto instrument. PI was excited with a 561 nm laser and emission was detected using a 575/26 nm band pass filter. The resulting cell cycle histograms were analyzed with the software FlowJo (Treestrar Inc.).

For single cell cloning, cells were sorted with a FACSAria III cell sorter (BD Biosciences) using a 100 μm nozzle into 96 transwell co-culture plates. The same instrument and nozzle were also used for the isolation of SR9rg > *pst*_E1n (mRuby^−^, GFP^+^) cells into tube (Falcon, 352,058) and plated in a 24-well plate for expansion of the population. Red fluorescence was excited with a 561 nm laser and emission was detected using a 610/20 nm band pass filter. Green fluorescence was excited with a 488 nm laser and emission was detected using a 530/30 nm band pass filter.

To determine enhancer activity after RMCE with SR9rg cells, we analyzed 1 × 10^5^ single cells for each sample using an LSR II Fortessa instrument (BD Biosciences). For quantification of the data obtained with *pst*_E1n and its derivatives, we used the software FlowJo. First, we defined a gate for the mRuby-negative population based on the data obtained with S2R+ cells (mRuby-negative) and SR9rg cells (mainly mRuby-positive cells). Second, we defined a gate for GFP-positive cells (gate 2) and a gate for GFP-negative cells (gate 3) based on the data observed after RMCE with *20 × UAS* which do not contain GFP-positive cells. The median GFP signal intensity of the cells within gate 3 was used for background correction. It was subtracted from the median GFP signal intensity of the cells within gate 2, yielding the final value representing enhancer activity used for the bar diagrams.

### RNA interference

DNA templates for production of dsRNA by in vitro transcription were amplified enzymatically from genomic DNA of S2R+ cells with primers that introduce terminal T7 RNA polymerase promoter sequences (Supplementary Table S10, Additional File 23). The DNA template fragments were purified from agarose gel using gel extraction kit (QIAGEN, cat# 28706) and used for in vitro transcription with the Ambion T7 Megascript Kit (Invitrogen, cat# AM1334). dsRNAs were precipitated by adding 3.3× volumes of 100% ethanol, followed by chilling overnight at − 20 °C. After a centrifugation (13,100×g, for 15 min at 4 °C), the supernatant was discarded. The retained pellet was washed with 75% ethanol, air-dried and dissolved in RNase-free water. The concentration of dsRNA in the final samples was determined using Nanodrop (Thermo Scientific, cat# ND-ONEC-W).

Depletion of candidate transcription factors involved in the function of the *pst*_E1n enhancer fragment was performed with SR9rg > *pst*_E1n (mRuby^−^, GFP^+^) and SR9rg > *ced6*_E (mRuby^−^, GFP^+^) cells. First, cells were harvested and resuspended in Schneider’s medium (without serum and other additives) at a density of 1.5 × 10^6^ cells/ml. 1.5 ml cell suspension were mixed with 15 μg dsRNA and seeded into a well of a 6-well plate. After an incubation of 45 min, 3 ml complete medium were added, followed by gentle mixing. After 4 days of incubation with dsRNA, cells were harvested and resuspended in complete medium at a concentration of 1 × 10^6^ cells/ml. Thereafter, aliquots of cells were plated in 6-well plates. The number of cells seeded into a well was adapted to the temperature, to which they were shifted eventually (30 °C: 1 × 10^6^ cells; 25 °C: 1 × 10^6^ cells; 14 °C: 2 × 10^6^ cells). After seeding, the cells were cultured for an additional 24 h at 25 °C before shifting to either 14, 25 or 30 °C. After 48 h of incubation at these variable temperatures, cells were harvested and resuspended in PBS (Gibco, 10,010–015). Cells were filtered with a Cell-Strainer (Falcon, 352,235) and kept on ice until analysis by flow cytometry.

### Immunoblotting

Total cell extracts were prepared in 3x Laemmli buffer, heated for 5 min at 95 °C and cleared by centrifugation at 4 °C (3 min, 17,000×g). Aliquots were frozen in liquid nitrogen and stored at − 70 °C. Protein concentration was determined with the Pierce 660 nm Protein Assay (Thermo Fisher Scientific, cat# 2262). Proteins were resolved by standard SDS polyacrylamide gel electrophoresis using pre-stained PAGE RULER plus (Thermo Fisher Scientific, cat# 26619) molecular weight markers. Proteins were transferred onto nitrocellulose membranes by electrotransfer with a tank system. Membranes were transiently stained with Ponceau S to confirm successful transfer. After blocking, membranes were probed with the following primary antibodies: rabbit anti-JNK (Santa Cruz Biotechnology, Inc., Heidelberg, Germany, cat# sc-571, 1:1000), rabbit anti-phospho-JNK 81E11 (Cell Signaling Technology, Leiden, Netherlands, cat# 4688, 1:1000), rabbit anti-phospho-p38MAPK (Cell Signaling Technology, cat# 9211, 1:400), mouse anti-alpha-tubulin DM1A (Sigma Aldrich Chemie GmbH, Buchs, Switzerland, cat# T9026, 1:50′000), mouse anti-PSTAIR (Sigma Aldrich Chemie GmbH, cat# P7962, 1:50′000), mouse anti-FLAG M2 (Sigma, cat# F1804, 1:1000) and rabbit anti-mCherry (1:1000) [101]. As secondary antibodies we used horseradish peroxidase conjugated goat IgG anti-rabbit and anti-mouse IgG (H + L) (Jackson ImmunoResearch Europe Ltd., Cambridge, UK, cat# 111–035-003 and 115–035-003, 1:1000). Signals were detected by chemiluminescence. Signals corrected by subtraction of local background were quantified using ImageJ and compared based on linear interpolation of intensity values obtained with the dilution series that was resolved in parallel.

### qRT-PCR

Total RNA was extracted from cultured cells with TRIzol (Invitrogen, cat# 15596026), followed by DNase digestion (DNA-free DNA Removal Kit, Ambion, cat# AM1906). Alternatively, we also used Direct-zol™ RNA MiniPrep Plus kit (ZYMO, cat# R2070, Lucerna-Chem AG, Lucerne, Switzerland) for RNA preparation. cDNA synthesis was performed using Transcriptor High-Fidelity cDNA Synthesis Kit (Roche) with 500 ng RNA per reaction. Quantitative real-time PCR was performed using SYBR Green with an Applied Biosystems 7900HT using the recommended two-step cycling protocol. Alternatively, the QuantStudio™ 3 Real-Time PCR System (ThermoFisher, cat# A28137) was used.

For validation of the microarray data from experiment M1 (see below), we selected some genes for analysis by qRT-PCR as follows. Among strong ColdUp genes, the top hit (*CG32944*) was chosen and four genes encoding proteins with predicted functions (*CG6321* and *Orct2* as transporters, *Lip4* as lipase, *CG32944* as protein kinase). Among strong HeatUp genes, we selected the top hit (*Hsp23*) and two additional genes with known functions (*Hsp22* and *DNApol-alpha50*). In addition, we selected two invariant genes with known crucial roles in autophagy and growth regulation (*Atg1* and *PTEN*). For normalization, we used primer pairs for three genes, *Act5C*, *alphaTub84B* and *Tbp*. Primer sequences are given in Supplementary Table S10, Additional File 23.

### Plasmid constructions

All PCRs for cloning were performed with Phusion High Fidelity DNA polymerase (NEB, cat# M0530) using the default buffer (NEB, cat# B0518S).

For generation of pMT-cas9-hygro^r^, we first introduced a ClaI restriction site into pMT-hygro^r^ (Invitrogen, Carlsbad, CA, USA) by digesting with BglII and XbaI, followed by ligation with a double-stranded DNA oligonucleotide (ds oligo) that was obtained by annealing the oligos YB007 and YB008 (see Supplementary Table S10, Additional File 23). After digestion of the resulting intermediate with ClaI and XbaI, a fragment containing the 3 × Flag-nls-cas9-nls coding sequence isolated from pBS-Hsp70-cas9 (Addgene, #46294) with same enzymes was inserted, yielding the final plasmid, which was verified by sequencing the insert region.

For chromosomal integration of the RMCE target region in SR9 cells by CRISPR/cas9, we generated pCFD3:U6:3_attP40, a derivative of pCFD3:U6:3gRNA (Addgene, #49410) for expression of a single guide RNA. The sgRNA was designed for targeting the “attP40” locus within a facultative *Msp300* intron on the left arm of chromosome 2. This region is known to host the recombinant transposon P{CaryP}attP40 that one of the most frequently used attP landing sites for PhiC31-mediated generation of transgenic *Drosophila* lines, including thousands of UASt-RNAi lines with limited basal and high GAL4-mediated expression [102]. The expression of flanking genes within a 40 kb range was found to be independent of temperature in S2R+ cells. pCFD3:U6:3gRNA was digested with BbsI and a ds oligo obtained by annealing YB109 and YB110 was inserted. The insert regions was verified by sequencing.

The plasmid pUC57-RMCE-target was generated for chromosomal integration of the target region required for recombinase-mediated directional cassette exchange. A synthetic EcoRI-HindIII DNA fragment inserted into the corresponding restriction sites of pUC57 was obtained from GenScript (Leiden, Netherlands). The insert DNA of this intermediate 1 contained the minimal attB target sites for the PhiC31 and Bxb1 integrases as well as the mEGFP coding sequence. An mRuby2 marker gene was generated and inserted between the attB target sites of intermediate 1 as follows. The mRuby2 coding sequence was amplified from pCDNA3_mRuby2 (Addgene, # 40260) using JB007 and JB008. The resulting PCR fragment was digested with EcoRI and XbaI, and inserted into the corresponding sites of pUASt downstream of the minimal promoter and the 5′ UTR of *Hsp70*. The resulting *Hsp70*P-mRuby2 cassette was then amplified from the pUASt-mRuby2 construct using JB005 and JB006. The PCR fragment was digested with NcoI and AvrII, and inserted into the corresponding sites of intermediate 1. The resulting intermediate 2 was complemented with a *copia* promoter-blas^r^ cassette after amplifying the cassette using JB011 and JB012 from a plasmid with a corresponding synthetic DNA insert obtained from GenScript. The resulting PCR fragment was digested with NcoI and SpeI, and inserted into the corresponding sites of intermediate 2, yielding intermediate 3. Finally, flanking homology arms for targeting to the attP40 region were added. The left homology region (HRl, 938 bp) was amplified from S2R+ genomic DNA using JB003 and JB004. The resulting PCR fragment was digested with BglII and AflII, and inserted into the corresponding sites of intermediate 3, yielding intermediate 4. The fragment with the right homology region (HRr, 573 bp), also amplified from S2R+ genomic DNA with JB009 and JB010, was digested with XhoI and HindIII, and inserted into the corresponding sites of intermediate 4 to arrive at the final construct pUC57-RMCE-target.

For the generation of the dual integrase expression plasmid pCo-Bxb1_PhiC31, we first deleted the blas^r^ coding region from a precursor plasmid (pCoBlast_mitoKillerRed) using a one-primer method [103] with primer JB020. The presence of the deletion was confirmed by sequencing. The region coding for PhiC31 was amplified from a template plasmid (pHsp70_phiC31-NLS_SV40; kindly provided by J. Bischof and K. Basler, University of Zurich, Zurich, Switzerland) using JB021 and JB022. The resulting PCR fragment was digested with EcoRI and XbaI, followed by ligation into the corresponding sites of the modified precursor plasmid, yielding pCo_PhiC31. The region coding for Bxb1 was amplified from the plasmid pET11_Bxb1 [104] using oEC134 and oEC135. The resulting PCR fragment was digested with AccIII and ligated into AgeI digested pCo_PhiC31, yielding the final construct pCo-Bxb1_PhiC31. In this product, a *copia* promoter fragment is upstream of the Bxb1 coding sequence. Moreover, the PhiC31 coding region is further upstream of the *copia* promoter fragment in reverse orientation with a minimal *Hsp70* promoter at its start. As a result, the two promoters (*copia* and *Hsp70*) drive bidirectional expression of the integrases, stimulated by transcription factors recruited by the *copia* promoter fragment. The correctness of the plasmid was confirmed by sequencing.

As a vector for the production of exchange plasmids to be used for RMCE in SR9rg cells, we designed pUC57-attP_P_-mcs-DSCP-nls-attP_B_. The insert region in this pUC57 derivative has the attP sequences for the PhiC31 and Bxb1 integrases on the left and right ends, respectively. Moreover, it contains a multiple cloning site (mcs) and the *Drosophila* synthetic core promoter (DSCP) [63] followed by a translational start codon and sequences coding for a nuclear localization signal (nls). The plasmid was obtained from GenScript and its insert region (EC358) is given in Supplementary Table S10, Additional File 23. Using a one-primer method [103] with YB317, a derivative of pUC57-attP_P_-mcs-DSCP-nls-attP_B_ was made lacking the DSCP. This pUC57-attP_P_-mcs-attP_B_ vector was used for assaying candidate CREs together with their linked endogenous promoter instead of relying on the DSCP.

Exchange plasmids with candidate CRE fragments for analysis after RMCE in SR9rg cells were generated using either pUC57-attP_P_-mcs-DSCP-nls-attP_B_ or pUC57-attP_P_-mcs-attP_B_. Candidate CREs were amplified by PCR from S2R+ cell genomic DNA (for primers, see Supplementary Table S10, Additional File 23). Alternatively, synthetic gene blocks (Supplementary Table S10, Additional File 23) were used. Some of the resulting exchange plasmids were further modified. To generate the exchange plasmids containing the fragments *pst*_E1b, *pst*_E1m, *pst*_E1n and *pst*_E1o, we introduced deletions into the exchange plasmid containing *pst*_E1 using a one-primer method [103] and either YB440, YB451, YB453, or YB439. This method and YB316 were also used to delete the DSCP from the exchange plasmid containing the *Lip4*_E fragment.

The plasmid pgEGFP-pst-blas^r^ was used for transfection of S2R+ cells and selection of random chromosomal integrations. It was obtained by modifying the precursor plasmid pCaSpeR4-gEGFP-pst, which was generated as follows. In a first step, the multiple cloning site of the pCaSpeR4 vector was modified to include an AvrII site. Thus, pCaSpeR4 was digested with BglII and XbaI, followed by ligation with a ds oligo obtained by annealing CL328 and CL329. Using the EcoRI and NotI in the resulting intermediate 1, we inserted a fragment encompassing the 5′ region of the *pst* locus after enzymatic amplification with CL330 and CL331 from *w*^1^ genomic DNA and digestions with EcoRI and NotI, yielding intermediate 2. A fragment encompassing the middle region of *pst* was amplified with CL332 and CL333 from *w*^1^ genomic DNA, digested with NotI and AvrII, and ligated into corresponding restriction sites of intermediate 2. The resulting intermediate 3 was modified by insertion of a fragment encompassing the 3′ region of *pst*, which was amplified with CL334 and CL335 from *w*^1^ genomic DNA. The PCR fragment as well as intermediate 3 were digested with AvrII and XbaI before ligation into intermediate 4. For insertion of the EGFP coding region, we used enzymatic amplification of this sequence with CL336 and SCH21, and inserted it into intermediate 4 using NotI to arrive at pCaspeR4-g-EGFP-pst. The *pst* gene region with an N-terminal EGFP insertion was then cut out from pCaspeR4-g-EGFP-pst using AfeI and XbaI. The resulting insert fragment was ligated with a vector fragment derived from pCoBlast. The vector fragment was made by digestion of pCoBlast with HindIII. The resulting overhangs were filled with Klenow fragment before digestion with a second restriction enzyme, XbaI. The ligation of insert and vector fragment yielded pg-EGFP-pst-blas^r^.

For the generation of *pst* mutant fly lines by CRIPSR/cas9, we generated derivatives of pCFD5:U6:3-t::gRNA (Addgene, #73914). A first derivative (pCFD5:U6:3-t::gRNA_pst-1) was made with the help of the gene block YB392 (Integrated DNA Technologies, Leuven, Belgium). The resulting plasmid allowed expression of two distinct gRNA targeting the *pst* coding region. An analogous construct (pCFD5:U6:3-t::gRNA_pst-2) for expression of another gRNA pair was made with gene block YB393. The gene blocks were digested with BbsI and ligated into the corresponding sites of pCFD5:U6:3-t::gRNA.

### Characterization of the clonal SR9rg cell line

The SR9rg cells, which we generated for analysis of temperature effects on CREs after RMCE, were derived from SR9 cells. Expression of *cas9* was induced in SR9 cells for 24 h by addition of CuSO_4_ (final concentration 500 μM) before co-transfection with the gRNA plasmid pCFD3:U6:3_attP40 and the repair template plasmids pUC57-RMCE-target. Blasticidin was added to the culture (final concentration 25 μg/ml) for selection of stable integration events. Microscopic analysis of the blasticidin-resistant cell population revealed mRuby expression as expected. By single cell cloning, we established 62 clonal lines. From seven of these, we were able to amplify a PCR fragment with a size indicating the presence of functional RMCE exchange target regions. Moreover, preliminary evidence from the initial PCR assays was consistent with on-target integrations in the “attP40” chromosomal region. However, these initial PCR analyses also indicated the presence of additional off-target integrations of sequences derived from pUC57-RMCE-target. One of the seven clonal cell lines, SR9rg, was chosen for evaluation of the efficiency of RMCE after co-transfection with the dual integrase expression plasmid pCo-Bxb1_PhiC31 and an exchange plasmid derived from pUC57-attP_P_-mcs-DSCP-nls-attP_B_. As these experiments indicated that SR9rg cells permit successful RMCE, we continued to use this cell line. In parallel, we analyzed the SR9rg cells in further detail, including whole genome sequencing (WGS) (see below). These analyses failed to confirm the presence of an on-target integration of the pUC57-RMCE-target sequences flanked by the left and right homology arms. Moreover, they confirmed the presence of off-target integrations of pUC57-RMCE-target sequences. Using the WGS data for read count analyses, sequences derived from pUC57-RMCE-target appear to be present in the SR9rg genome amounting to about 10 copies of the plasmid. By analysis of paired end reads, where one read maps to the *Drosophila* reference genome and the other to the pUC57-RMCE-target plasmid, sequences derived from this plasmid appear to be integrated in at least 34 distinct genomic locations. The analysis of such chimeric paired reads also suggested that several of these integrations have structures that cannot support an RMCE event, which results in a switch from mRuby to EGFP expression. The apparent structural complexity of the off-target integrations and the aneuploid genome of S2R+ cells precluded a comprehensive identification and structural clarification of all the integration events. We point out that we observed the same high propensity for undesired off-target integrations with S2R+ cells during several additional attempts to generate cell lines with single on target integration events by similar CRISPR/cas strategies. However, in SR9rg cells we also identified one clearly functional integration on chromosome 3 L that could be demonstrated to undergo the expected RMCE after cotransfection with the dual integrase expression plasmid pCo-Bxb1_PhiC31 and an exchange plasmid derived from pUC57-attP_P_-mcs-DSCP-nls-attP_B_ (Supplementary Fig. S14, Additional File 24). The FACS analysis of SR9rg derived cell populations obtained after RMCE with an exchange plasmid containing a strong enhancer suggested that the identified functional target region is not the only one, since we observed a fraction of cells expressing both red and green fluorescence beyond those expressing green but not red fluorescence (see for example Fig. 4B). The cells expressing both red and green fluorescence have presumably undergone RMCE at least at one but not at all functional target regions. The FACS analyses of SR9rg derived cell populations after RMCE with a strong enhancer also indicated that the number of functional target regions permitting the generation of a reporter gene expressing GFP by RMCE is either rather low, or that RMCE occurs usually with very high efficiency at almost all the functional regions. Otherwise, the population of cells expressing green but not red fluorescence after RMCE should have been exceeding low, in contrast to our observations (see for example Fig. 4B).

For confirmation of presence and function of the RMCE target region integrated within the *CG13288* locus in chr3L according to our WGS analysis (Supplementary Fig. S14, Additional File 24), we performed PCR assays using genomic DNA from SR9rg and SR9rg > *pst*_E1n cells and the primers oEC264, oEC267, oEC263, EC265 and YB654 (Supplementary Table S10, Additional File 23) .

### Expression profiling with DNA microarrays

For the analysis of the S2R+ cell transcriptomes at distinct temperatures (11, 14, 25 and 30 °C) (experiment M1), cells were seeded in 60 mm culture dishes in 2.5 ml medium in numbers adjusted to the temperature, to which they were shifted eventually (30 °C: 1.5 × 10^6^; 25 °C: 1.7 × 10^6^; 14 °C: 3.5 × 10^6^; 11 °C: 4.5 × 10^6^). One day after seeding, cells were shifted to different temperatures (11, 14, 25 or 30 °C) for 24 h before isolation of total RNA. Three biological replicates were performed (without prior computation of an appropriate sample size). RNA was isolated with the NucleoSpin RNAII kit (Macherey-Nagel, Oensingen, Switzerland).

For analysis of the effects of *His2Av* depletion on the temperature dependence of the S2R+ cell transcriptome (experiment M2), aliquots of cells were also seeded into 60 mm culture dishes in adjusted numbers (30 °C: 0.4 × 10^6^; 25 °C: 0.65 × 10^6^; 14 °C: 0.7 × 10^6^). Twenty-four hours after seeding, we added 30 μg dsRNA per dish, derived from either *His2Av* or *lacZ* for control. Four days after dsRNA addition, aliquots were shifted to different temperatures (14, 25 and 30 °C) for 24 h, followed by isolation of total RNA using the NucleoSpin RNAII kit. Three replicates were processed (without prior computation of an appropriate sample size).

For the time course analysis of the S2R+ cell transcriptome after a temperature downshift to 14 °C (experiment M3), we seeded aliquots of 1.4 × 10^6^ cells in 2.5 ml of medium in 60 mm culture dishes. After 36 h of incubation at 25 °C, culture aliquots were shifted to 14 °C. Total RNA was isolated at different times after the downshift (0, 4, 12, 24 and 72 h). In addition, instead of shifting to 14 °C, control aliquots were grown for an additional 12 h at 25 °C before isolation of total RNA. At least three biological replicates for each time point were generated (without prior computation of an appropriate sample size).

For analysis of a temperature downshift to 14 °C on the transcriptome of HB10 cells (experiment M4), aliquots of 2.6 × 10^6^ cells were seed into 35 mm culture dishes. After 36 h of incubation at 24 °C, half of the aliquots were shifted to 14 °C while the other half was maintained at 24 °C. Total RNA was isolated 24 h later. Three biological replicates were processed (without prior computation of an appropriate sample size). In case of the time course and HB10 cell experiments, total RNA was isolated using TRIzol (Invitrogen, cat# 15596026).

Before probe generation for DNA microarray hybridization, the total RNA preparations were further cleaned. Potential contaminations with DNA were eliminated by DNase I treatment, followed by RNA purification with the RNeasy MiniKit (Qiagen). The concentration of the resulting RNA preparations was determined with a NanoDrop 1000 Spectrophotometer V3.7 and RNA integrity was confirmed with an Agilent 2100 Bioanalyzer.

The work described in this publication was completed over several years and our initial transcriptome profiling started with DNA microarrays before RNA-Seq was customary. For generation of Cyanine 3 (Cy3)-labelled complementary RNA (cRNA) probes, we used the Low-Input QuickAmp kit (Agilent, Santa Clara, CA, USA). The resulting cRNA was purified using the Absolute RNA Nanoprep kit (Agilent). Successful Cy3 incorporation was confirmed by NanoDrop measurements and cRNA integrity with the Bioanalyzer. The hybridization mix (55 μl) contained 1.65 μg of fragmented cRNA, 2.2 μl of 25x hybridization buffer, 11 μl of 10x blocking agent and RNase-free water. For hybridization, we used single-color gene-expression microarrays purchased from Agilent. Two experiments (M1 and M2) were completed with *Drosophila* Gene Expression Microarray 4x44K (# G2519F, design ID: 018972) and two experiments (M3 and M4) with *Drosophila* Gene Expression Microarray (V2) 4x44k (#G2519F, design ID: 021791). After microarray hybridization and washing, signal intensities were recorded with Agilent Feature Extraction Software. Raw data obtained in experiments M1–4 have been deposited in NCBI’s Gene Expression Omnibus [105] and are accessible through GEO Series accession number GSE159174 (https://www.ncbi.nlm.nih.gov/geo/query/acc.cgi?acc=GSE159174).

In case of experiment M1, raw data was processed with GeneSpringGX11.0 (Agilent). Inter array differences were corrected using the option “median scaling to a control sample” of this software and the three replicates obtained after incubation at 25 °C as control samples. For comparison of the temperature dependence of signal intensities observed with a given microarray probe oligo with the signal intensities detected by other probes, a baseline transformation was applied so that all log_2_ values of signal intensities observed in the different replicates and temperature conditions with a given probe that a median = 0. Probes with low or incoherent values were filtered out before further analysis. Values were discarded, if the flag “present” was not specified in at least two of the three replicates obtained for a given temperature. Moreover, in case of the analyses concerning temperature-dependence of central cellular processes (Fig. 3A), probes with an expression level below 100 were not considered.

In case of the experiments M2–4, raw data was processed using R. For interarray comparisons, quantile normalization as implemented in the Bioconductor package preprocessCore [106] was applied in case of experiment M2. In case of the experiments M3 and M4, we used the limma package [107]. Signal intensities were background-corrected with normexp convolution method and quantile-normalized. Signal intensities were further adjusted with the R surrogate variable analysis (SVA) package [108] for removal of batch effects. We computed the median of signal intensities among technical replicate probes present more than once on the microarray. Moreover, for further analyses in case of experiment M3, we only retained probes that had log_2_ signal intensities higher than 6.6 on at least three of all the 23 analyzed microarrays. To identify probes with significant temporal change in signal intensities, we used an empirical Bayes extension of analysis of variance (ANOVA) as implemented in the limma package. Importantly, samples taken at t0 and at t12 after incubation at the control temperature 25 °C (ct12) were grouped and considered as reference baseline group for this statistical test, as the t0 and ct12 transcriptomes were very highly correlated (Pearson’s correlation coefficient *r* = 0.998 after comparison of t0 with t12_25 log_2_ average signal intensities of the three replicates). For further analysis, we selected all the probes with an FDR-corrected *p*-value < 0.05 and with a fold change in expression level ≥ 2 at one or more time points compared to the baseline value. To cluster probes with similar temporal expression profiles (Fig. 3E), we used computed Spearman distance metric among signal intensities and run a *k*-means cluster (R stats package). The elbow method [45] was used for assessing the number of clusters in our data set. Probes associated with systematic gene names were annotated with the R biomaRt library.

For the analyses concerning temperature-dependence of central cellular processes (Fig. 3A, F), we first generated curated lists of genes with unequivocal association to one of the selected functional networks or to an organelle (S phase, M phase, autophagy, proteasome, amino acid catabolism, insulin/TOR signaling, cytoplasmic ribosome, mitochondrial ribosomes, oxidative phosphorylation, glycolysis, TCA cycle, beta oxidation, lipid metabolism, and peroxisome). To generate these gene lists (Supplementary Table S11, Additional File 25), *D. melanogaster* genes were first filtered using corresponding GO terms. The resulting list were manually curated and further complemented using information from the KEGG database, as well as from various publications, including [109]. All probes annotated to the selected genes were identified and probes with significant expression were used for quantitative analyses. The resulting heat map (Fig. 3A) displays log_2_-transformed signal intensities of average fold change normalized to the means. The heat map displaying the time course analysis (Fig. 3E) displays log_2_ values of average fold change at the indicated time points relative to expression at t = 0. All the additional analysis of functional associations of differentially expressed genes (Fig. 3B) were performed using the option “Proteins with Values/Ranks” of String (v11.0) [110].

For the analysis of temperature effects on genes known to be regulated during response to different types of cellular stress, we first generated lists of stress-regulated genes primarily based on the results of [58] and additional publications cited by these authors [59–61]. These publications describe microarray analyses of the transcriptome response in adult flies or larvae after exposure to various stressors (oxidative stress: paraquat, hydrogen peroxide, hyperoxia; ER stress: tunicamycin; starvation; bacterial or fungal infection). For each type of stress, genes induced more than 1.5 fold (or 2 fold in case of paraquat) were selected for our analysis. All probes detecting transcripts derived from these stress genes were identified (Supplementary Table S11, Additional File 25) and the signals observed with these probes in our microarray experiments were extracted for the analysis of temperature on stress gene expression in S2R+ cells.

Additional information concerning presentation of the microarray data in various figures is described further below.

### Expression profiling with 3′ RNA-Seq

S2R+ cells were plated into 60 mm dishes. The number of plated cells was adjusted according to the temperature to be analyzed eventually (30 °C: 1.5 × 10^6^ cells; 25 °C: 1.7 × 10^6^ cells; 14 °C: 3.5 × 10^6^ cells; 11 °C: 4.5 × 10^6^ cells). Three biological replicates were prepared for each temperature (without prior computation of an appropriate sample size). All aliquots were first cultured at 25 °C for 24 h after plating, before shifting to either 11, 14, 25 or 30 °C.

For the analyses with adult males, *w*^1^ flies were used to start cultures in bottles at 25 °C. Newly eclosed adult male flies (0–12 h after eclosion) were collected and distributed into aliquots into fresh food vials. Three biological replicates were prepared for each temperature. The aliquots were then shifted to either 11, 14, 25 or 30 °C.

After 24 h of incubation at the different target temperatures, total RNA was extracted from the S2R+ cells and the male flies using TRIzol (Invitrogen, cat# 15596026), followed by DNase digestion (DNA-free DNA Removal Kit, Ambion, cat# AM1906). Libraries were generated with the QuantSeq 3′ mRNA-Seq Kit REV for Illumina (Lexogen) and sequenced by the Functional Genomics Center Zürich (FGCZ, University and ETH Zürich). 2 × 150 bp paired-end sequencing was performed with HiSeq4000 (llumina, San Diego, CA).

Data analysis was completed as follows. To identify genes differentially expressed at the different incubation temperatures, sequencing data from the 24 samples (12 samples from S2R+ cells and 12 samples from adult males, 419.5 million reads in total) were aligned to the *Drosophila melanogaster* (dm6) reference genome (Ensembl version 98) with STAR aligner [111]. The STAR dm6 index was built with adjusted parameters (−-genomeSAindexNbases 10) to accommodate the size of the genome. Moreover, the gene annotation was incorporated to allow correct mapping of spliced reads. After alignment, count tables of reads mapping to genes across all the 24 samples were obtained using htseq-count (Anders et al. 2015). Normalization was computed using the cpm function of edgeR [112]. Two comparisons (Cells 11 °C + 14 °C vs. Cells 25 °C + 30 °C and Flies 11 °C + 14 °C vs. Flies 25 °C + 30 °C) were made using edgeR. Weakly expressed genes were filtered out using HTSFilter [113].

To calculate the correlation between the results of 3′ RNA-Seq and DNA microarray analyses, log_2_ values of fold change of the expression level of a given gene at high temperature (25 and 30 °C values pooled) compared to low temperature (11 and 14 °C values pooled) was evaluated using Pearson’s method.

For analysis of alternative polyadenylation, a poly(A) site (PAS) database was constructed by processing the pooled 3′ RNA-Seq reads from all samples in a first step. After read-mapping to the reference genome (dm6), the position of the last nucleotide before the start of the poly(A) tail in given read was determined for each read. This position represents the cleavage and polyadenylation site. The detected PAS positions were assigned to clusters using a window size from 25 nucleotides upstream to 25 nucleotides downstream of the actual PAS. This clustering accounts for the fact that cleavage and polyadenylation do not occur with single nucleotide precision. Since the REV version of the Lexogen QuantSeq 3′ mRNA-Seq Kit was used, we filtered out positions in the PAS database that corresponded to internal priming (IP) events rather than true polyadenylation. To this end, the genomic sequence in the region from − 30 to 10 around each putative PAS was checked and all sites which contained seven or more consecutive A nucleotides in this region were removed from the PAS database. Moreover, putative PAS were also removed if the region from − 30 to 0 did not contain any of the known polyadenylation signals [114]. The filtered PAS in the database were assigned to genes. This was accomplished after extending the 3′ UTR region annotated in FlyBase to include an additional 5000 nucleotides or up to the middle of the distance between the PAS and the start of the downstream gene. This extension was motivated by the observation that 3′ UTR annotations are overly stringent. The database with all the filtered and assigned PASs was used for the generation of count tables. For each condition (cells or flies at either 11, 14, 25 or 30 °C, respectively) the read counts observed at the PASs in the database were determined. Normalized values were computed using the cpm function of edgeR. To identify genes with APA switches, we used DESeq [115] for the analysis of two comparisons (Cells 11 °C + 14 °C vs. Cells 25 °C + 30 °C, Flies 11 °C + 14 °C vs. Flies 25 °C + 30 °C). For each gene, we considered all possible poly(A) site pairs and selected only those pairs that have opposing direction (fold-change) of regulation in a particular comparison with FDR < 0.05. If only one PAS had an adjusted *p* value < 0.05, then the second PAS was selected based on highest read count. If none of the PAS had a p value < 0.05, then both PASs were selected based on highest read count. Among the retained pairs, the one with the greatest fold-change distance was ultimately chosen and labeled as enhanced (fold-change (proximal/distal) > 0) or repressed (fold-change (proximal/distal) < 0). Thereby we obtained lists of genes with temperature-dependent differential APA, which contained for each of these genes the two PASs that were most divergently used in a particular comparison.

The 3′ RNA-Seq data have been deposited in NCBI’s Gene Expression Omnibus [105] and are accessible through GEO Series accession number GSE159174 (https://www.ncbi.nlm.nih.gov/geo/query/acc.cgi?acc=GSE159174) and also at http://www.expressrna.org/index.html?action=library&library_id=20170831_yu.

For generation of correlation plots (Figs. 2A, E, 3D, Supplementary Fig. S7) and volcano plots (Fig. 2B, E; Supplementary Fig. S7), data for expression levels after normalization and filtering to eliminate genes with spurious expression was processed with the R packages corrplot [116] and EnhancedVolcano [117]. In the line diagram illustrating temperature effects on *Hsp* transcript levels (Fig. 3C), log_2_ values of fold changes (average of three biological replicates and multiple probes, if present) relative to expression at 14 °C were displayed, as revealed by the microarray analysis, where some probes detected transcript derived from multiple *Hsp* genes. In the four box plots (Fig. 3e), normalized log_2_-transformed signal intensities detected in all the replicates are displayed. For normalization, the mean log_2_ signal intensity over all the samples obtained for a given probe was subtracted from the log_2_ signal intensity detected in the samples.

### ATAC-Seq

Aliquots of 3.5 million S2R+ cells were seeded in round 60 mm dishes in case of the samples that were eventually exposed to 25 or 29 °C. In case of the samples eventually exposed to 14 °C, the starting number of cells was 7 millions. Four replicate cultures were prepared for each analyzed temperature (without prior computation of an appropriate sample size). After a 36 h incubation at 25 °C, the four aliquots were shifted to either 14, 25 or 29 °C. Cells were harvested 24 h after this temperature shift. One aliquot for each temperature was harvested first at room temperature for cell count determination. Cell harvest in case of all the remaining aliquots was performed quickly using 0.025% Trypsin-EDTA (ThermoFisher Scientific, 25,200–056) within the incubators and at the temperatures used during the preceding 24 h incubation, with solutions pre-equilibrated to the different incubation temperatures. The culture medium was removed and cells were rinsed once with PBS before addition of 1 ml Trypsin-EDTA solution for 5 min. The released cells were transferred into a 15 ml Falcon tube and combined with four rinses of the plates (each with 1 ml of complete medium). The resulting master cell suspension was diluted into ice-cold complete medium in order to obtain an Eppendorf tube with 75,000 cells in 1 ml for each aliquot. Cells were sedimented (500×g for 5 min, 4 °C) and washed once with 50 μl PBS. Cells were resuspended and lysed with 50 μl of cold lysis buffer (10 mM Tris-HCl, 10 mM NaCl, 3 mM MgCl_2_, 0.1% NP-40). Lysed cells were sedimented (500×g for 10 min, 4 °C). Tagmentation was performed during 30 min at 25 °C in a 50 μl reaction in TD Buffer (Illumina, FC-121-1030) containing 2.5 μl Tn5 Transposes (Illumina, FC-121-1030) [32]. Tagmented DNA was purified using a Qiagen MinElute Kit and amplified as described [32] using a Nextera forward primer (Ad1_noMX; Supplementary Table S10, Additional File 23) in combination with a replicate-specific bar-coded reverse primer (Ad2.1 to Ad2.9; Supplementary Table S10, Additional File 23). Libraries were purified using Qiagen PCR Cleanup Kit. Samples were pooled and sequenced at the FGCZ using HiSeq2500 v4 providing 126 bp paired end reads. The nine ATAC-Seq libraries were sequenced to a median depth of 52 million reads per sample.

The sequence data was further processed as follows. Sequencing adaptors were trimmed with Trimmomatic (version 0.38) [118]. All reads were mapped to *Drosophila* genome dm6 using Bowtie2 [119] with the parameters -X 2000 --very-sensitive. Non-mapped, non-unique, and mitochondrial reads were filtered out with SAMtools [120, 121]. PCR duplicates were marked and removed with Picard (https://broadinstitute.github.io/picard/). Subsequently, all reads were adjusted for the binding footprint of Tn5 transposase, i.e., all reads aligning to the top strand were shifted by four bp and all reads aligning to bottom strand were shifted five bp in 5′ direction.

ATAC-Seq peaks were called with MACS2 [122] for each library with parameters -f BEDPE --keep-dup all -q 0.01 -g dm --nomodel. Differential peaks were analyzed with DiffBind [123]. Read counts were obtained with the dba.count function. To keep the peaks at a consistent width, peaks were re-centered around the point of greatest enrichment with “summits = 250”, yielding 17,175 consensus peaks among all nine libraries. Next, DESeq2 [124] was employed for count normalization and differential analysis using default threshold of FDR < = 0.05. Normalized peaks with low reproducibility among replicates were eliminated as described [125]. The ATAC-Seq data have been deposited in NCBI’s Gene Expression Omnibus [105] and are accessible through GEO Series accession number GSE159174 (https://www.ncbi.nlm.nih.gov/geo/query/acc.cgi?acc=GSE159174).

### Whole genome sequencing

For the characterization of SR9 derived cell lines by whole genome sequencing, we selected seven lines, including SR9rg, obtained by single cell cloning from the cell population resulting after SR9 cell transfection with pCFD3:U6:3_attP40 and pUC57-RMCE-target. In addition, we selected three additional clonal cell lines that were obtained after transfection of SR9 cells with pCFD3:U6:3_attP40 and distinct repair template plasmids. The selected cell lines were cultured in 25-cm^2^ flasks at 25 °C. DNA was isolated using QIAamp DNA Mini Kit (Qiagen, 51,306). Libraries were prepared with TruSeq Nano DNA library Prep kit and sequenced at the FGCZ. 2 × 150 bp paired-end sequencing was performed with HiSeq4000.

Preqc [126] was used to estimate sequence coverage, per-base error rates, as well as genome size, heterozygosity and repeat content. De novo assembly of the paired-end sequencing data was performed using SPAdes [127] with default parameters. Additionally, AByss [128] was also used for de novo assembly with SPAdes error corrected reads. QUAST [129] analysis showed that these two algorithms produced similar results in terms of the number and length of contigs and scaffolds identified in each sample. Assembly quality of SPAdes and Abyss was determined using QUAST.

We built three in-silico reference genomes, which consisted of the *Drosophila* reference genome sequence (dmel_r6.19) plus the plasmid sequence of pMT-cas9-hygro^r^ and of one of the three distinct repair plasmids (including pUC57-RMCE-target). Paired-end reads were aligned to in-silico reference genome using BWA aligner [130], duplicates were marked by SAMBLASTER [131] and removed. The in-silico genomes were then used as reference genomes for a search of hybrid structure variants (hSvs) linking dmel_r6.19 sequences with sequences in the plasmids. To do so, we used two algorithms LUMPY [132] and Delly [133]. In the case of LUMPY analyses, reads with mapping quality either above Q20 or Q30 were used. In the case of Delly analyses, either all reads or reads with mapping quality above Q30 were used. Paired read analysis for the identification and mapping of the chimeric pairs, where one read was aligned to the *Drosophila* reference genome and the other read to the plasmid sequences, was performed by homemade scripts written in Python. Copy number variation was analyzed by CNVnator [134].

### Generation of *pst* mutant fly lines

CRISPR/cas9 was used for the generation of fly lines with mutations in *pst*. The plasmids allowing expression of a pair of gRNAs (see above) were injected into eggs collected from *yw*; *attP40*{*nos-cas9}/*CyO flies, respectively (BestGene Inc., Chino Hills, CA, USA). The males that eclosed from the injected eggs were crossed individually with virgins w*; *Sb*/TM3, *Ser*. Some of the F1 progeny resulting from these crosses were analyzed with a PCR assay, involving amplification from genomic DNA with the primers YB444 and YB445 (after injection of gRNA plasmid 1) or YB446 and YB447 (after injection of gRNA plasmid 2), which anneal to sites flanking the gRNA target sites. Therefore, shortened PCR products indicated deletion of the *pst* sequences between the two gRNA target sites. If deletion fragments were detected among the tested progeny of a given founder male, additional male progeny flies derived from this founder male were crossed individually to virgins w*; *Sb*/TM3, *Ser*. Some progeny from these crosses were again analyzed with the PCR assays described above for the identification of flies with intragenic *pst* deletions. Two independent lines were established: w*; *pst*^cc1^/TM3, *Ser* and w*; *pst*^cc2^/TM3, *Ser*. DNA sequencing of the PCR fragment spanning the intragenic deletions revealed the presence of the following mutations. The *pst*^cc1^ allele is caused by an intragenic out-of-frame deletion. It is predicted to express a protein that includes only the first 94 amino acids of Pst. The *pst*^cc2^ allele is caused by an intragenic in-frame deletion. The Pst-PE variant protein expressed from this allele is predicted to lack a central region of 309 amino acids (...VVGSSGTSSVSSNCCN-deletion-GKGFLLNGDV...).

## Supplementary Information

**Additional file 1: Table S1**. Excel file with data from DNA microarray analysis of temperature dependence of the S2R+ cell transcriptome (M1).

**Additional file 2: Table S2**. Excel file with log2 values of the fold changes in transcript levels observed when comparing low and high temperatures by DNA microarray analysis with S2R+ cells (worksheet 1), 3′ RNA-Seq with S2R+ cells (worksheet 2), 3′ RNA-Seq with adult male flies (worksheet 3) and DNA microarray analysis with HB10 cells (worksheet 4). Expression at low temperature represents the mean observed at 11 and 14 °C, while expression at high temperature the mean at 25 and 30 °C, except for the analyses with HB10 cells where expression at 14 and 24 °C is compared.

**Additional file 3: Fig. S1**. Concordance of transcript levels after quantification with different methods. (A-C) Temperature effects on transcript levels in S2R+ cells were analyzed using either qRT-PCR (A), microarrays (B) or 3′ RNA-Seq (C). The bar diagram displays relative expression levels at the different temperatures, as detected by the different methods. Expression at 25 °C was set to 1. For each analysis, culture aliquots were shifted for 24 h to the indicated temperatures (11, 25 and 30 °C) before RNA isolation. Representative CoolUp genes (*CG13694*, *CG6321*, *CG32944, Orct2*, *Lip4*) and CoolDown genes (*Hsp23*, *Hsp22*, *DNApol-α50*), as well as genes (*Atg1*, *Pten*), which were barely temperature regulated, were selected for validation by qRT-PCR. In case of qRT-PCR, mean and s.d. of three technical replicates are displayed. In case of the microarray data, mean and s.d. of three biological replicates and multiple probes, if present, are shown. In case of the 3′ RNA-Seq data, mean and s.d. of three biological replicates are presented.

**Additional file 4: Fig. S2**. Increased use of distal polyadenylation sites at low temperature. (A-C) Microarray probes with pronounced CoolUp signals were observed to detect specifically the annotated transcripts with the longest 3′ untranslated region, as illustrated in case of the gene (A) *RpLP1*, (B) *Kap-α3* and (C) *ewg*. The positions recognized by different probes are indicated (vertical bars of light grey shading) in a scheme with the transcribed region and the annotated transcripts. The bar diagrams below display signal intensities (mean of three biological replicates) observed with these probes after incubation of S2R+ cells at different temperatures (11, 14, 25 and 30 °C).

**Additional file 5: Table S3**. Excel file with data from the analysis of temperature dependence of the S2R+ cell transcriptome by 3′ RNA-Seq.

**Additional file 6: Table S4**. Excel file with data from the analysis of temperature dependence of the transcriptome of adult male flies by 3′ RNA-Seq.

**Additional file 7: Table S5**. Excel file with data from DNA microarray analysis of temperature dependence of the HB10 cell transcriptome (M4).

**Additional file 8: Table S6**. Excel file with 3′ RNA-Seq count data from the analysis of temperature effects on the use of alternative polyadenylation sites in S2R+ cells (worksheet 1) and adult male flies (worksheet 2).

**Additional file 9: Fig. S3**. Temperature-regulated alternative polyadenylation. (A-D) 3′ RNA-Seq data obtained from S2R+ cells (A, C) and adult male flies (B, D) after 24 h of incubation at different temperatures (11, 14, 25 and 30 °C) was used for an analysis of temperature effects on the choice of polyadenylation sites (PASs). (A,B) Histograms illustrate the frequency of alternative polyadenylation (APA) in S2R+ cells (A) and adult male flies (B), as detected after pooling all data obtained at the different temperatures from the three biological replicates. In total, 14,669 PASs were detected in S2R+ cells, and 22,378 in adult male flies. These were assigned (see Materials and Methods) to a total of 7495 and 10,757 expressed genes in S2R+ cells and adult male flies, respectively. (C,D) Temperature effects on APA. For genes with multiple PASs, we compared the two PASs with highest presence (read counts) across all conditions and required the log_2_ fold change to be in opposite directions when comparing the read counts at the high (25 and 30 °C) with those at the low (11 and 14 °C) temperatures. The scatter plots display the fold changes at the proximal and distal of these two most strongly affected PASs in S2R+ cells (C) and adult flies (D). Blue dots represent genes (class I), where preference of the distal over the proximal PAS at low temperature is significant in comparison to the high temperature. Red dots represent genes (class II), where temperature change has a significant effect in opposite direction.

**Additional file 10: Fig. S4**. Downregulation of cell cycle genes in S2R+ cells at low temperature. (A) Temperature dependence of transcript levels of S phase genes. The S2R+ cell transcriptome was analyzed after 24 h of incubation at different temperatures (11, 14, 25 and 30 °C) using microarrays. Temperature dependence of signal intensities (mean of three biological replicates) observed with microarray probes for transcripts derived from a curated set of genes crucial for progression through S phase is displayed after baseline transformation. (B) Temporal dynamics S- and M phase gene downregulation after a shift from 25 to 14 °C. Transcriptome changes were analyzed with microarrays at different times (0, 4, 12, 24 and 72 h) after the temperature downshift. Fold change relative to expression at t = 0 (dashed green line) of signal intensities (mean of three biological replicates) observed with microarray probes for transcripts derived from a curated set of genes crucial for progression through S- and M phase was calculated. Mean and s.d. are displayed in red. (C) Clustering of temperature-regulated genes according to their temporal expression dynamics after a 25- > 14 °C shift using *k*-means identified a prominent cluster of genes with persistent downregulation (Fig. 3E, cluster 2). Functional interactions among the genes in this cluster, as revealed by analysis with the STRING database, are displayed. Genes associated with the GO term “DNA replication” (GO:0006260), the term most strongly enriched (FDR = 4.15 × 10^− 26^) by the genes in this cluster, are marked in blue. Genes associated with “cell cycle” (GO:0007049, also strongly enriched, FDR = 3.72 × 10^− 15^) are marked in red.

**Additional file 11: Table S7**. Excel file with data from DNA microarray analysis of the temporal dynamics of transcriptome changes in S2R+ cells after a shift to 14 °C (M3).

**Additional file 12: Fig. S5**. Absence of stress response gene induction after temperature downshift to 14 °C. Microarray data from the time course analysis of S2R+ cell transcriptomes after a 25- > 14 °C temperature shift were used for an analysis of the response of known stress response genes. Probes detecting transcripts of genes previously reported to be induced by the indicated stressors were identified. Signals obtained at t0 were set to 1 and fold change at different times after temperature downshift was calculated. Swarm plots display log_2_ values of the fold changes, as well as means and s.d. in red.

**Additional file 13: Fig. S6**. Transient activation of JNK and p38 protein kinases after temperature downshift to 14 °C. (A,B) Immunoblotting with antibodies specific for the activated forms of JNK (phospho-JNK) and p38 (phospho-p38) were used for analysis. S2R+ cells were plated and grown at 25 °C before a shift to 14 °C at t = 0. Extracts were prepared at t = 0, 4, 12, 24, and 72 h after downshift, and also from an aliquot maintained for 12 h at 25 °C after t = 0. Relative amounts of extracts analyzed by immunoblotting are indicated. Arrows mark the bands corresponding to phospho-JNK, JNK and phospho-p38, and dashes the position of molecular weight markers. (A) For control of antibody specificities, we also analyzed extracts from S2R+ cells treated with lipopolysaccharide (LPS) or depleted of *bsk* transcripts (coding for JNK) or GFP transcripts (for control) by RNAi. Immunoblots were probed with anti-phospho-JNK, anti-JNK and anti-α-tubulin. Signal intensities in the bands representing JNK and phospho-JNK were quantified in four replicates. A bar diagram displays average intensities (+/− s.d.) normalized to those at t = 0. (B) For comparison, we also analyzed extracts from S2R+ cells after exposure (45 min) to a heat shock at 37 °C. Relative amounts of extracts analyzed by immunoblotting are indicated. Immunoblots were probed with anti-phospho-p38 and anti-α-tubulin. The arrow indicates the band corresponding to phospho-p38. Position of molecular weight markers are indicated. Signal intensities in the bands representing phospho-p38 were quantified in three replicates. A bar diagram displays average intensities (+/− s.d.) normalized to those at t = 0.

**Additional file 14: Fig. S7**. Evaluation of the role of histone H2Av (His2Av) in transcriptional control of temperature-regulated genes in S2R+ cells. (A) To address the role of His2Av in temperature-dependent regulation of gene expression in S2R+ cells, we added *His2Av* dsRNA or *lacZ* dsRNA for control to culture aliquots followed by a shift to the indicated temperatures after four days. Twenty-four hours after the shift, we isolated total RNA for analysis with microarrays. Three replicate experiments were performed. (B) Analysis of transcript levels of *His2Av* and *Act5C* (for control) confirmed successful depletion of *His2Av* transcripts. Bar diagrams display transcript levels (average of three biological replicates and s.d.) at the analyzed temperatures. Average expression at the three temperatures observed after treatment with *lacZ* dsRNA was set to 100%. (C) Analysis of His2Av depletion by immunoblotting. S2R + _*MtnA*p-*His2Av*-mRFP cells (Lidsky et al. 2013) were treated with dsRNA derived from *lacZ* (for control) and two distinct *His2Av* amplicons (*H2Av*_1 and *H2Av*_2) for three and 5 days as indicated. Cell extracts were analyzed by immunoblotting with anti-mCherry (detecting His2Av-mRFP) and anti-PSTAIR (loading control). The combined treatment with both *His2Av* dsRNA preparations (*H2Av*_1 + 2) for 5 days was found to reduce His2Av-mRFP levels to 40% of controls according to quantification of signal intensities and normalization based on anti-PSTAIR signals. (D) Effects of *His2Av* depletion on the S2R+ cell transcriptome. A volcano plot is displayed with probes associated with signal intensities that were significantly (FDR < 0.05; fold change ≥2) down- (green dots, 320 probes) or upregulated (brown dots, 300 probes) by *His2Av* depletion in comparison to control (*lacZ* dsRNA treatment). The comparison based on signals detected at 14 °C is shown. Analogous comparisons at 25 or 30 °C resulted in comparable observations. (E) Effects of *His2Av* depletion on transcript levels of temperature-regulated genes. Probes associated with a fold change in signal intensity ≥2 in the comparison of 14 and 30 °C in both control experiments (with *lacZ* dsRNA treatment and without any dsRNA treatment) were selected for further analysis. Plots summarize the signal intensities obtained with these probes, separated into CoolUp and CoolDown probes, after treatment with either *lacZ* or *His2Av* dsRNA at the three different temperatures. The comparison revealed at most subtle effects of *His2Av* depletion on transcript levels of temperature-regulated genes in S2R+ cells, in contrast to results from *Arabidopsis thaliana* where a reduction in the homolog His2A.Z has been found to result in a dramatic conversion of the expression of genes that are normally temperature-regulated into a constitutive warm-like expression [20]. (F) Fold change correlation analyses for further analysis of the effect of *His2Av* depletion on temperature-dependent gene regulation. Scatter plots of log_2_ fold changes for all probes with significant signals are displayed. *r* = Pearson’s correlation coefficient. As revealed by the left panel, the fold changes observed when comparing expression levels at 14 and 30 °C were very strongly correlated in the two control experiments (with *lacZ* dsRNA treatment and without any dsRNA treatment). However, there was essentially no correlation between the fold changes resulting from temperature change and those resulting from His2Av depletion at 14 °C (middle panel) or 30 °C (right panel), in contrast to results from similar analyses in *Arabidopsis thaliana* [20], where reduced loading of the homolog H2A.Z into chromatin (in *arp6–10* mutants) results in *r* = 0.68 for the correlation between transcriptome regulation by temperature and misregulation by reduced H2A.Z.

**Additional file 15: Table S8**. Excel file with data from DNA microarray analysis of the effects of RNAi with *His2Av* dsRNA or *lacZ* dsRNA (for control) on temperature dependence of the S2R+ cell transcriptome (M2).

**Additional file 16: Table S9**. Excel file with data from the analysis of temperature dependence of DNA accessibility in S2R+ cells by ATAC-Seq, with consensus peaks (worksheet 1) and DE peaks 14 vs 29 °C (worksheet 2).

**Additional file 17: Fig. S8**. DNA accessibility in nuclear chromatin of S2R+ cells. For illustration of ATAC-Seq data quality, various analyses based on one of the samples (replicate of cells tagmented after growth at 25 °C) are displayed. (A) Distribution of ATAC-Seq fragment lengths. Arrows indicate periodicity reflecting nucleosomal organization. (B) Enrichment of ATAC-Seq reads around annotated transcription start sites (TSS). Distance (base pairs [bp]) from the transcriptional start site (TSS) is indicated on the x axis. (C) Distribution of ATAC-Seq reads across annotated genome features.

**Additional file 18: Fig. S9**. Assay for temperature-dependent enhancer activity with candidate CREs. (A) Table summarizing location and activity of candidate CREs analyzed after RMCE with SR9rg cells. (B) Data for the analyzed candidate CREs. Candidate CREs (designation in bold on the left side) were selected based on data obtained by ATAC-Seq (top three browser tracks) and 3′ RNA-Seq (bottom three browser tracks). Grey shading represents the region of the analyzed DNA fragment. Green arrowheads indicate the direction of transcription of the proximal gene. Scatter plots on the right side present green (x axis) and red (y axis) fluorescence intensities as determined by flow cytometry with SR9rg cells after RMCE and incubation at the indicated temperatures.

**Additional file 19: Fig. S10**. Dissection of *pst*_E1 region. (A) Schematic illustration of analyzed truncation series, with transcription start sites (kinked arrows), untranslated regions (grey boxes), coding region (yellow boxes) and introns (white boxes) indicated. (B) Scatter plots of the results obtained by flow cytometry after RMCE with SR9rg cells and incubation at the indicated temperatures.

**Additional file 20: Fig. S11**. Temperature dependence of transcript levels of candidate regulators of *pst*_E1n enhancer activity and dependence on Pvf/Pvr. (A-C) Transcript levels at the indicated temperatures in S2R+ cells as detected by 3′ RNA-Seq. Bar diagrams display log_2_ of the average of the read counts (after normalization) and s.d. (*n* = 3 replicate experiments). (A) Transcript levels of TFs with predicted binding sites within *pst*_E1n and detectable expression in S2R+ cells. (B) Transcript levels of JAK/STAT signal transduction proteins. Expression of *upd1* is marginal at most in S2R+ cells and hence not included. (C) Transcript levels of the genes encoding the receptor tyrosine kinase Pvr and its known ligands Pvf2 and Pvf3. Expression of *Pvf1* is marginal at most in S2R+ cells and hence not included. (D) Dependence of *pst*_E1n enhancer activity on Pvfs and Pvr. The indicated dsRNAs were used for depletion in SR9rg > *pst*_E1n (mRuby^−^, GFP^+^) cells, which were then shifted in aliquots to the indicated temperatures and analyzed eventually by flow cytometry. Bars display median GFP signal intensity. For depletion of Pvr and Pvfs, two dsRNA preparations (1 or 2) generated from distinct amplicons, were used in multiple experiments (indicated by a, b or c) in some cases.

**Additional file 21: Fig. S12**. Enhancer activity after depletion of TFs, JAK/STAT and Pvr. (A,B) The indicated dsRNAs were used for depletion of TFs with predicted binding sites in *pst*_E1n. In addition, JAK/STAT signaling pathway proteins (Dome and Socs36E) and the receptor tyrosine kinase Pvr were depleted. In some cases, two dsRNA preparations (1 or 2) generated from distinct amplicons were used. *lacZ* ds RNA was used in two replicates (a and b). Depletions were performed in SR9rg > *pst*_E1n (mRuby^−^, GFP^+^) cells (A) and in SR9rg > *ced-6*_E (mRuby^−^, GFP^+^) cells (B). Depleted cells and control cell lines (S2R+ and SR9rg) were then shifted in aliquots to the indicated temperatures and analyzed eventually by flow cytometry. Bars display median GFP signal intensity.

**Additional file 22: Fig. S13**. Depletion efficiency and effect on endogenous *pst* transcript levels. (A,B) The indicated dsRNAs were used for RNAi with SR9rg > *pst*_E1n (mRuby^−^, GFP^+^) cells. Two dsRNA preparations (1 or 2) generated from distinct amplicons were used for some targets. The number of independent experiments is indicated in brackets. In addition, *lacZ* dsRNA was used in parallel for control. Bars represent average transcript levels as determined by qRT-PCR relative to those detected after *lacZ* depletion, which were set to 1. Whiskers indicate s.d. in case of multiple independent experiments. (A) To assess RNAi efficiency, total RNA was isolated after four days of depletion at 25 °C followed by analysis of the target transcript levels. The target analyzed in case of double depletion is indicated at the bottom. (B) The levels of transcripts derived from the endogenous *pst* gene were analyzed.

**Additional file 23: Table S10**. Excel file with description of synthetic DNA fragments (oligos and gene blocks).

**Additional file 24: Fig. S14**. Characterization of SR9 and SR9rg cells. (A-D) Identification of a functional target locus for RMCE in SR9rg cells. Whole genome sequencing was used for the characterization of the clonal SR9rg cell line. Hybrid paired reads, where one read aligns to genomic sequences and the mate read to pUC57-RMCE-target plasmid sequences were identified and used for mapping chromosomal insertions of pUC57-RMCE-target. An insertion that is competent for RMCE was identified on chromosome 3 L in an intron of the *CG13288* gene. (A) The genomic region (dmel_r6.19, chr3L:5904000 to chr3L:5921000) with *CG13288* as visualized by GBrowse 2.0 is displayed on top. The cumulative base coverage by hybrid paired reads mapping to this region is indicated by the blue bars below (from 0 to 55 reads/bp). Black arrows emphasize the distal clustering of the abrupt end of the alignments on either side, corresponding to the genome-plasmid junctions. Primers “L” and “R” (red arrows) with sequences close to the left and right ends of this genomic region were used for confirmation of the insertion by PCR (see panel D). (B) A linearized representation of pUC57-RMCE-target is shown on top. The grey bars below display the cumulative base coverage (from 0 to 51 reads/bp) by the hybrid paired reads mapping to the *CG132888* region. Black arrows and dashed lines indicate breakpoints (abrupt clustered ends of read alignments). Primers “r” and “l” (red arrows) with sequences close to the right and left ends of the plasmid region were used for confirmation of the insertion by PCR (see panel D). (C) Proposed organization of pUC57-RMCE-target insertion in the *CG13288* region. The plasmid pUC57-RMCE-target is inserted almost completely, starting from breakpoint “l” and extending to the breakpoint “r” (see panel B). The inserted plasmid region is flanked by a tandem duplication of the genome region between “L” and “R” (see panel A). Primers used for confirmation of the proposed organization are indicated (red arrows). (D) PCR assay for confirmation of the proposed organization of the pUC57-RMCE-target insertion in the *CG13288* region and of its competence for RMCE. Genomic DNA isolated from the indicated cells was analyzed by PCR with the primer pairs indicated in the red boxes. Primer P in the last pair anneals to the *pst*_E1n region, which is only present after RMCE, i.e., only in the SR9rg > *pst*_E1n cells. L = oEC264, R = oEC267, l = oEC263, r = oEC265, P = YB654. (E) Characterization of SR9 cells. These cells were obtained after transfection of S2R+ cells with the plasmid pMT-cas9-hygro^r^ schematically shown on top. For confirmation of CuSO_4_-inducible expression of *cas9* in the SR9 cells, they were cultured in parallel with the parental S2R+ cells for 24 h without or with 500 μM CuSO_4_ as indicated. Total extracts were probed by immunoblotting with an anti-FLAG. Ponceau S staining (not shown) indicated comparable sample loading, as also indicated by a non-specific anti-FLAG band (*). The specific signal representing 3xFlag-nls-Cas9-nls (predicted molecular weight 164.67 kDa) is indicated as well (arrowhead).

**Additional file 25: Table S11**. Excel table with the source data used for figure preparation.

## Data Availability

The datasets generated during the current study are available in the Gene Expression Omnibus (GEO) repository (GEO Series accession number GSE159174; https://www.ncbi.nlm.nih.gov/geo/query/acc.cgi?acc=GSE159174). Additional data generated during this study are included in this published article and its supplementary information files.

## Abbreviations

AMP: Adult muscle precursor
APA: Alternative polyadenylation
ATAC-Seq: Assay for transposase-accessible chromatin using sequencing
bp: Base pairs
COR: Cold response genes
CRE: *Cis*-regulatory element
DCV: *Drosophila* C virus
DSCP: *Drosophila* synthetic core promoter
dsRNA: Double stranded ribonucleic acid
EGFP: Enhanced green fluorescent protein
EGFR: Epidermal growth factor receptor
ER: Endoplasmatic reticulum
ETS: E26 transformation-specific
FACS: Fluorescence activated cell sorting
FDR: False discovery rate
FGFR: Fibroblast growth factor receptor
*FLC*: *Flowering Locus C*
GFP: Green fluorescent protein
GO: Gene ontology
HSF1: Heat shock factor 1
HSP: Heat shock protein
HSR: Heat shock response
ICE-CBF: Inducer of CBF expression - C-repeat binding factors
JAK/STAT: Janus kinase/signal transducer and activator of transcription
JNK: c-Jun N-terminal kinase
LPS: Lipolysaccharide
MAP: Mitogen-activated protein kinase
mEGFP: Monomeric enhanced green fluorescent protein
PAS: Polyadenylation site
PBS: Phosphate buffered saline
PIF: Phytochrome interacting factor
*pnt*: *Pointed*
*pst*: *Pastrel*
qRT-PCR: Quantitative reverse transcriptase polymerase chain reaction
RNAi: Ribonucleic acid interference
RMCE: Recombinase-mediated cassette exchange
RTK: Receptor tyrosine kinase
s.d.: Standard deviation
STARR-Seq: Self-transcribing active regulatory region sequencing
TCA: Tricarboxylic acid
TF: Transcription factor
TOR: Target of rapamycin
TSS: Transcriptional start site
WGS: Whole genome sequencing

## References

[1] Ritossa F. A new puffing pattern induced by temperature shock and DNP in Drosophila. Experientia. 1962;18(12):571–3. 10.1007/BF02172188.

[2] Vihervaara A, Duarte FM, Lis JT. Molecular mechanisms driving transcriptional stress responses. Nat Rev Genet. 2018;19(6):385–97. 10.1038/s41576-018-0001-6. 10.1038/s41576-018-0001-6 PMC6036639 29556092

[3] Gomez-Pastor R, Burchfiel ET, Thiele DJ. Regulation of heat shock transcription factors and their roles in physiology and disease. Nature reviews. Mol Cell Biol. 2018;19(1):4–19. 10.1038/nrm.2017.73. 10.1038/nrm.2017.73 PMC5794010 28852220

[4] Liu AY, Bian H, Huang LE, Lee YK. Transient cold shock induces the heat shock response upon recovery at 37 degrees C in human cells. J Biol Chem. 1994;269(20):14768–75. 10.1016/S0021-9258(17)36691-7. 8182082

[5] Colinet H, Hoffmann A. Gene and protein expression of Drosophila Starvin during cold stress and recovery from chill coma. Insect Biochem Mol Biol. 2010;40(5):425–8. 10.1016/j.ibmb.2010.03.002. 10.1016/j.ibmb.2010.03.002 20303406

[6] Štětina T, Koštál V, Korbelová J. The role of inducible Hsp70, and other heat shock proteins, in adaptive complex of cold tolerance of the fruit Fly (Drosophila melanogaster). PLoS One. 2015;10(6):e0128976. 10.1371/journal.pone.0128976. 10.1371/journal.pone.0128976 PMC4452724 26034990

[7] von Heckel K, Stephan W, Hutter S. Canalization of gene expression is a major signature of regulatory cold adaptation in temperate Drosophila melanogaster. BMC Genom. 2016;17(1):574. 10.1186/s12864-016-2866-0. 10.1186/s12864-016-2866-0 PMC4977637 27502401

[8] Königer A, Grath S. Transcriptome analysis reveals candidate genes for cold tolerance in Drosophila ananassae. Genes. 2018;9(12):624. 10.3390/genes9120624. 10.3390/genes9120624 PMC6315829 30545157

[9] Mahat DB, Salamanca HH, Duarte FM, Danko CG, Lis JT. Mammalian heat shock response and mechanisms underlying its genome-wide transcriptional regulation. Mol Cell. 2016;62(1):63–78. 10.1016/j.molcel.2016.02.025. 10.1016/j.molcel.2016.02.025 PMC4826300 27052732

[10] Solís EJ, Pandey JP, Zheng X, Jin DX, Gupta PB, Airoldi EM, et al. Defining the essential function of yeast Hsf1 reveals a compact transcriptional program for maintaining eukaryotic Proteostasis. Mol Cell. 2016;63(1):60–71. 10.1016/j.molcel.2016.05.014. 10.1016/j.molcel.2016.05.014 PMC4938784 27320198

[11] Abduljalil JM. Bacterial riboswitches and RNA thermometers: nature and contributions to pathogenesis. Noncoding RNA Res. 2018;3(2):54–63. 10.1016/j.ncrna.2018.04.003. 10.1016/j.ncrna.2018.04.003 PMC6096418 30159440

[12] Weber MH, Marahiel MA. Bacterial cold shock responses. Sci Prog. 2003;86(1-2):9–75. 10.3184/003685003783238707. 10.3184/003685003783238707 PMC10368357 12838604

[13] Ding Y, Shi Y, Yang S. Molecular regulation of plant responses to environmental temperatures. Mol Plant. 2020;13(4):544–64. 10.1016/j.molp.2020.02.004. 10.1016/j.molp.2020.02.004 32068158

[14] Ritonga FN, Chen S. Physiological and molecular mechanism involved in cold stress tolerance in plants. Plants (Basel). 2020;9(5):560. 10.3390/plants9050560. 10.3390/plants9050560 PMC7284489 32353940

[15] Whittaker C, Dean C. The FLC locus: a platform for discoveries in epigenetics and adaptation. Annu Rev Cell Dev Biol. 2017;33(1):555–75. 10.1146/annurev-cellbio-100616-060546. 10.1146/annurev-cellbio-100616-060546 28693387

[16] Zhao Y, Antoniou-Kourounioti RL, Calder G, Dean C, Howard M. Temperature-dependent growth contributes to long-term cold sensing. Nature. 2020;583(7818):825–9. 10.1038/s41586-020-2485-4. 10.1038/s41586-020-2485-4 PMC7116785 32669706

[17] Quint M, Delker C, Franklin KA, Wigge PA, Halliday KJ, van Zanten M. Molecular and genetic control of plant thermomorphogenesis. Nat Plants. 2016;2(1):15190. 10.1038/nplants.2015.190. 10.1038/nplants.2015.190 27250752

[18] Chung BYW, Balcerowicz M, Di Antonio M, Jaeger KE, Geng F, Franaszek K, et al. An RNA thermoswitch regulates daytime growth in Arabidopsis. Nat Plants. 2020;6(5):522–32. 10.1038/s41477-020-0633-3. 10.1038/s41477-020-0633-3 PMC7231574 32284544

[19] Cortijo S, Charoensawan V, Brestovitsky A, Buning R, Ravarani C, Rhodes D, et al. Transcriptional regulation of the ambient temperature response by H2A.Z nucleosomes and HSF1 transcription factors in Arabidopsis. Mol Plant. 2017;10(10):1258–73. 10.1016/j.molp.2017.08.014. 10.1016/j.molp.2017.08.014 PMC6175055 28893714

[20] Kumar SV, Wigge PA. H2A.Z-containing nucleosomes mediate the thermosensory response in Arabidopsis. Cell. 2010;140(1):136–47. 10.1016/j.cell.2009.11.006. 10.1016/j.cell.2009.11.006 20079334

[21] Petavy D, Gibert M. Viability and rate of development at different temperatures in Drosophila: a comparison of constant and alternating thermal regimes. J Therm Biol. 2001;26:29–39. 10.1016/s0306-4565(00)00022-x. 10.1016/s0306-4565(00)00022-x 11070342

[22] Denlinger DL, Lee RE, editors. Low temperature biology of insects. Cambridge: Cambridge University Press; 2010. 10.1017/CBO9780511675997.

[23] Hoffmann AA, Sorensen JG, Loeschke V. Adaptation of Drosophila to temperature extremes: bringing together quantitative and molecular approaches. J Therm Biol. 2003;28(3):175–216. 10.1016/S0306-4565(02)00057-8.

[24] George R, Stanewsky R. Peripheral sensory organs contribute to temperature synchronization of the circadian clock in Drosophila melanogaster. Front Physiol. 2021;12:622545. 10.3389/fphys.2021.622545. 10.3389/fphys.2021.622545 PMC7884628 33603678

[25] Shakhmantsir I, Sehgal A. Splicing the clock to maintain and entrain circadian rhythms. J Biol Rhythm. 2019;34(6):584–95. 10.1177/0748730419868136. 10.1177/0748730419868136 PMC10823935 31389290

[26] Martin Anduaga A, Evantal N, Patop IL, Bartok O, Weiss R, Kadener S. Thermosensitive alternative splicing senses and mediates temperature adaptation in Drosophila. eLife. 2019;8:e44642. 10.7554/eLife.44642. 10.7554/eLife.44642 PMC6890466 31702556

[27] Chen J, Nolte V, Schlötterer C. Temperature stress mediates decanalization and dominance of gene expression in Drosophila melanogaster. PLoS Genet. 2015;11(2):e1004883. 10.1371/journal.pgen.1004883. 10.1371/journal.pgen.1004883 PMC4342254 25719753

[28] Jakšić AM, Schlötterer C. The interplay of temperature and genotype on patterns of alternative splicing in Drosophila melanogaster. Genetics. 2016;204(1):315–25. 10.1534/genetics.116.192310. 10.1534/genetics.116.192310 PMC5012396 27440867

[29] Fast I, Hewel C, Wester L, Schumacher J, Gebert D, Zischler H, et al. Temperature-responsive miRNAs in Drosophila orchestrate adaptation to different ambient temperatures. RNA. 2017;23(9):1352–64. 10.1261/rna.061119.117. 10.1261/rna.061119.117 PMC5558905 28630141

[30] Afik S, Bartok O, Artyomov MN, Shishkin AA, Kadri S, Hanan M, et al. Defining the 5΄ and 3΄ landscape of the Drosophila transcriptome with Exo-seq and RNaseH-seq. Nucleic Acids Res. 2017;45(11):e95. 10.1093/nar/gkx133. 10.1093/nar/gkx133 PMC5499799 28335028

[31] Yanagawa S, Lee JS, Ishimoto A. Identification and characterization of a novel line of Drosophila Schneider S2 cells that respond to wingless signaling. J Biol Chem. 1998;273(48):32353–9. 10.1074/jbc.273.48.32353. 10.1074/jbc.273.48.32353 9822716

[32] Buenrostro JD, Giresi PG, Zaba LC, Chang HY, Greenleaf WJ. Transposition of native chromatin for fast and sensitive epigenomic profiling of open chromatin, DNA-binding proteins and nucleosome position. Nat Methods. 2013;10(12):1213–8. 10.1038/nmeth.2688. 10.1038/nmeth.2688 PMC3959825 24097267

[33] Moll P, Ante M, Seitz A, Reda T. QuantSeq 3′ mRNA sequencing for RNA quantification. Nat Methods. 2014;11:i–iii. 10.1038/nmeth.f.376.

[34] Simcox A, Mitra S, Truesdell S, Paul L, Chen T, Butchar JP, et al. Efficient genetic method for establishing Drosophila cell lines unlocks the potential to create lines of specific genotypes. PLoS Genet. 2008;4(8):e1000142. 10.1371/journal.pgen.1000142. 10.1371/journal.pgen.1000142 PMC2474701 18670627

[35] Cherbas L, Willingham A, Zhang D, Yang L, Zou Y, Eads BD, et al. The transcriptional diversity of 25 Drosophila cell lines. Genome Res. 2011;21(2):301–14. 10.1101/gr.112961.110. 10.1101/gr.112961.110 PMC3032933 21177962

[36] Dequéant M-L, Fagegaltier D, Hu Y, Spirohn K, Simcox A, Hannon GJ, et al. Discovery of progenitor cell signatures by time-series synexpression analysis during Drosophila embryonic cell immortalization. Proc Natl Acad Sci U S A. 2015;112(42):12974–9. 10.1073/pnas.1517729112. 10.1073/pnas.1517729112 PMC4620889 26438832

[37] Gunage RD, Dhanyasi N, Reichert H, Vijayraghavan K. Drosophila adult muscle development and regeneration. Semin Cell Dev Biol. 2017;72:56–66. 10.1016/j.semcdb.2017.11.017. 10.1016/j.semcdb.2017.11.017 29146144

[38] Banerjee U, Girard JR, Goins LM, Spratford CM. Drosophila as a genetic model for hematopoiesis. Genetics. 2019;211(2):367–417. 10.1534/genetics.118.300223. 10.1534/genetics.118.300223 PMC6366919 30733377

[39] Rot G, Wang Z, Huppertz I, Modic M, Lenče T, Hallegger M, et al. High-resolution RNA maps suggest common principles of splicing and polyadenylation regulation by TDP-43. Cell Rep. 2017;19(5):1056–67. 10.1016/j.celrep.2017.04.028. 10.1016/j.celrep.2017.04.028 PMC5437728 28467899

[40] Tian B, Manley JL. Alternative polyadenylation of mRNA precursors. Nature reviews. Mol Cell Biol. 2017;18(1):18–30. 10.1038/nrm.2016.116. 10.1038/nrm.2016.116 PMC5483950 27677860

[41] Gruber AJ, Zavolan M. Alternative cleavage and polyadenylation in health and disease. Nat Rev Genet. 2019;20(10):599–614. 10.1038/s41576-019-0145-z. 10.1038/s41576-019-0145-z 31267064

[42] Sadek J, Omer A, Hall D, Ashour K, Gallouzi IE. Alternative polyadenylation and the stress response. Wiley Interdiscip Rev RNA. 2019;10(5):e1540. 10.1002/wrna.1540. 10.1002/wrna.1540 31050180

[43] Kortmann J, Narberhaus F. Bacterial RNA thermometers: molecular zippers and switches. Nat Rev Microbiol. 2012;10(4):255–65. 10.1038/nrmicro2730. 10.1038/nrmicro2730 22421878

[44] Somero GN. RNA thermosensors: how might animals exploit their regulatory potential? J Exp Biol. 2018;221(4):jeb162842. 10.1242/jeb.162842. 10.1242/jeb.162842 29472490

[45] Goutte C, Toft P, Rostrup E, Nielsen F, Hansen LK. On clustering fMRI time series. Neuroimage. 1999;9(3):298–310. 10.1006/nimg.1998.0391. 10.1006/nimg.1998.0391 10075900

[46] Musselman LP, Kühnlein RP. Drosophila as a model to study obesity and metabolic disease. J Exp Biol. 2018;221(Suppl_1):jeb163881. 10.1242/jeb.163881. 10.1242/jeb.163881 29514880

[47] Mattila J, Hietakangas V. Regulation of carbohydrate energy metabolism in Drosophila melanogaster. Genetics. 2017;207(4):1231–53. 10.1534/genetics.117.199885. 10.1534/genetics.117.199885 PMC5714444 29203701

[48] Palanker L, Tennessen JM, Lam G, Thummel CS. Drosophila HNF4 regulates lipid mobilization and beta-oxidation. Cell Metab. 2009;9(3):228–39. 10.1016/j.cmet.2009.01.009. 10.1016/j.cmet.2009.01.009 PMC2673486 19254568

[49] Clark RI, Tan SWS, Péan CB, Roostalu U, Vivancos V, Bronda K, et al. MEF2 is an in vivo immune-metabolic switch. Cell. 2013;155(2):435–47. 10.1016/j.cell.2013.09.007. 10.1016/j.cell.2013.09.007 PMC3807682 24075010

[50] Gonsalves SE, Moses AM, Razak Z, Robert F, Westwood JT. Whole-genome analysis reveals that active heat shock factor binding sites are mostly associated with non-heat shock genes in Drosophila melanogaster. PLoS One. 2011;6(1):e15934. 10.1371/journal.pone.0015934. 10.1371/journal.pone.0015934 PMC3021535 21264254

[51] Duarte FM, Fuda NJ, Mahat DB, Core LJ, Guertin MJ, Lis JT. Transcription factors GAF and HSF act at distinct regulatory steps to modulate stress-induced gene activation. Genes Dev. 2016;30(15):1731–46. 10.1101/gad.284430.116. 10.1101/gad.284430.116 PMC5002978 27492368

[52] Radermacher PT, Myachina F, Bosshardt F, Pandey R, Mariappa D, Muller HA, et al. O-GlcNAc reports ambient temperature and confers heat resistance on ectotherm development. Proc Natl Acad Sci U S A. 2014;111(15):5592–7. 10.1073/pnas.1322396111. 10.1073/pnas.1322396111 PMC3992692 24706800

[53] Myachina F, Bosshardt F, Bischof J, Kirschmann M, Lehner CF. Drosophila β-tubulin 97EF is upregulated at low temperature and stabilizes microtubules. Development. 2017;144:4573–87. 10.1242/dev.156109. 10.1242/dev.156109 29084803

[54] Goto SG. Expression of Drosophila homologue of senescence marker protein-30 during cold acclimation. J Insect Physiol. 2000;46(7):1111–20. 10.1016/S0022-1910(99)00221-8. 10.1016/s0022-1910(99)00221-8 10817837

[55] Baird L, Yamamoto M. The molecular mechanisms regulating the KEAP1-NRF2 pathway. Mol Cell Biol. 2020;40(13):e00099–20. 10.1128/MCB.00099-20. 10.1128/MCB.00099-20 PMC7296212 32284348

[56] Sykiotis GP, Bohmann D. Keap1/Nrf2 signaling regulates oxidative stress tolerance and lifespan in Drosophila. Dev Cell. 2008;14(1):76–85. 10.1016/j.devcel.2007.12.002. 10.1016/j.devcel.2007.12.002 PMC2257869 18194654

[57] Külshammer E, Mundorf J, Kilinc M, Frommolt P, Wagle P, Uhlirova M. Interplay among Drosophila transcription factors Ets21c, Fos and Ftz-F1 drives JNK-mediated tumor malignancy. Dis Model Mech. 2015;8:1279–93. 10.1242/dmm.020719. 10.1242/dmm.020719 PMC4610234 26398940

[58] Girardot F, Monnier V, Tricoire H. Genome wide analysis of common and specific stress responses in adult drosophila melanogaster. BMC Genomics. 2004;5(1):74. 10.1186/1471-2164-5-74. 10.1186/1471-2164-5-74 PMC526417 15458575

[59] de Gregorio E, Spellman PT, Rubin GM, Lemaitre B. Genome-wide analysis of the Drosophila immune response by using oligonucleotide microarrays. Proc Natl Acad Sci U S A. 2001;98(22):12590–5. 10.1073/pnas.221458698. 10.1073/pnas.221458698 PMC60098 11606746

[60] Landis GN, Abdueva D, Skvortsov D, Yang J, Rabin BE, Carrick J, et al. Similar gene expression patterns characterize aging and oxidative stress in Drosophila melanogaster. Proc Natl Acad Sci U S A. 2004;101(20):7663–8. 10.1073/pnas.0307605101. 10.1073/pnas.0307605101 PMC419663 15136717

[61] Zinke I, Schütz CS, Katzenberger JD, Bauer M, Pankratz MJ. Nutrient control of gene expression in Drosophila: microarray analysis of starvation and sugar-dependent response. EMBO J. 2002;21(22):6162–73. 10.1093/emboj/cdf600. 10.1093/emboj/cdf600 PMC137192 12426388

[62] Gonda RL, Garlena RA, Stronach B. Drosophila heat shock response requires the JNK pathway and phosphorylation of mixed lineage kinase at a conserved serine-proline motif. PLoS One. 2012;7(7):e42369. 10.1371/journal.pone.0042369. 10.1371/journal.pone.0042369 PMC3407086 22848763

[63] Pfeiffer BD, Jenett A, Hammonds AS, Ngo TT, Misra S, Murphy C, et al. Tools for neuroanatomy and neurogenetics in Drosophila. Proc Natl Acad Sci U S A. 2008;105(28):9715–20. 10.1073/pnas.0803697105. 10.1073/pnas.0803697105 PMC2447866 18621688

[64] Arnold CD, Gerlach D, Spies D, Matts JA, Sytnikova YA, Pagani M, et al. Quantitative genome-wide enhancer activity maps for five Drosophila species show functional enhancer conservation and turnover during cis-regulatory evolution. Nat Genet. 2014;46(7):685–92. 10.1038/ng.3009. 10.1038/ng.3009 PMC4250274 24908250

[65] Brand AH, Perrimon N. Targeted gene expression as a means of altering cell fates and generating dominant phenotypes. Development. 1993;118(2):401–15. 10.1242/dev.118.2.401. 10.1242/dev.118.2.401 8223268

[66] Yáñez-Cuna JO, Arnold CD, Stampfel G, Boryń LM, Gerlach D, Rath M, et al. Dissection of thousands of cell type-specific enhancers identifies dinucleotide repeat motifs as general enhancer features. Genome Res. 2014;24(7):1147–56. 10.1101/gr.169243.113. 10.1101/gr.169243.113 PMC4079970 24714811

[67] Cherbas L, Hackney J, Gong L, Salzer C, Mauser E, Zhang D, et al. Tools for targeted genome engineering of established Drosophila cell lines. Genetics. 2015;201(4):1307–18. 10.1534/genetics.115.181610. 10.1534/genetics.115.181610 PMC4676523 26450921

[68] Pauli D, Spierer A, Tissières A. Several hundred base pairs upstream of Drosophila hsp23 and 26 genes are required for their heat induction in transformed flies. EMBO J. 1986;5(4):755–61. 10.1002/j.1460-2075.1986.tb04278.x. 10.1002/j.1460-2075.1986.tb04278.x PMC1166855 3011424

[69] Grant CE, Bailey TL, Noble WS. FIMO: scanning for occurrences of a given motif. Bioinformatics. 2011;27(7):1017–8. 10.1093/bioinformatics/btr064. 10.1093/bioinformatics/btr064 PMC3065696 21330290

[70] Hsu T, Schulz RA. Sequence and functional properties of Ets genes in the model organism Drosophila. Oncogene. 2000;19(55):6409–16. 10.1038/sj.onc.1204033. 10.1038/sj.onc.1204033 11175357

[71] Herrera SC, Bach EA. JAK/STAT signaling in stem cells and regeneration: from Drosophila to vertebrates. Development. 2019;146(2):dev167643. 10.1242/dev.167643. 10.1242/dev.167643 PMC6361132 30696713

[72] Zhu LJ, Christensen RG, Kazemian M, Hull CJ, Enuameh MS, Basciotta MD, et al. FlyFactorSurvey: a database of Drosophila transcription factor binding specificities determined using the bacterial one-hybrid system. Nucleic Acids Res. 2011;39(suppl_1):D111–7. 10.1093/nar/gkq858. 10.1093/nar/gkq858 PMC3013762 21097781

[73] Sopko R, Perrimon N. Receptor tyrosine kinases in Drosophila development. Cold Spring Harb Perspect Biol. 2013;5(6):a009050. 10.1101/cshperspect.a009050. 10.1101/cshperspect.a009050 PMC3660834 23732470

[74] Lee H, McManus CJ, Cho D-Y, Eaton M, Renda F, Somma MP, et al. DNA copy number evolution in Drosophila cell lines. Genome Biol. 2014;15(8):R70. 10.1186/gb-2014-15-8-r70. 10.1186/gb-2014-15-8-r70 PMC4289277 25262759

[75] Wartlick O, Mumcu P, Kicheva A, Bittig T, Seum C, Jülicher F, et al. Dynamics of Dpp signaling and proliferation control. Science. 2011;331(6021):1154–9. 10.1126/science.1200037. 10.1126/science.1200037 21385708

[76] Guo Y, Flegel K, Kumar J, McKay DJ, Buttitta LA. Ecdysone signaling induces two phases of cell cycle exit in Drosophila cells. Biol Open. 2016;5(11):1648–61. 10.1242/bio.017525. 10.1242/bio.017525 PMC5155522 27737823

[77] Otsuki L, Brand AH. Cell cycle heterogeneity directs the timing of neural stem cell activation from quiescence. Science. 2018;360(6384):99–102. 10.1126/science.aan8795. 10.1126/science.aan8795 PMC6538531 29622651

[78] Cosolo A, Jaiswal J, Csordás G, Grass I, Uhlirova M, Classen A-K. JNK-dependent cell cycle stalling in G2 promotes survival and senescence-like phenotypes in tissue stress. 2019;8:eLife, e41036. 10.7554/eLife.41036. 10.7554/eLife.41036 PMC6389326 30735120

[79] Edgar BA, O'Farrell PH. Genetic control of cell division patterns in the Drosophila embryo. Cell. 1989;57(1):177–83. 10.1016/0092-8674(89)90183-9. 10.1016/0092-8674(89)90183-9 PMC2755076 2702688

[80] Simcox A. Progress towards Drosophila epithelial cell culture. Methods Mol Biol. 2013;945:1–11. 10.1007/978-1-62703-125-7_1. 10.1007/978-1-62703-125-7_1 PMC4291111 23097097

[81] Adelman K, Lis JT. Promoter-proximal pausing of RNA polymerase II: emerging roles in metazoans. Nat Rev Genet. 2012;13(10):720–31. 10.1038/nrg3293. 10.1038/nrg3293 PMC3552498 22986266

[82] Manivannan SN, Jacobsen TL, Lyon P, Selvaraj B, Halpin P, Simcox A. Targeted integration of single-copy transgenes in Drosophila melanogaster tissue-culture cells using recombination-mediated cassette exchange. Genetics. 2015;201(4):1319–28. 10.1534/genetics.115.181230. 10.1534/genetics.115.181230 PMC4676529 26500255

[83] Neumüller RA, Wirtz-Peitz F, Lee S, Kwon Y, Buckner M, Hoskins RA, et al. Stringent analysis of gene function and protein-protein interactions using fluorescently tagged genes. Genetics. 2012;190(3):931–40. 10.1534/genetics.111.136465. 10.1534/genetics.111.136465 PMC3296255 22174071

[84] Viswanatha R, Li Z, Hu Y, Perrimon N. Pooled genome-wide CRISPR screening for basal and context-specific fitness gene essentiality in Drosophila cells. 2018;7:eLife, e36333. 10.7554/eLife.36333. 10.7554/eLife.36333 PMC6063728 30051818

[85] Sumitomo Y, Higashitsuji H, Higashitsuji H, Liu Y, Fujita T, Sakurai T, et al. Identification of a novel enhancer that binds Sp1 and contributes to induction of cold-inducible RNA-binding protein (cirp) expression in mammalian cells. BMC Biotechnol. 2012;12(1):72. 10.1186/1472-6750-12-72. 10.1186/1472-6750-12-72 PMC3534229 23046908

[86] Thaisuchat H, Baumann M, Pontiller J, Hesse F, Ernst W. Identification of a novel temperature sensitive promoter in CHO cells. BMC Biotechnol. 2011;11(1):51. 10.1186/1472-6750-11-51. 10.1186/1472-6750-11-51 PMC3118111 21569433

[87] Brunner D, Dücker K, Oellers N, Hafen E, Scholz H, Klämbt C. The ETS domain protein pointed-P2 is a target of MAP kinase in the sevenless signal transduction pathway. Nature. 1994;370(6488):386–9. 10.1038/370386a0. 10.1038/370386a0 8047146

[88] O'Neill EM, Rebay I, Tjian R, Rubin GM. The activities of two Ets-related transcription factors required for Drosophila eye development are modulated by the Ras/MAPK pathway. Cell. 1994;78(1):137–47. 10.1016/0092-8674(94)90580-0. 10.1016/0092-8674(94)90580-0 8033205

[89] Shilo B-Z. The regulation and functions of MAPK pathways in Drosophila. Methods. 2014;68(1):151–9. 10.1016/j.ymeth.2014.01.020. 10.1016/j.ymeth.2014.01.020 24530508

[90] Rosmarin AG, Resendes KK, Yang Z, McMillan JN, Fleming SL. GA-binding protein transcription factor: a review of GABP as an integrator of intracellular signaling and protein-protein interactions. Blood Cells Mol Dis. 2004;32(1):143–54. 10.1016/j.bcmd.2003.09.005. 10.1016/j.bcmd.2003.09.005 14757430

[91] Baltzer C, Tiefenböck SK, Marti M, Frei C. Nutrition controls mitochondrial biogenesis in the Drosophila adipose tissue through Delg and cyclin D/Cdk4. PLoS One. 2009;4(9):e6935. 10.1371/journal.pone.0006935. 10.1371/journal.pone.0006935 PMC2735006 19742324

[92] Frei C, Galloni M, Hafen E, Edgar BA. The Drosophila mitochondrial ribosomal protein mRpL12 is required for cyclin D/Cdk4-driven growth. EMBO J. 2005;24(3):623–34. 10.1038/sj.emboj.7600523. 10.1038/sj.emboj.7600523 PMC548645 15692573

[93] Schulz RA, The SM, Hogue DA, Galewsky S, Guo Q. Ets oncogene-related gene Elg functions in Drosophila oogenesis. Proc Natl Acad Sci U S A. 1993;90(21):10076–80. 10.1073/pnas.90.21.10076. 10.1073/pnas.90.21.10076 PMC47716 8234259

[94] Dobson AJ, Boulton-McDonald R, Houchou L, Svermova T, Ren Z, Subrini J, et al. Longevity is determined by ETS transcription factors in multiple tissues and diverse species. PLoS Genet. 2019;15(7):e1008212. 10.1371/journal.pgen.1008212. 10.1371/journal.pgen.1008212 PMC6662994 31356597

[95] Dubnau J, Chiang A-S, Grady L, Barditch J, Gossweiler S, McNeil J, et al. The staufen/pumilio pathway is involved in Drosophila long-term memory. Curr Biol. 2003;13(4):286–96. 10.1016/s0960-9822(03)00064-2. 10.1016/s0960-9822(03)00064-2 12593794

[96] Fukui A, Inaki M, Tonoe G, Hamatani H, Homma M, Morimoto T, et al. Lola regulates glutamate receptor expression at the Drosophila neuromuscular junction. Biol Open. 2012;1(4):362–75. 10.1242/bio.2012448. 10.1242/bio.2012448 PMC3509458 23213426

[97] Magwire MM, Fabian DK, Schweyen H, Cao C, Longdon B, Bayer F, et al. Genome-wide association studies reveal a simple genetic basis of resistance to naturally coevolving viruses in Drosophila melanogaster. PLoS Genet. 2012;8(11):e1003057. 10.1371/journal.pgen.1003057. 10.1371/journal.pgen.1003057 PMC3499358 23166512

[98] Cao C, Cogni R, Barbier V, Jiggins FM. Complex coding and regulatory polymorphisms in a restriction factor determine the susceptibility of Drosophila to viral infection. Genetics. 2017;206(4):2159–73. 10.1534/genetics.117.201970. 10.1534/genetics.117.201970 PMC5560813 28630113

[99] Yang J, Yan R, Roy A, Xu D, Poisson J, Zhang Y. The I-TASSER suite: protein structure and function prediction. Nat Methods. 2015;12(1):7–8. 10.1038/nmeth.3213. 10.1038/nmeth.3213 PMC4428668 25549265

[100] Lidsky PV, Sprenger F, Lehner CF. Distinct modes of centromere protein dynamics during cell cycle progression in Drosophila S2R+ cells. J Cell Sci. 2013;126:4782–93. 10.1242/jcs.134122. 10.1242/jcs.134122 23943877

[101] Herzog S, Nagarkar Jaiswal S, Urban E, Riemer A, Fischer S, Heidmann SK. Functional dissection of the Drosophila melanogaster condensin subunit cap-G reveals its exclusive association with condensin I. PLoS Genet. 2013;9(4):e1003463. 10.1371/journal.pgen.1003463. 10.1371/journal.pgen.1003463 PMC3630105 23637630

[102] Markstein M, Pitsouli C, Villalta C, Celniker SE, Perrimon N. Exploiting position effects and the gypsy retrovirus insulator to engineer precisely expressed transgenes. Nat Genet. 2008;40(4):476–83. 10.1038/ng.101. 10.1038/ng.101 PMC2330261 18311141

[103] Makarova O, Kamberov E, Margolis B. Generation of deletion and point mutations with one primer in a single cloning step. Biotechniques. 2000;29(5):970–2. 10.2144/00295bm08. 10.2144/00295bm08 11084856

[104] Huang J, Ghosh P, Hatfull GF, Hong Y. Successive and targeted DNA integrations in the Drosophila genome by Bxb1 and phiC31 integrases. Genetics. 2011;189(1):391–5. 10.1534/genetics.111.129247. 10.1534/genetics.111.129247 PMC3176133 21652525

[105] Edgar R, Domrachev M, Lash AE. Gene expression omnibus: NCBI gene expression and hybridization array data repository. Nucleic Acids Res. 2002;30(1):207–10. 10.1093/nar/30.1.207. 10.1093/nar/30.1.207 PMC99122 11752295

[106] Bolstad B. PreprocessCore: A collection of pre-processing functions. R package. DOI: 10.18129/B9.bioc.preprocessCore.

[107] Smyth GK. Limma: linear models for microarray data. In: Gentleman R, Carey V, Dudoit S, Irizarry R, Huber W, editors. Bioinformatics and computational biology solutions using R and Bioconductor. New York: Springer; 2005. p. 397–420. 10.1007/0-387-29362-0_23.

[108] Leek JT, Storey JD. Capturing heterogeneity in gene expression studies by surrogate variable analysis. PLoS Genet. 2007;3(9):1724–35. 10.1371/journal.pgen.0030161. 10.1371/journal.pgen.0030161 PMC1994707 17907809

[109] Baker KD, Thummel CS. Diabetic larvae and obese flies-emerging studies of metabolism in Drosophila. Cell Metab. 2007;6(4):257–66. 10.1016/j.cmet.2007.09.002. 10.1016/j.cmet.2007.09.002 PMC2231808 17908555

[110] Szklarczyk D, Gable AL, Lyon D, Junge A, Wyder S, Huerta-Cepas J, et al. STRING v11: protein-protein association networks with increased coverage, supporting functional discovery in genome-wide experimental datasets. Nucleic Acids Res. 2019;47(D1):D607–13. 10.1093/nar/gky1131. 10.1093/nar/gky1131 PMC6323986 30476243

[111] Dobin A, Davis CA, Schlesinger F, Drenkow J, Zaleski C, Jha S, et al. STAR: ultrafast universal RNA-seq aligner. Bioinformatics. 2013;29(1):15–21. 10.1093/bioinformatics/bts635. 10.1093/bioinformatics/bts635 PMC3530905 23104886

[112] Robinson MD, McCarthy DJ, Smyth GK. edgeR: a Bioconductor package for differential expression analysis of digital gene expression data. Bioinformatics. 2010;26(1):139–40. 10.1093/bioinformatics/btp616. 10.1093/bioinformatics/btp616 PMC2796818 19910308

[113] Rau A, Gallopin M, Celeux G, Jaffrézic F. Data-based filtering for replicated high-throughput transcriptome sequencing experiments. Bioinformatics. 2013;29(17):2146–52. 10.1093/bioinformatics/btt350. 10.1093/bioinformatics/btt350 PMC3740625 23821648

[114] Gruber AJ, Schmidt R, Gruber AR, Martin G, Ghosh S, Belmadani M, et al. A comprehensive analysis of 3′ end sequencing data sets reveals novel polyadenylation signals and the repressive role of heterogeneous ribonucleoprotein C on cleavage and polyadenylation. Genome Res. 2016;26(8):1145–59. 10.1101/gr.202432.115. 10.1101/gr.202432.115 PMC4971764 27382025

[115] Anders S, Reyes A, Huber W. Detecting differential usage of exons from RNA-seq data. Genome Res. 2012;22(10):2008–17. 10.1101/gr.133744.111. 10.1101/gr.133744.111 PMC3460195 22722343

[116] Wei T, Simko V (2017). R package "corrplot": Visualization of a Correlation Matrix (Version 0.84).

[117] Blighe K, Rana S, Lewis M (2020). EnhancedVolcano: Publication-ready volcano plots with enhanced colouring and labeling. R package version 1.6.0.

[118] Bolger AM, Lohse M, Usadel B. Trimmomatic: a flexible trimmer for Illumina sequence data. Bioinformatics. 2014;30(15):2114–20. 10.1093/bioinformatics/btu170. 10.1093/bioinformatics/btu170 PMC4103590 24695404

[119] Langmead B, Wilks C, Antonescu V, Charles R. Scaling read aligners to hundreds of threads on general-purpose processors. Bioinformatics. 2019;35(3):421–32. 10.1093/bioinformatics/bty648. 10.1093/bioinformatics/bty648 PMC6361242 30020410

[120] Li H, Handsaker B, Wysoker A, Fennell T, Ruan J, Homer N, et al. The sequence alignment/map format and SAMtools. Bioinformatics. 2009;25(16):2078–9. 10.1093/bioinformatics/btp352. 10.1093/bioinformatics/btp352 PMC2723002 19505943

[121] Li H. A statistical framework for SNP calling, mutation discovery, association mapping and population genetical parameter estimation from sequencing data. Bioinformatics. 2011;27(21):2987–93. 10.1093/bioinformatics/btr509. 10.1093/bioinformatics/btr509 PMC3198575 21903627

[122] Zhang Y, Liu T, Meyer CA, Eeckhoute J, Johnson DS, Bernstein BE, et al. Model-based analysis of ChIP-Seq (MACS). Genome Biol. 2008;9(9):R137. 10.1186/gb-2008-9-9-r137. 10.1186/gb-2008-9-9-r137 PMC2592715 18798982

[123] Stark R, Brown G. DiffBind: differential binding analysis of ChIP-Seq peak data; 2011. 10.18129/B9.bioc.DiffBind.

[124] Love MI, Huber W, Anders S. Moderated estimation of fold change and dispersion for RNA-seq data with DESeq2. Genome Biol. 2014;15(12):550. 10.1186/s13059-014-0550-8. 10.1186/s13059-014-0550-8 PMC4302049 25516281

[125] Causton HC, Quackenbush J, Brazma A (2003). Microarray gene expression data analysis: a beginner's guide / Helen C. Causton, John Quackenbush and Alvis Brazma.

[126] Simpson JT. Exploring genome characteristics and sequence quality without a reference. Bioinformatics. 2014;30(9):1228–35. 10.1093/bioinformatics/btu023. 10.1093/bioinformatics/btu023 PMC3998141 24443382

[127] Bankevich A, Nurk S, Antipov D, Gurevich AA, Dvorkin M, Kulikov AS, et al. SPAdes: a new genome assembly algorithm and its applications to single-cell sequencing. J Comput Biol. 2012;19(5):455–77. 10.1089/cmb.2012.0021. 10.1089/cmb.2012.0021 PMC3342519 22506599

[128] Simpson JT, Wong K, Jackman SD, Schein JE, Jones SJM, Birol I. ABySS: a parallel assembler for short read sequence data. Genome Res. 2009;19(6):1117–23. 10.1101/gr.089532.108. 10.1101/gr.089532.108 PMC2694472 19251739

[129] Gurevich A, Saveliev V, Vyahhi N, Tesler G. QUAST: quality assessment tool for genome assemblies. Bioinformatics. 2013;29(8):1072–5. 10.1093/bioinformatics/btt086. 10.1093/bioinformatics/btt086 PMC3624806 23422339

[130] Li H, Durbin R. Fast and accurate short read alignment with burrows-wheeler transform. Bioinformatics. 2009;25(14):1754–60. 10.1093/bioinformatics/btp324. 10.1093/bioinformatics/btp324 PMC2705234 19451168

[131] Faust GG, Hall IM. SAMBLASTER: fast duplicate marking and structural variant read extraction. Bioinformatics. 2014;30(17):2503–5. 10.1093/bioinformatics/btu314. 10.1093/bioinformatics/btu314 PMC4147885 24812344

[132] Layer RM, Chiang C, Quinlan AR, Hall IM. LUMPY: a probabilistic framework for structural variant discovery. Genome Biol. 2014;15(6):R84. 10.1186/gb-2014-15-6-r84. 10.1186/gb-2014-15-6-r84 PMC4197822 24970577

[133] Rausch T, Zichner T, Schlattl A, Stütz AM, Benes V, Korbel JO. DELLY: structural variant discovery by integrated paired-end and split-read analysis. Bioinformatics. 2012;28(18):i333–9. 10.1093/bioinformatics/bts378. 10.1093/bioinformatics/bts378 PMC3436805 22962449

[134] Abyzov A, Urban AE, Snyder M, Gerstein M. CNVnator: an approach to discover, genotype, and characterize typical and atypical CNVs from family and population genome sequencing. Genome Res. 2011;21(6):974–84. 10.1101/gr.114876.110. 10.1101/gr.114876.110 PMC3106330 21324876

