# Correction to: A *cis*-regulatory element promoting increased transcription at low temperature in cultured ectothermic *Drosophila* cells

**DOI:** 10.1186/s12864-022-08473-0

**Published:** 2022-03-28

**Authors:** Yu Bai, Emmanuel Caussinus, Stefano Leo, Fritz Bosshardt, Faina Myachina, Gregor Rot, Mark D. Robinson, Christian F. Lehner

**Affiliations:** 1 Department of Molecular Life Sciences, University of Zurich, Winterthurerstrasse 190, 8057 Zurich, Switzerland; 2 SIB Swiss Institute of Bioinformatics, University of Zurich, Winterthurerstrasse 190, 8057 Zurich, Switzerland

**Correction to: BMC Genomics 22, 771 (2021)**

**https://doi.org/10.1186/s12864-021-08057-4**

Following the publication of the original article [1], the corresponding author was informed of two mistakes present in the originally published Additional file 23 Table S10. The sequences of the oligonucleotides YB320 and YB362, which were used as forward primers for amplification of the candidate CREs *Hsp23*_E2 and *Prx2540-1*_E, were not entered correctly into this table.

The correct sequence of YB320 is 5’-TAGTTGGGGATGTCTTCGAATGTACATATGTTCCAAATCG -3’ and of YB362 is 5’- TAGTTGGGGATGTCTTCCATTTAGCTCATCTCCACGCTAG -3’.

The correct Additional file 23 Table S10 is included in this Correction article and the original article [1] has been updated.

## Supplementary Information

**Additional file 23: Table S10**. Excel file with description of synthetic DNA fragments (oligos and gene blocks).
