# Supplementary figures and images for: A cis-regulatory element promoting increased transcription at low temperature in cultured ectothermic Drosophila cells
Source: BMC Genomics. 2021 Oct 28;22:771. doi: 10.1186/s12864-021-08057-4 (PMC8555087; doi:10.1186/s12864-021-08057-4)

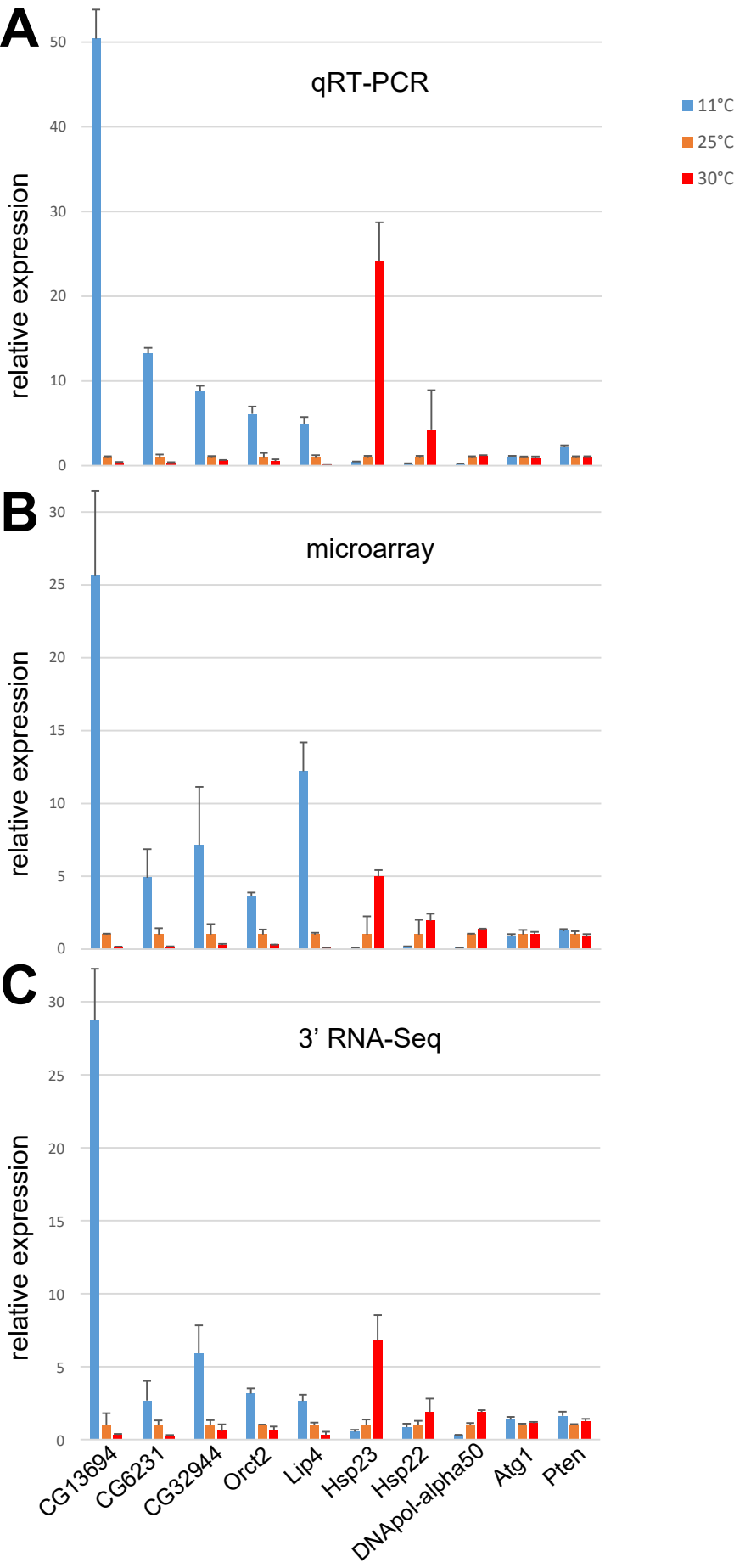

Supplement: Supplementary file 3 — Additional file 3: Fig. S1. Concordance of transcript levels after quantification with different methods. (A-C) Temperature effects on transcript levels in S2R+ cells were analyzed using either qRT-PCR (A), microarrays (B) or 3′ RNA-Seq (C). The bar diagram displays relative expression levels at the different temperatures, as detected by the different methods. Expression at 25 °C was set to 1. For each analysis, culture aliquots were shifted for 24 h to the indicated temperatures (11, 25 and 30 °C) before RNA isolation. Representative CoolUp genes (CG13694, CG6321, CG32944, Orct2, Lip4) and CoolDown genes (Hsp23, Hsp22, DNApol-α50), as well as genes (Atg1, Pten), which were barely temperature regulated, were selected for validation by qRT-PCR. In case of qRT-PCR, mean and s.d. of three technical replicates are displayed. In case of the microarray data, mean and s.d. of three biological replicates and multiple probes, if present, are shown. In case of the 3′ RNA-Seq data, mean and s.d. of three biological replicates are presented. [file 12864_2021_8057_MOESM3_ESM.pdf]

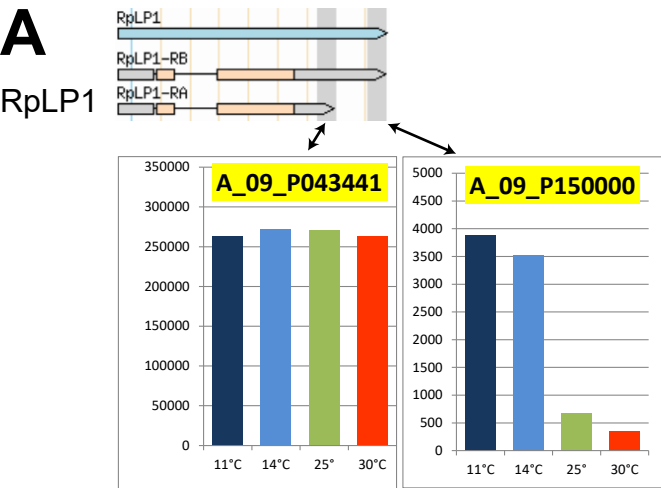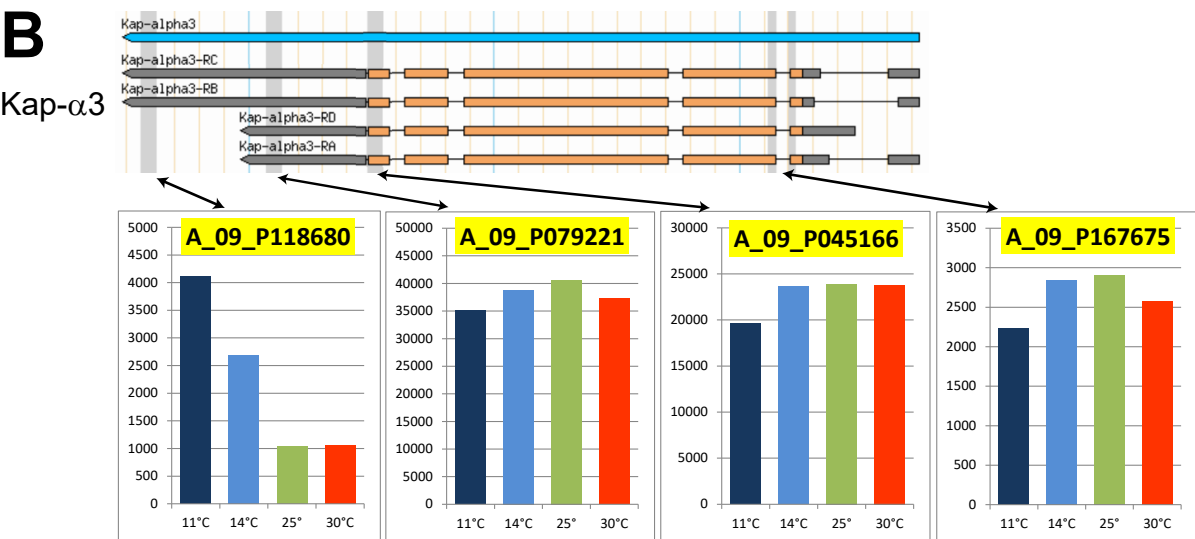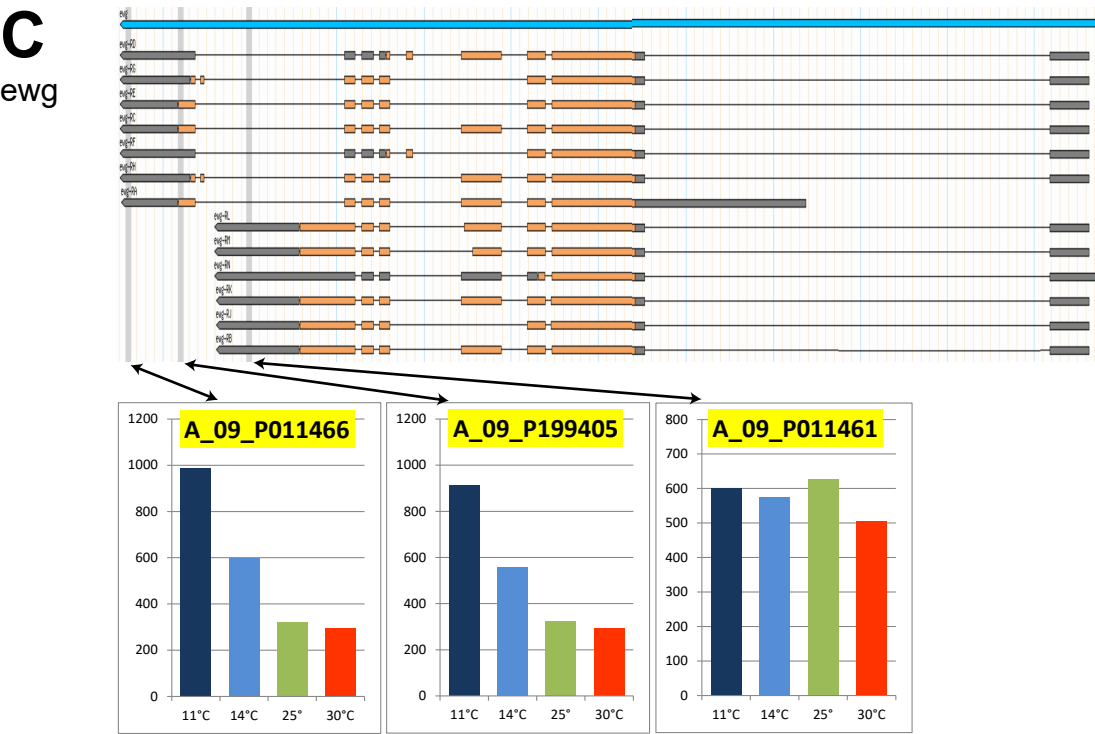

Supplement: Supplementary file 4 — Additional file 4: Fig. S2. Increased use of distal polyadenylation sites at low temperature. (A-C) Microarray probes with pronounced CoolUp signals were observed to detect specifically the annotated transcripts with the longest 3′ untranslated region, as illustrated in case of the gene (A) RpLP1, (B) Kap-α3 and (C) ewg. The positions recognized by different probes are indicated (vertical bars of light grey shading) in a scheme with the transcribed region and the annotated transcripts. The bar diagrams below display signal intensities (mean of three biological replicates) observed with these probes after incubation of S2R+ cells at different temperatures (11, 14, 25 and 30 °C). [file 12864_2021_8057_MOESM4_ESM.pdf]

**A****S2R+**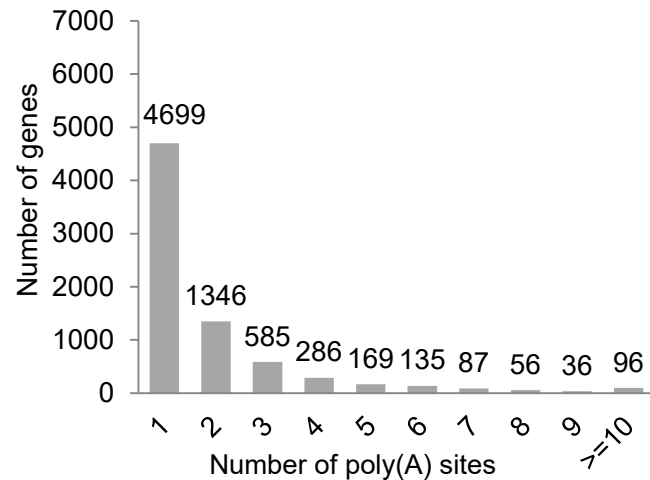**B****adult male flies**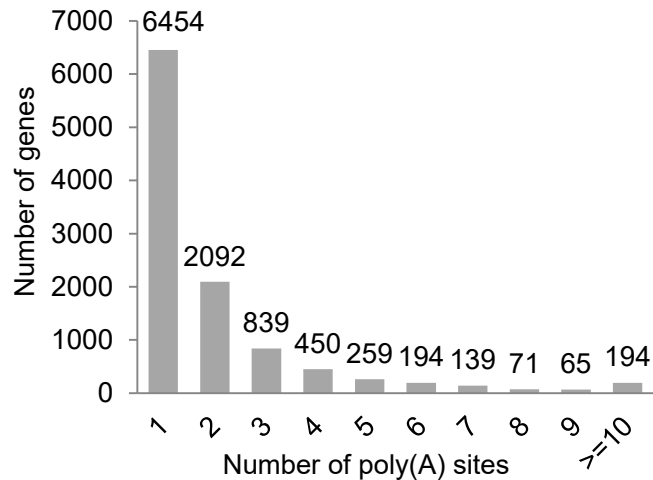**C****S2R+**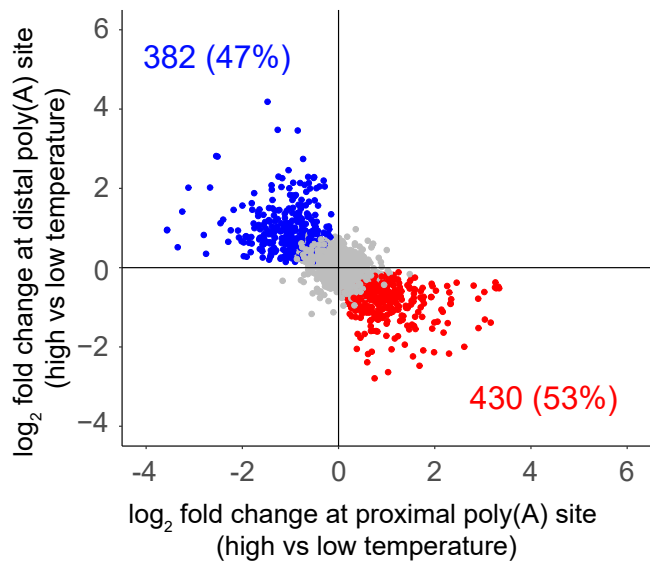**D****adult male flies**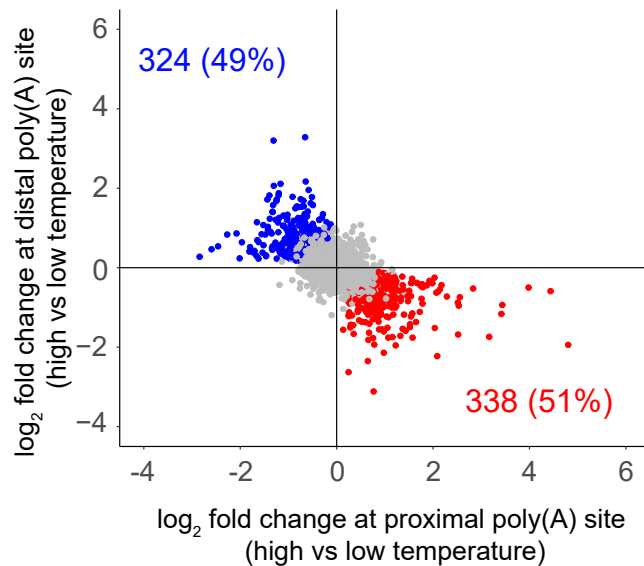

Supplement: Supplementary file 9 — Additional file 9: Fig. S3. Temperature-regulated alternative polyadenylation. (A-D) 3′ RNA-Seq data obtained from S2R+ cells (A, C) and adult male flies (B, D) after 24 h of incubation at different temperatures (11, 14, 25 and 30 °C) was used for an analysis of temperature effects on the choice of polyadenylation sites (PASs). (A,B) Histograms illustrate the frequency of alternative polyadenylation (APA) in S2R+ cells (A) and adult male flies (B), as detected after pooling all data obtained at the different temperatures from the three biological replicates. In total, 14,669 PASs were detected in S2R+ cells, and 22,378 in adult male flies. These were assigned (see Materials and Methods) to a total of 7495 and 10,757 expressed genes in S2R+ cells and adult male flies, respectively. (C,D) Temperature effects on APA. For genes with multiple PASs, we compared the two PASs with highest presence (read counts) across all conditions and required the log2 fold change to be in opposite directions when comparing the read counts at the high (25 and 30 °C) with those at the low (11 and 14 °C) temperatures. The scatter plots display the fold changes at the proximal and distal of these two most strongly affected PASs in S2R+ cells (C) and adult flies (D). Blue dots represent genes (class I), where preference of the distal over the proximal PAS at low temperature is significant in comparison to the high temperature. Red dots represent genes (class II), where temperature change has a significant effect in opposite direction. [file 12864_2021_8057_MOESM9_ESM.pdf]

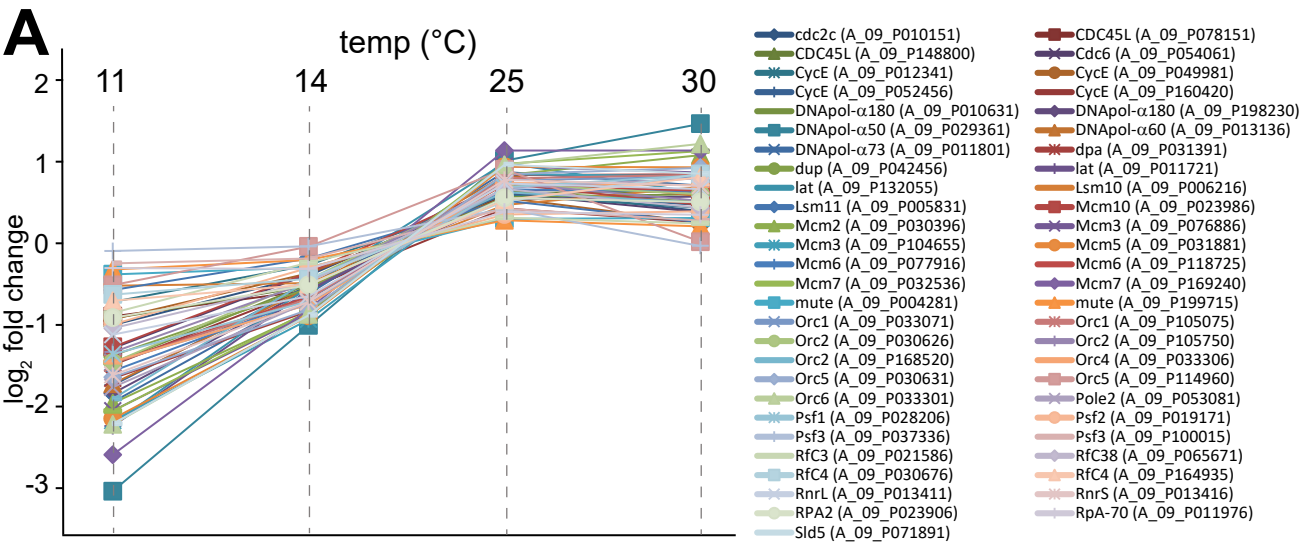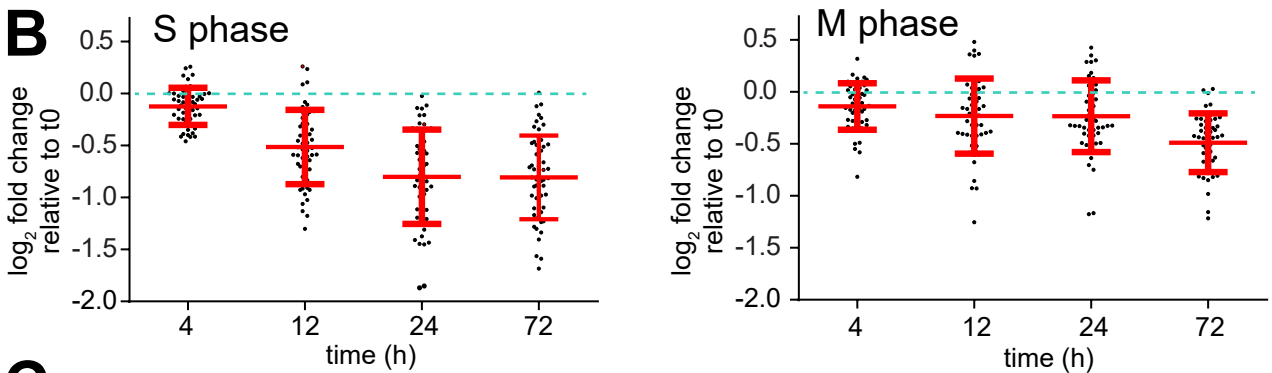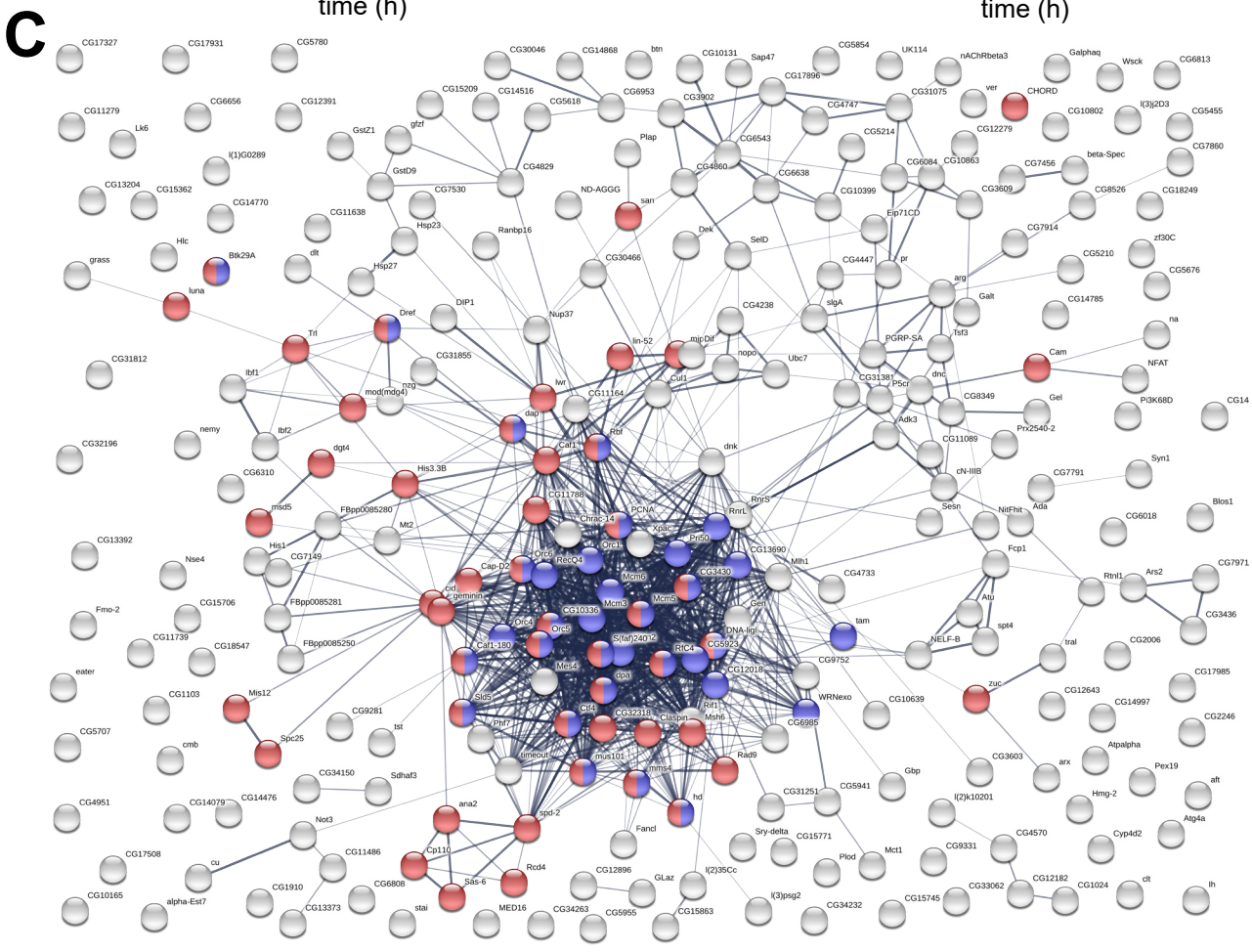

Supplement: Supplementary file 10 — Additional file 10: Fig. S4. Downregulation of cell cycle genes in S2R+ cells at low temperature. (A) Temperature dependence of transcript levels of S phase genes. The S2R+ cell transcriptome was analyzed after 24 h of incubation at different temperatures (11, 14, 25 and 30 °C) using microarrays. Temperature dependence of signal intensities (mean of three biological replicates) observed with microarray probes for transcripts derived from a curated set of genes crucial for progression through S phase is displayed after baseline transformation. (B) Temporal dynamics S- and M phase gene downregulation after a shift from 25 to 14 °C. Transcriptome changes were analyzed with microarrays at different times (0, 4, 12, 24 and 72 h) after the temperature downshift. Fold change relative to expression at t = 0 (dashed green line) of signal intensities (mean of three biological replicates) observed with microarray probes for transcripts derived from a curated set of genes crucial for progression through S- and M phase was calculated. Mean and s.d. are displayed in red. (C) Clustering of temperature-regulated genes according to their temporal expression dynamics after a 25- > 14 °C shift using k-means identified a prominent cluster of genes with persistent downregulation (Fig. 3E, cluster 2). Functional interactions among the genes in this cluster, as revealed by analysis with the STRING database, are displayed. Genes associated with the GO term “DNA replication” (GO:0006260), the term most strongly enriched (FDR = 4.15 × 10− 26) by the genes in this cluster, are marked in blue. Genes associated with “cell cycle” (GO:0007049, also strongly enriched, FDR = 3.72 × 10− 15) are marked in red. [file 12864_2021_8057_MOESM10_ESM.pdf]

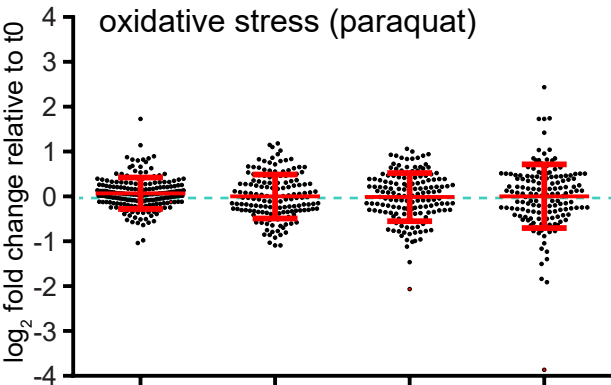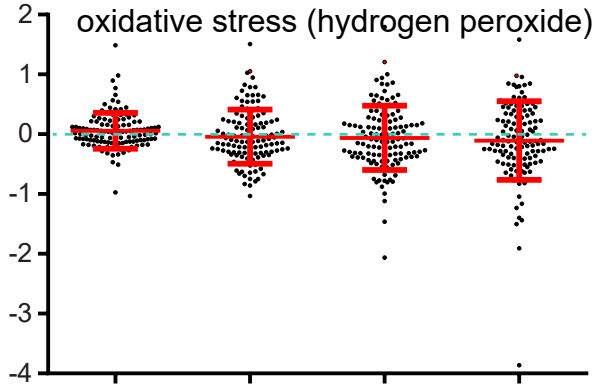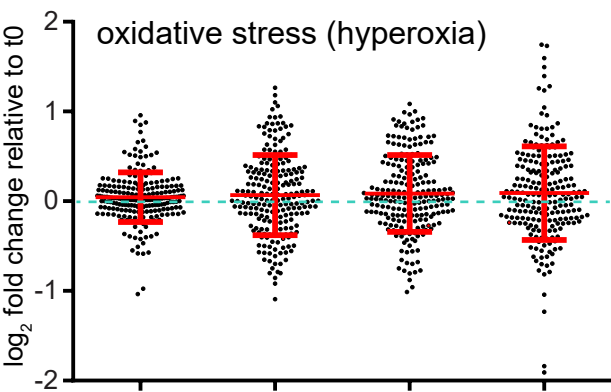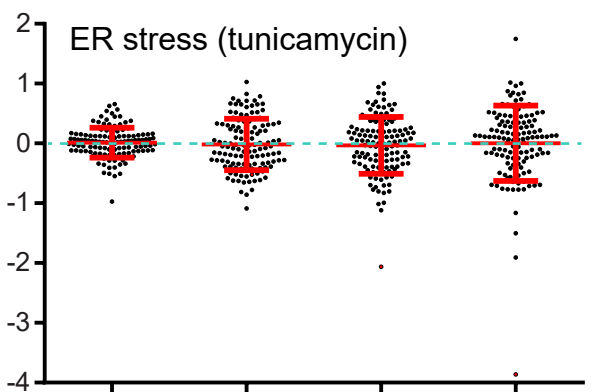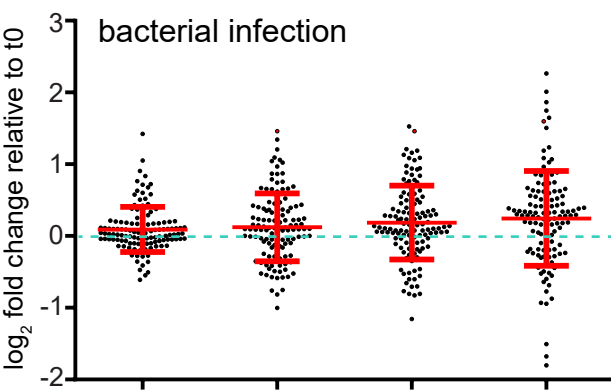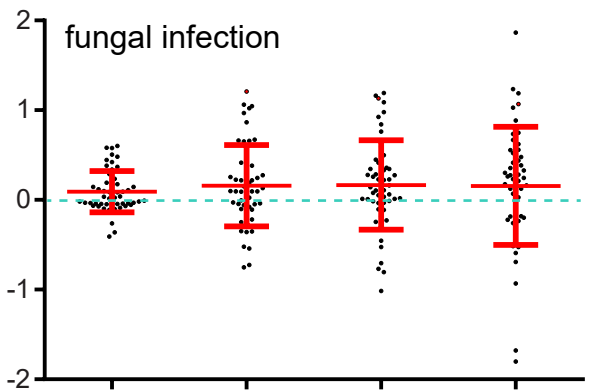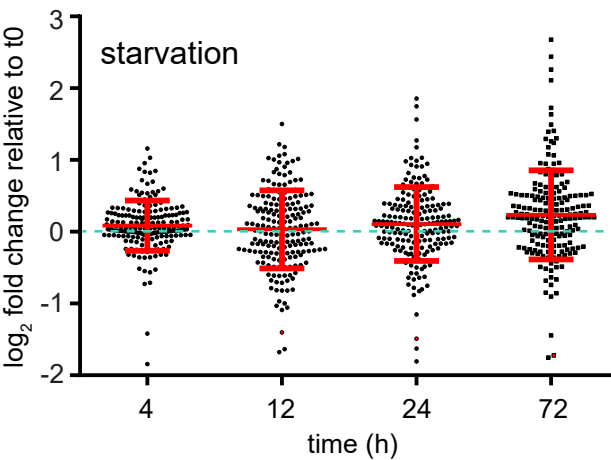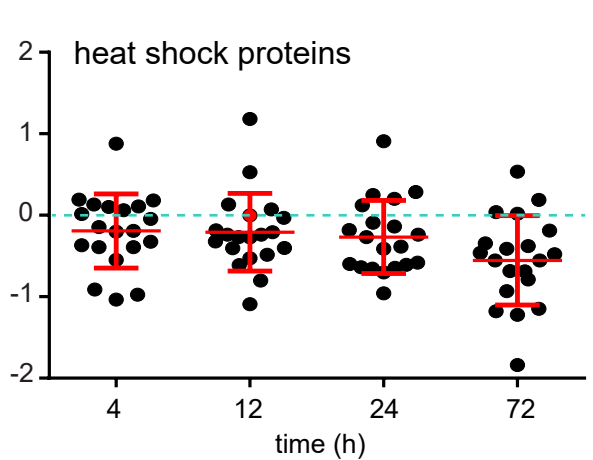

Supplement: Supplementary file 12 — Additional file 12: Fig. S5. Absence of stress response gene induction after temperature downshift to 14 °C. Microarray data from the time course analysis of S2R+ cell transcriptomes after a 25- > 14 °C temperature shift were used for an analysis of the response of known stress response genes. Probes detecting transcripts of genes previously reported to be induced by the indicated stressors were identified. Signals obtained at t0 were set to 1 and fold change at different times after temperature downshift was calculated. Swarm plots display log2 values of the fold changes, as well as means and s.d. in red. [file 12864_2021_8057_MOESM12_ESM.pdf]

**A**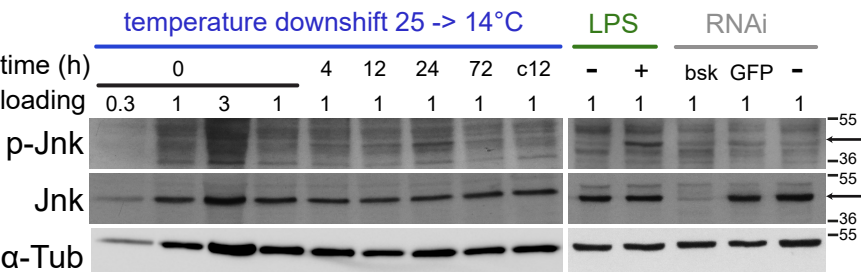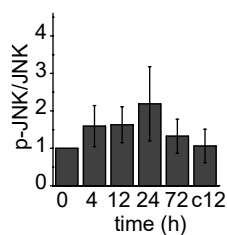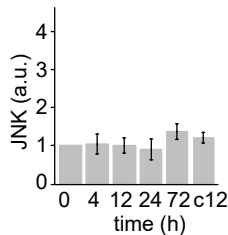**B**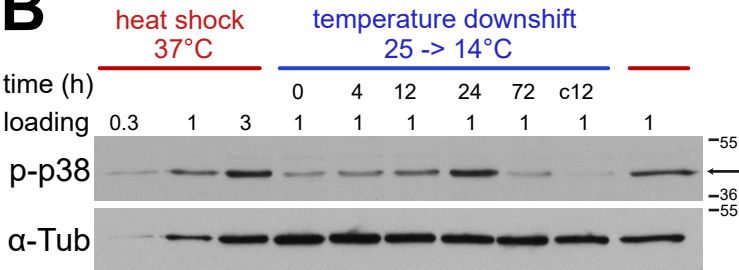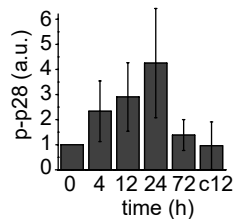

Supplement: Supplementary file 13 — Additional file 13: Fig. S6. Transient activation of JNK and p38 protein kinases after temperature downshift to 14 °C. (A,B) Immunoblotting with antibodies specific for the activated forms of JNK (phospho-JNK) and p38 (phospho-p38) were used for analysis. S2R+ cells were plated and grown at 25 °C before a shift to 14 °C at t = 0. Extracts were prepared at t = 0, 4, 12, 24, and 72 h after downshift, and also from an aliquot maintained for 12 h at 25 °C after t = 0. Relative amounts of extracts analyzed by immunoblotting are indicated. Arrows mark the bands corresponding to phospho-JNK, JNK and phospho-p38, and dashes the position of molecular weight markers. (A) For control of antibody specificities, we also analyzed extracts from S2R+ cells treated with lipopolysaccharide (LPS) or depleted of bsk transcripts (coding for JNK) or GFP transcripts (for control) by RNAi. Immunoblots were probed with anti-phospho-JNK, anti-JNK and anti-α-tubulin. Signal intensities in the bands representing JNK and phospho-JNK were quantified in four replicates. A bar diagram displays average intensities (+/− s.d.) normalized to those at t = 0. (B) For comparison, we also analyzed extracts from S2R+ cells after exposure (45 min) to a heat shock at 37 °C. Relative amounts of extracts analyzed by immunoblotting are indicated. Immunoblots were probed with anti-phospho-p38 and anti-α-tubulin. The arrow indicates the band corresponding to phospho-p38. Position of molecular weight markers are indicated. Signal intensities in the bands representing phospho-p38 were quantified in three replicates. A bar diagram displays average intensities (+/− s.d.) normalized to those at t = 0. [file 12864_2021_8057_MOESM13_ESM.pdf]

**A**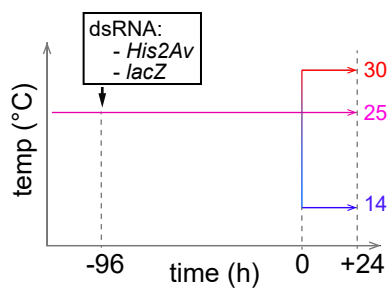**B**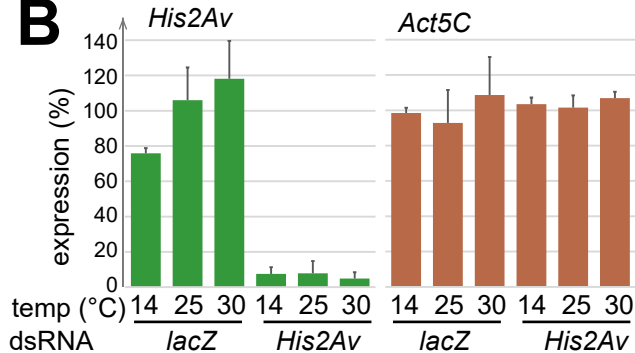**C**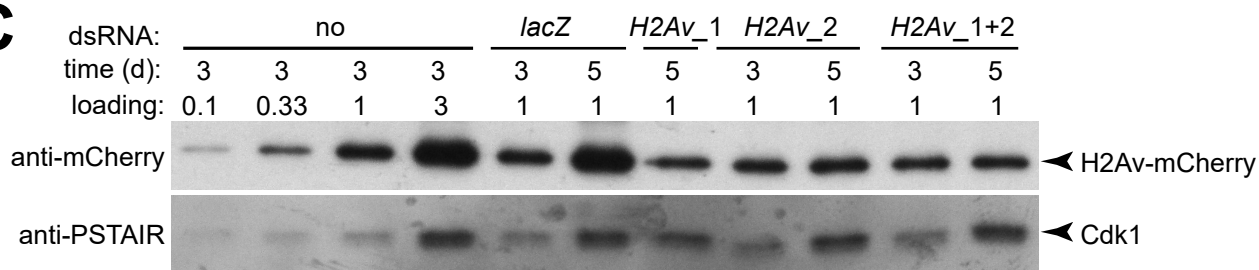**D**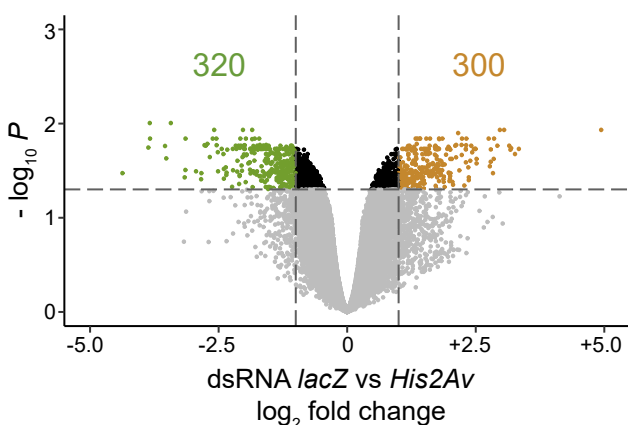**E**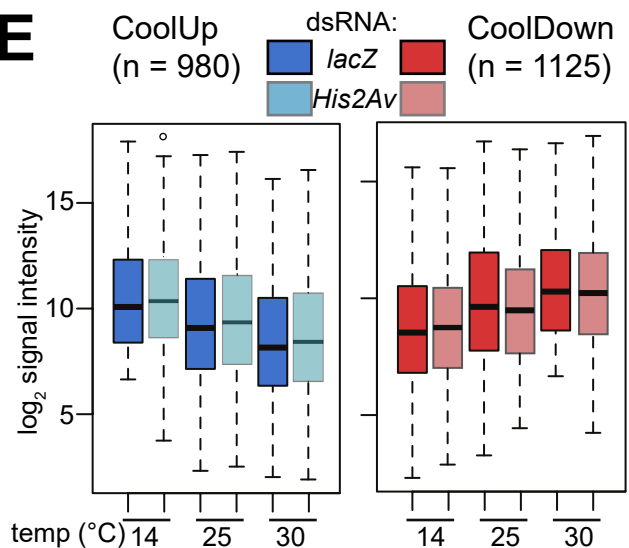**F**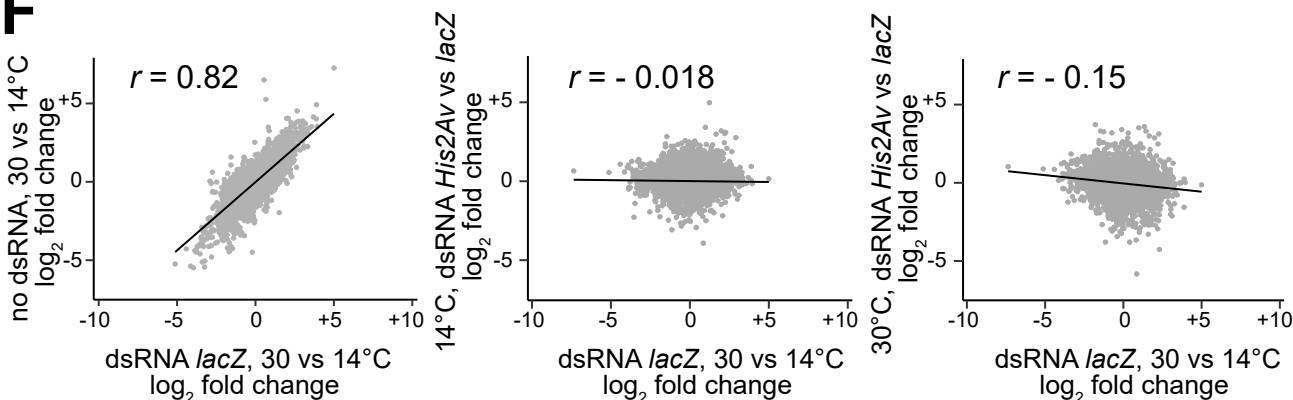

Supplement: Supplementary file 14 — Additional file 14: Fig. S7. Evaluation of the role of histone H2Av (His2Av) in transcriptional control of temperature-regulated genes in S2R+ cells. (A) To address the role of His2Av in temperature-dependent regulation of gene expression in S2R+ cells, we added His2Av dsRNA or lacZ dsRNA for control to culture aliquots followed by a shift to the indicated temperatures after four days. Twenty-four hours after the shift, we isolated total RNA for analysis with microarrays. Three replicate experiments were performed. (B) Analysis of transcript levels of His2Av and Act5C (for control) confirmed successful depletion of His2Av transcripts. Bar diagrams display transcript levels (average of three biological replicates and s.d.) at the analyzed temperatures. Average expression at the three temperatures observed after treatment with lacZ dsRNA was set to 100%. (C) Analysis of His2Av depletion by immunoblotting. S2R + _MtnAp-His2Av-mRFP cells (Lidsky et al. 2013) were treated with dsRNA derived from lacZ (for control) and two distinct His2Av amplicons (H2Av_1 and H2Av_2) for three and 5 days as indicated. Cell extracts were analyzed by immunoblotting with anti-mCherry (detecting His2Av-mRFP) and anti-PSTAIR (loading control). The combined treatment with both His2Av dsRNA preparations (H2Av_1 + 2) for 5 days was found to reduce His2Av-mRFP levels to 40% of controls according to quantification of signal intensities and normalization based on anti-PSTAIR signals. (D) Effects of His2Av depletion on the S2R+ cell transcriptome. A volcano plot is displayed with probes associated with signal intensities that were significantly (FDR < 0.05; fold change ≥2) down- (green dots, 320 probes) or upregulated (brown dots, 300 probes) by His2Av depletion in comparison to control (lacZ dsRNA treatment). The comparison based on signals detected at 14 °C is shown. Analogous comparisons at 25 or 30 °C resulted in comparable observations. (E) Effects of His2Av depletion on transcript levels of te [file 12864_2021_8057_MOESM14_ESM.pdf]

**A**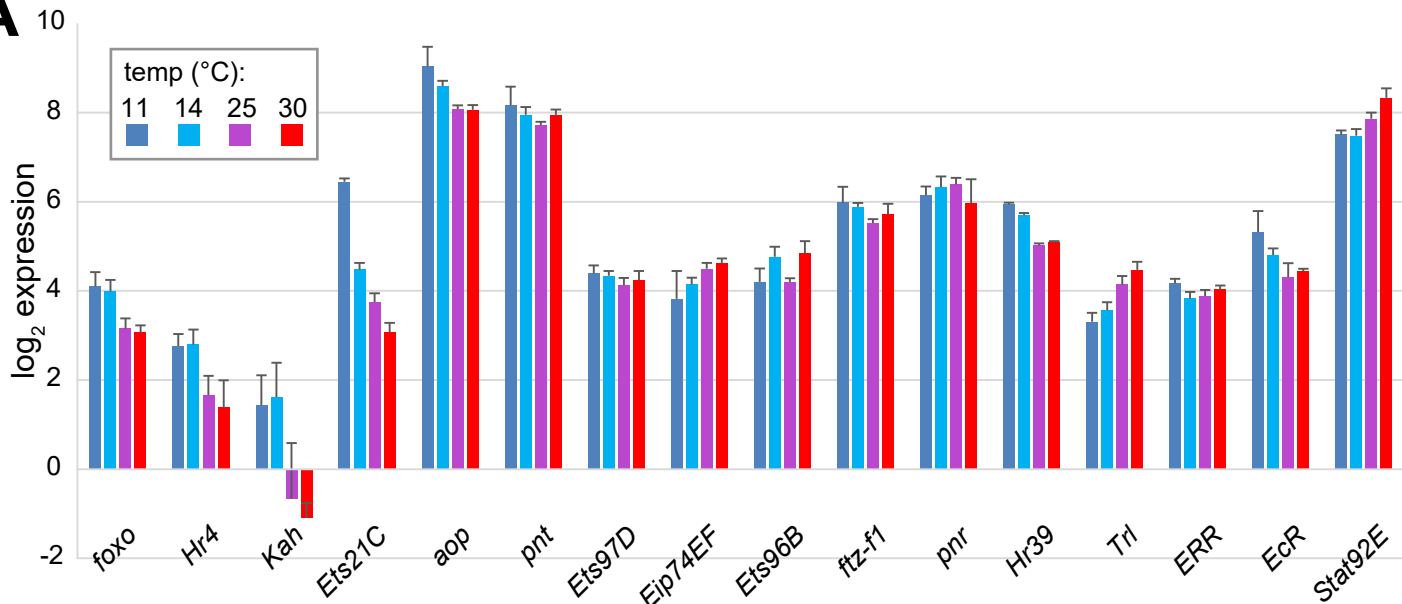**B**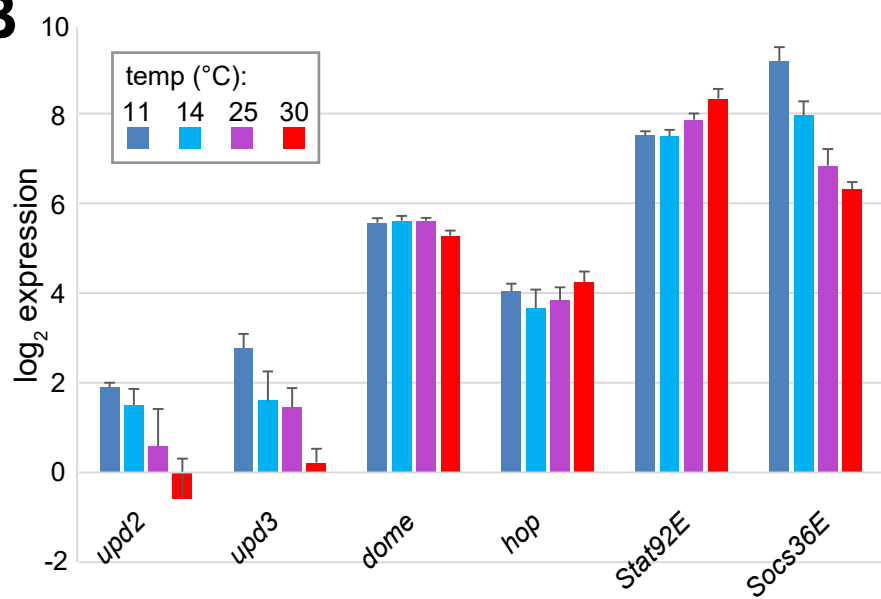**C**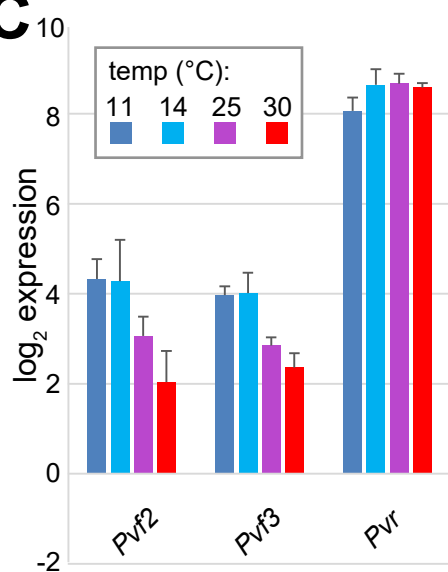**D**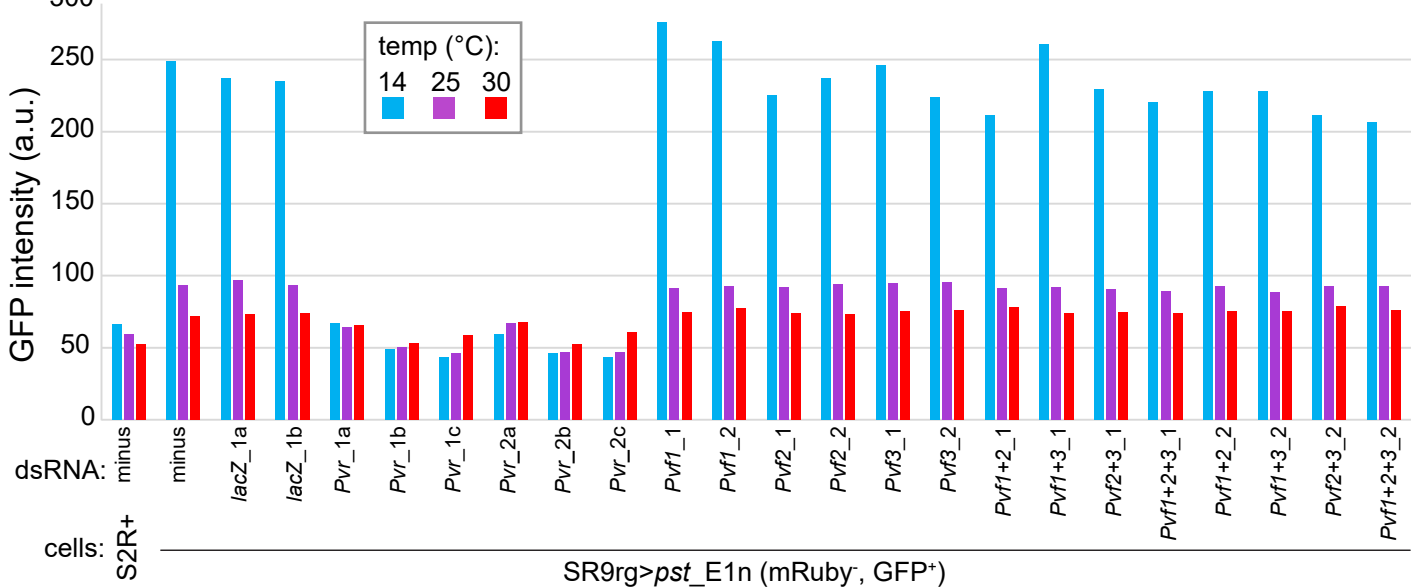

Supplement: Supplementary file 20 — Additional file 20: Fig. S11. Temperature dependence of transcript levels of candidate regulators of pst_E1n enhancer activity and dependence on Pvf/Pvr. (A-C) Transcript levels at the indicated temperatures in S2R+ cells as detected by 3′ RNA-Seq. Bar diagrams display log2 of the average of the read counts (after normalization) and s.d. (n = 3 replicate experiments). (A) Transcript levels of TFs with predicted binding sites within pst_E1n and detectable expression in S2R+ cells. (B) Transcript levels of JAK/STAT signal transduction proteins. Expression of upd1 is marginal at most in S2R+ cells and hence not included. (C) Transcript levels of the genes encoding the receptor tyrosine kinase Pvr and its known ligands Pvf2 and Pvf3. Expression of Pvf1 is marginal at most in S2R+ cells and hence not included. (D) Dependence of pst_E1n enhancer activity on Pvfs and Pvr. The indicated dsRNAs were used for depletion in SR9rg > pst_E1n (mRuby−, GFP+) cells, which were then shifted in aliquots to the indicated temperatures and analyzed eventually by flow cytometry. Bars display median GFP signal intensity. For depletion of Pvr and Pvfs, two dsRNA preparations (1 or 2) generated from distinct amplicons, were used in multiple experiments (indicated by a, b or c) in some cases. [file 12864_2021_8057_MOESM20_ESM.pdf]

**A**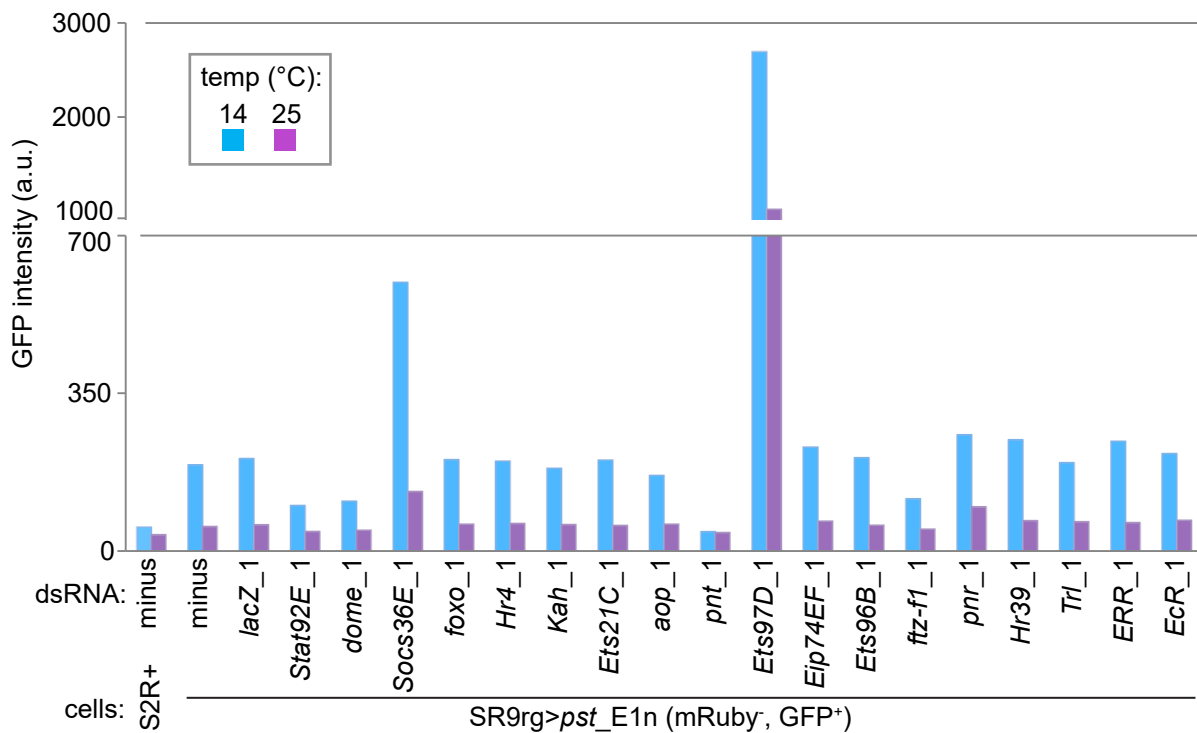**B**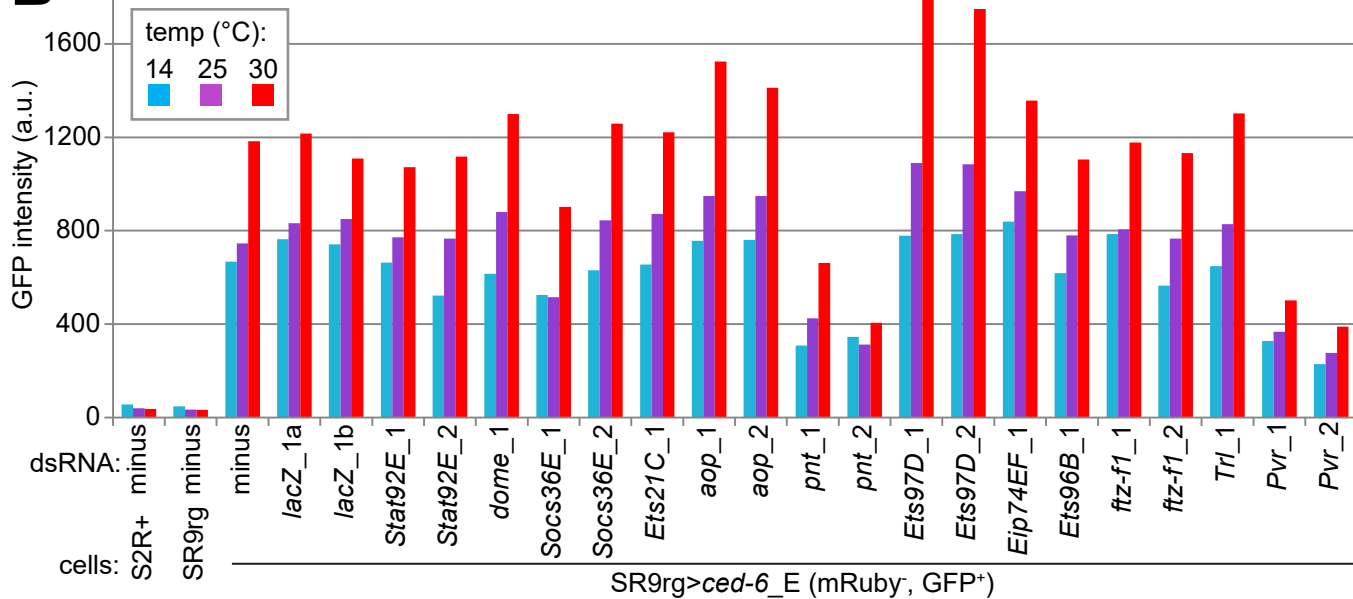

Supplement: Supplementary file 21 — Additional file 21: Fig. S12. Enhancer activity after depletion of TFs, JAK/STAT and Pvr. (A,B) The indicated dsRNAs were used for depletion of TFs with predicted binding sites in pst_E1n. In addition, JAK/STAT signaling pathway proteins (Dome and Socs36E) and the receptor tyrosine kinase Pvr were depleted. In some cases, two dsRNA preparations (1 or 2) generated from distinct amplicons were used. lacZ ds RNA was used in two replicates (a and b). Depletions were performed in SR9rg > pst_E1n (mRuby−, GFP+) cells (A) and in SR9rg > ced-6_E (mRuby−, GFP+) cells (B). Depleted cells and control cell lines (S2R+ and SR9rg) were then shifted in aliquots to the indicated temperatures and analyzed eventually by flow cytometry. Bars display median GFP signal intensity. [file 12864_2021_8057_MOESM21_ESM.pdf]

**A**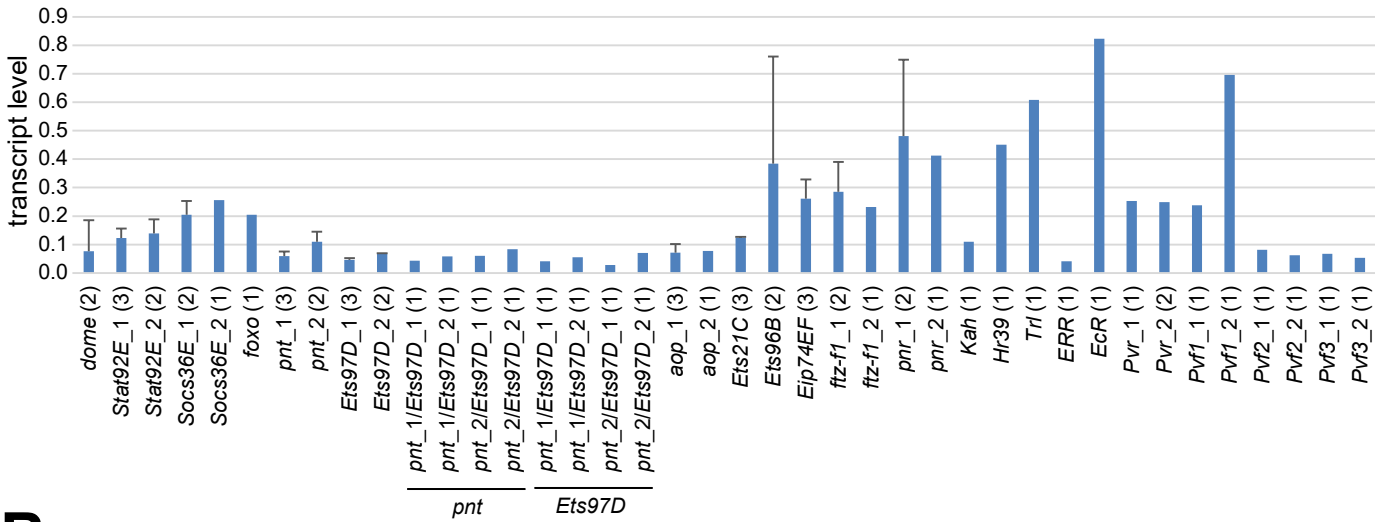**B**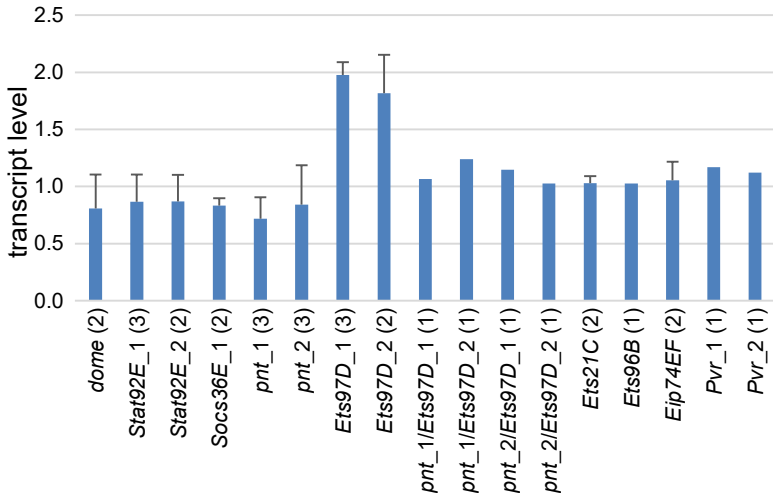

Supplement: Supplementary file 22 — Additional file 22: Fig. S13. Depletion efficiency and effect on endogenous pst transcript levels. (A,B) The indicated dsRNAs were used for RNAi with SR9rg > pst_E1n (mRuby−, GFP+) cells. Two dsRNA preparations (1 or 2) generated from distinct amplicons were used for some targets. The number of independent experiments is indicated in brackets. In addition, lacZ dsRNA was used in parallel for control. Bars represent average transcript levels as determined by qRT-PCR relative to those detected after lacZ depletion, which were set to 1. Whiskers indicate s.d. in case of multiple independent experiments. (A) To assess RNAi efficiency, total RNA was isolated after four days of depletion at 25 °C followed by analysis of the target transcript levels. The target analyzed in case of double depletion is indicated at the bottom. (B) The levels of transcripts derived from the endogenous pst gene were analyzed. [file 12864_2021_8057_MOESM22_ESM.pdf]

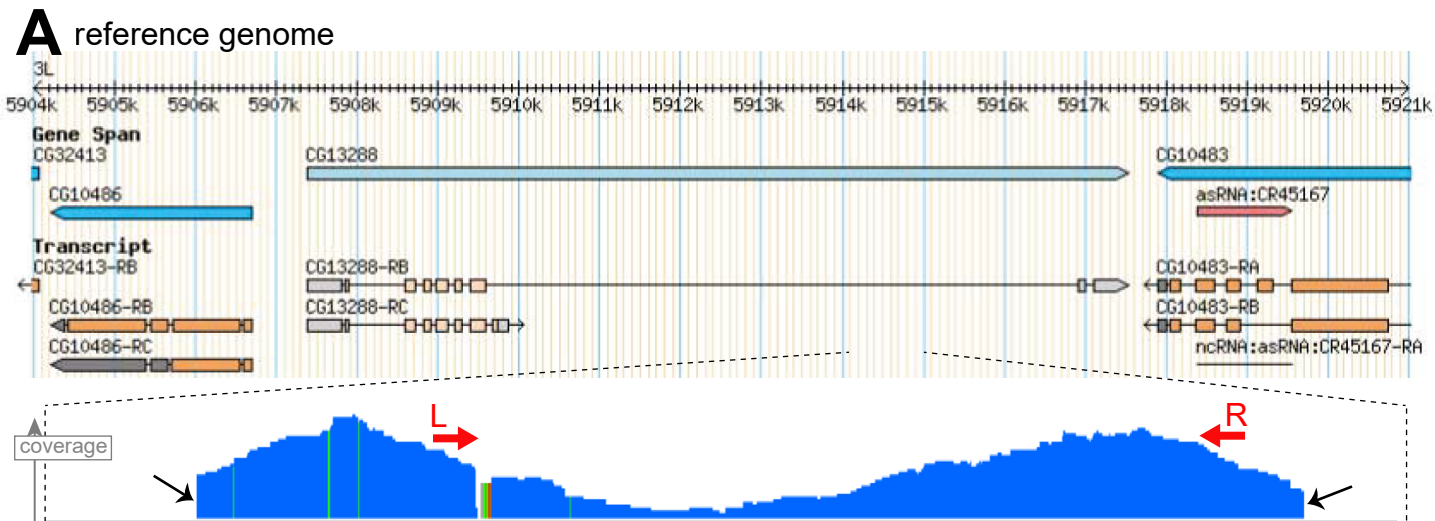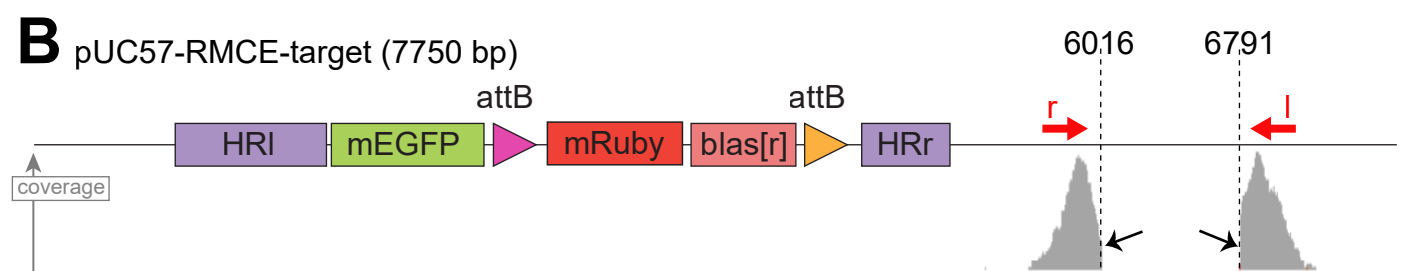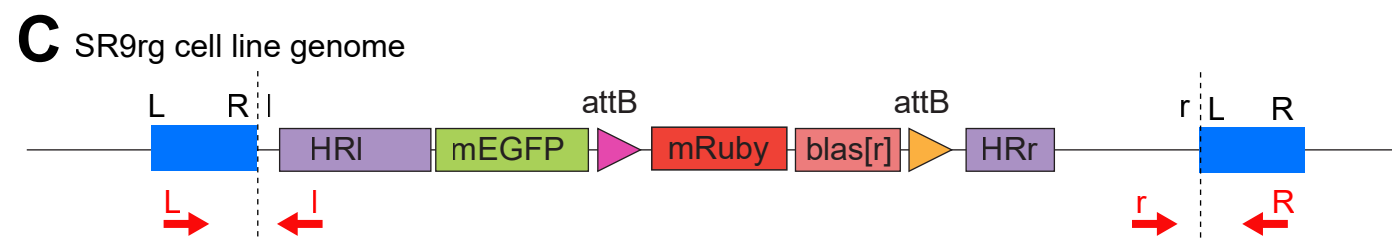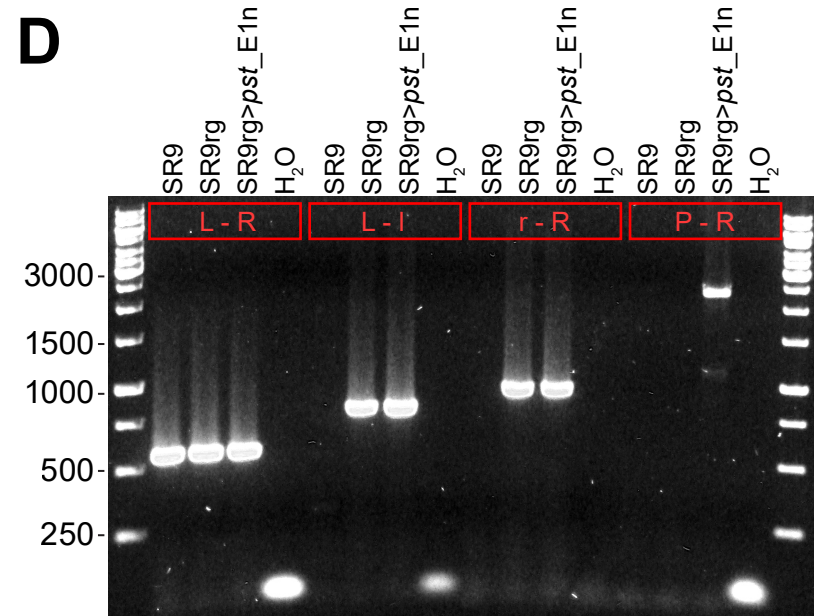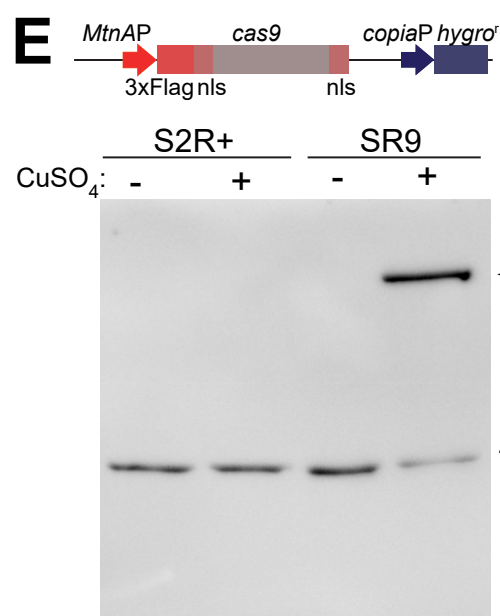

Supplement: Supplementary file 24 — Additional file 24: Fig. S14. Characterization of SR9 and SR9rg cells. (A-D) Identification of a functional target locus for RMCE in SR9rg cells. Whole genome sequencing was used for the characterization of the clonal SR9rg cell line. Hybrid paired reads, where one read aligns to genomic sequences and the mate read to pUC57-RMCE-target plasmid sequences were identified and used for mapping chromosomal insertions of pUC57-RMCE-target. An insertion that is competent for RMCE was identified on chromosome 3 L in an intron of the CG13288 gene. (A) The genomic region (dmel_r6.19, chr3L:5904000 to chr3L:5921000) with CG13288 as visualized by GBrowse 2.0 is displayed on top. The cumulative base coverage by hybrid paired reads mapping to this region is indicated by the blue bars below (from 0 to 55 reads/bp). Black arrows emphasize the distal clustering of the abrupt end of the alignments on either side, corresponding to the genome-plasmid junctions. Primers “L” and “R” (red arrows) with sequences close to the left and right ends of this genomic region were used for confirmation of the insertion by PCR (see panel D). (B) A linearized representation of pUC57-RMCE-target is shown on top. The grey bars below display the cumulative base coverage (from 0 to 51 reads/bp) by the hybrid paired reads mapping to the CG132888 region. Black arrows and dashed lines indicate breakpoints (abrupt clustered ends of read alignments). Primers “r” and “l” (red arrows) with sequences close to the right and left ends of the plasmid region were used for confirmation of the insertion by PCR (see panel D). (C) Proposed organization of pUC57-RMCE-target insertion in the CG13288 region. The plasmid pUC57-RMCE-target is inserted almost completely, starting from breakpoint “l” and extending to the breakpoint “r” (see panel B). The inserted plasmid region is flanked by a tandem duplication of the genome region between “L” and “R” (see panel A). Primers used for confirmation of the proposed organization a [file 12864_2021_8057_MOESM24_ESM.pdf]
