# Supplementary material for: A cis-regulatory element promoting increased transcription at low temperature in cultured ectothermic Drosophila cells
Source: BMC Genomics. 2021 Oct 28;22:771. doi: 10.1186/s12864-021-08057-4 (PMC8555087; doi:10.1186/s12864-021-08057-4)
Supplement: Supplementary file 17 — Additional file 17: Fig. S8. DNA accessibility in nuclear chromatin of S2R+ cells. For illustration of ATAC-Seq data quality, various analyses based on one of the samples (replicate of cells tagmented after growth at 25 °C) are displayed. (A) Distribution of ATAC-Seq fragment lengths. Arrows indicate periodicity reflecting nucleosomal organization. (B) Enrichment of ATAC-Seq reads around annotated transcription start sites (TSS). Distance (base pairs [bp]) from the transcriptional start site (TSS) is indicated on the x axis. (C) Distribution of ATAC-Seq reads across annotated genome features. [file 12864_2021_8057_MOESM17_ESM.pdf]

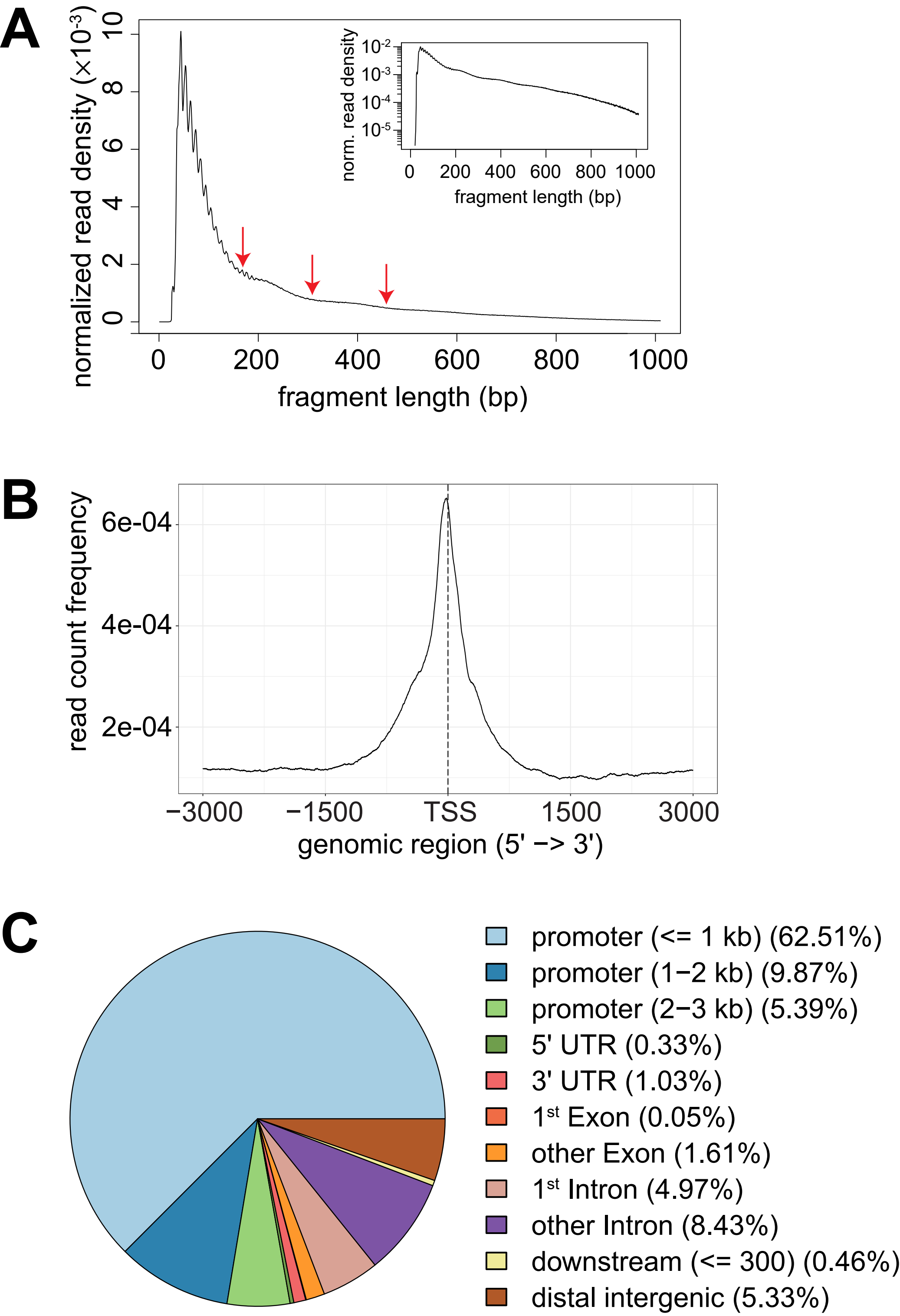
A
normalized read density (×10^-3)
0
2
4
6
8
10
0
200
400
600
800
1000
fragment length (bp)
norm. read density
10^-2
10^-3
10^-4
10^-5
0
200
400
600
800
1000
fragment length (bp)
B
read count frequency
2e-04
4e-04
6e-04
−3000
−1500
TSS
1500
3000
genomic region (5' −> 3')
C
promoter (<= 1 kb) (62.51%)
promoter (1−2 kb) (9.87%)
promoter (2−3 kb) (5.39%)
5' UTR (0.33%)
3' UTR (1.03%)
1st Exon (0.05%)
other Exon (1.61%)
1st Intron (4.97%)
other Intron (8.43%)
downstream (<= 300) (0.46%)
distal intergenic (5.33%)
