# Supplementary material for: A cis-regulatory element promoting increased transcription at low temperature in cultured ectothermic Drosophila cells
Source: BMC Genomics. 2021 Oct 28;22:771. doi: 10.1186/s12864-021-08057-4 (PMC8555087; doi:10.1186/s12864-021-08057-4)
Supplement: Supplementary file 18 — Additional file 18: Fig. S9. Assay for temperature-dependent enhancer activity with candidate CREs. (A) Table summarizing location and activity of candidate CREs analyzed after RMCE with SR9rg cells. (B) Data for the analyzed candidate CREs. Candidate CREs (designation in bold on the left side) were selected based on data obtained by ATAC-Seq (top three browser tracks) and 3′ RNA-Seq (bottom three browser tracks). Grey shading represents the region of the analyzed DNA fragment. Green arrowheads indicate the direction of transcription of the proximal gene. Scatter plots on the right side present green (x axis) and red (y axis) fluorescence intensities as determined by flow cytometry with SR9rg cells after RMCE and incubation at the indicated temperatures. [file 12864_2021_8057_MOESM18_ESM.pdf]

A

name	transcript level	test fragment[a)]	promoter[b)]	enhancer activity	temperature dependence [c)]
*Prx2540-1*_E	WarmUp	chr2R:10423096-10424598	-	++	WarmUp
*Mcm2*_E	WarmUp	chr3R:8264489-8264947	+	(+)	
*CG31075*_E_inverted	WarmUp	chr3R:26988420-26989034	+	-	
*CG13694*_E	CoolUp	chr2L:116969-116021	-	-	
*mthl14*_E	CoolUp	chr3L:245757-248102	-	+	
*CG13893*_E		chr3L:604500-600889	-	+	
*CG6231*_E1	CoolUp	chr3R:19622194-19619140	-	++	WarmUp
*CG6231*_E2	CoolUp	chr3R:19630067-19627647	+	-	
	CoolUp		-	-	
*CG6231*_E3	CoolUp	chr3R:19633419-19632370	+	-	
	CoolUp		-	-	
*Kah*_E1	CoolUp	chr3L:590671-587637	+	-	
*Kah*_E2	CoolUp	chr3L:589326-588826	+	(+)	
*Orct2*_E	CoolUp	chr3R:24271000-24273602	+	-	
*Lip4*_E	CoolUp	chr2L:10527000-10529600	+	(+)	
	CoolUp		-	-	

*Lip4*_E_inverted	CoolUp	chr2L:10529600-10527000	+	-	
*cv-2*_E1	CoolUp	chr2R:21369718-21367280	+	+	(CoolUp)
*cv-2*_E2	CoolUp	chr2R:21383738-21378813	+	+++	
*Pask*_E	CoolUp	chr2R:23987549-23989100	-	+	CoolUp
*pirk*_E	CoolUp	chr2R:21663200-21661544	-	-	
*mthl5*_E	CoolUp	chr3R:11888215-11886762	-	+++	(WarmUp)
*betaTub97E*F_E1_inverted	CoolUp	chr3R:27986500-27990003	+	-	
*betaTub97EF*_E2	CoolUp	chr3R:27990000-27988130	-	-	
*CR45607/upd2*_E	CoolUp	chrX:18246571-18250087	+	-	
*Socs36E*_E1	CoolUp	chr2L:18146300-18142749	+	-	
*Socs36E*_E2	CoolUp	chr2L:18142600-18141068	+	-	
*GILT2*_E	(CoolUp)	chr3R:23678729-23679439	+	-	
	(CoolUp)		-	(+)	
*Atf3*_E		chrX:1249873-1248024	+	(+)	
*CG11403*_E		chrX:1243130-1239626	+	-	
*CG32809*_E		chrX:1692228-1691362	+	-	
*ACC*_E		chr2R:7992450-7991766	+	(+)	
			-	-	
*pain*_E		chr2R:24920931-24920217	+	+	
*Rho1*_E		chr2R:16106015-16106899	+	+	
*CG43149*_E		chr3L:56221-55198	+	+	

*CG6888*_E		chr3L:15205814-15207085	+	(+)	
*CG14810*_E		chrX:1821507-1820886	+	-	
*CG15482*_E		chr2L:13211885-13211259	+	-	
*CR45693*_E		chr2L:14516828-14514926	+	+	
*CR43263*_E		chr2L:1218362-1217849	+	+	
*RNaseX25*_E1		chr3L:7969603-7970384	+	+++	CoolUp
*RNaseX25*_E2		chr3L:7968618-7970699	+	++	CoolUp
			-	+	
*RNaseX25*_E3		chr3L:7968618-7970384	+	++	CoolUp
*RNaseX25*_E4		chr3L:7969603-7970699	+	++	
			-	++	

a) chromosomal coordinates of the tested DNA fragments according to genome release dm6.
b) some candidate fragments, which included an endogenous promoter region, were inserted into an exchange plasmid lacking the DSCP. Accordingly, the presence (+) or absence (-) of the DSCP in the exchange plasmid is indicated.
c) the effect of temperature on enhancer activity is specified.

B

Bai et al., S9 Fig.

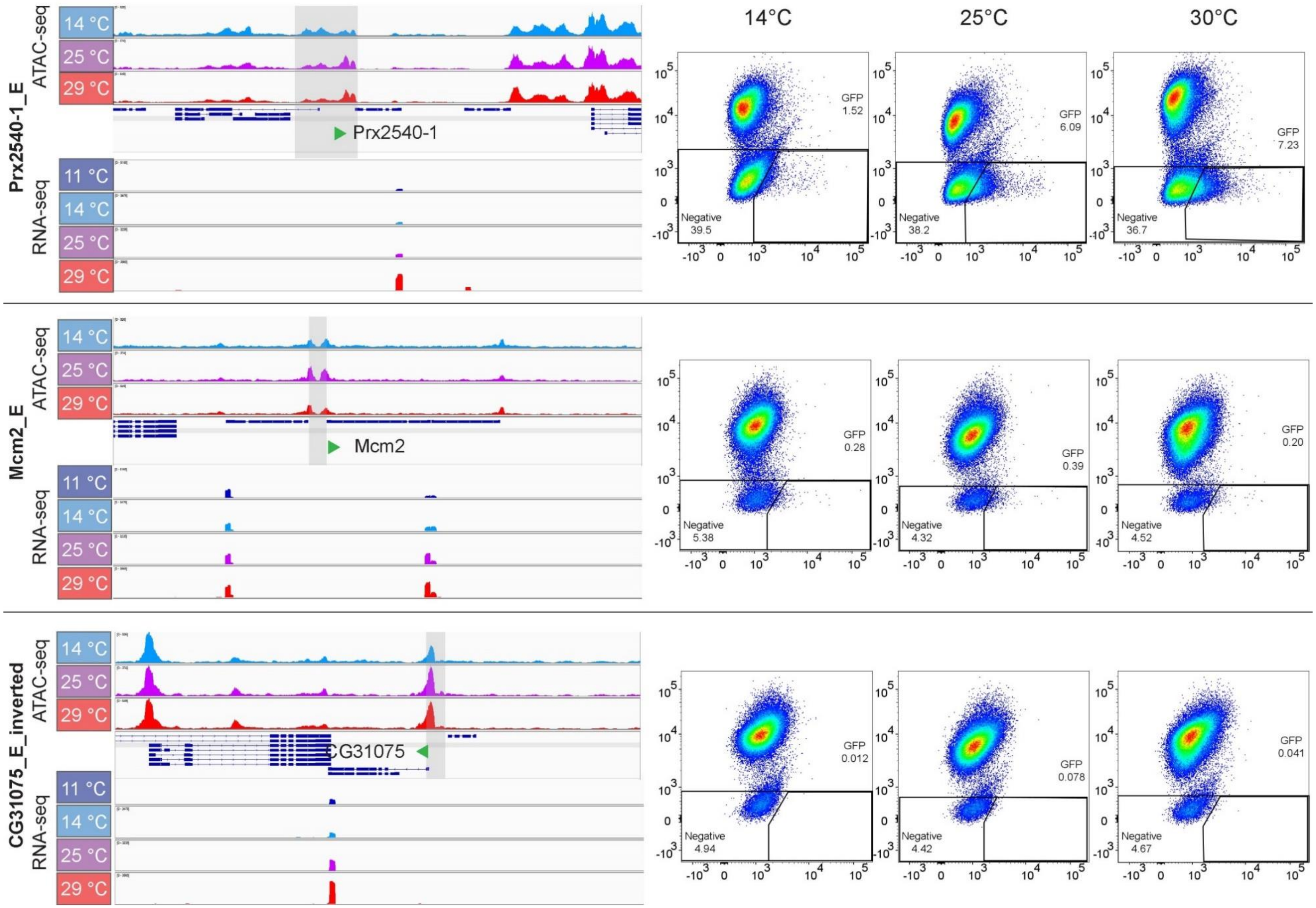

Bai et al., S9 Fig.

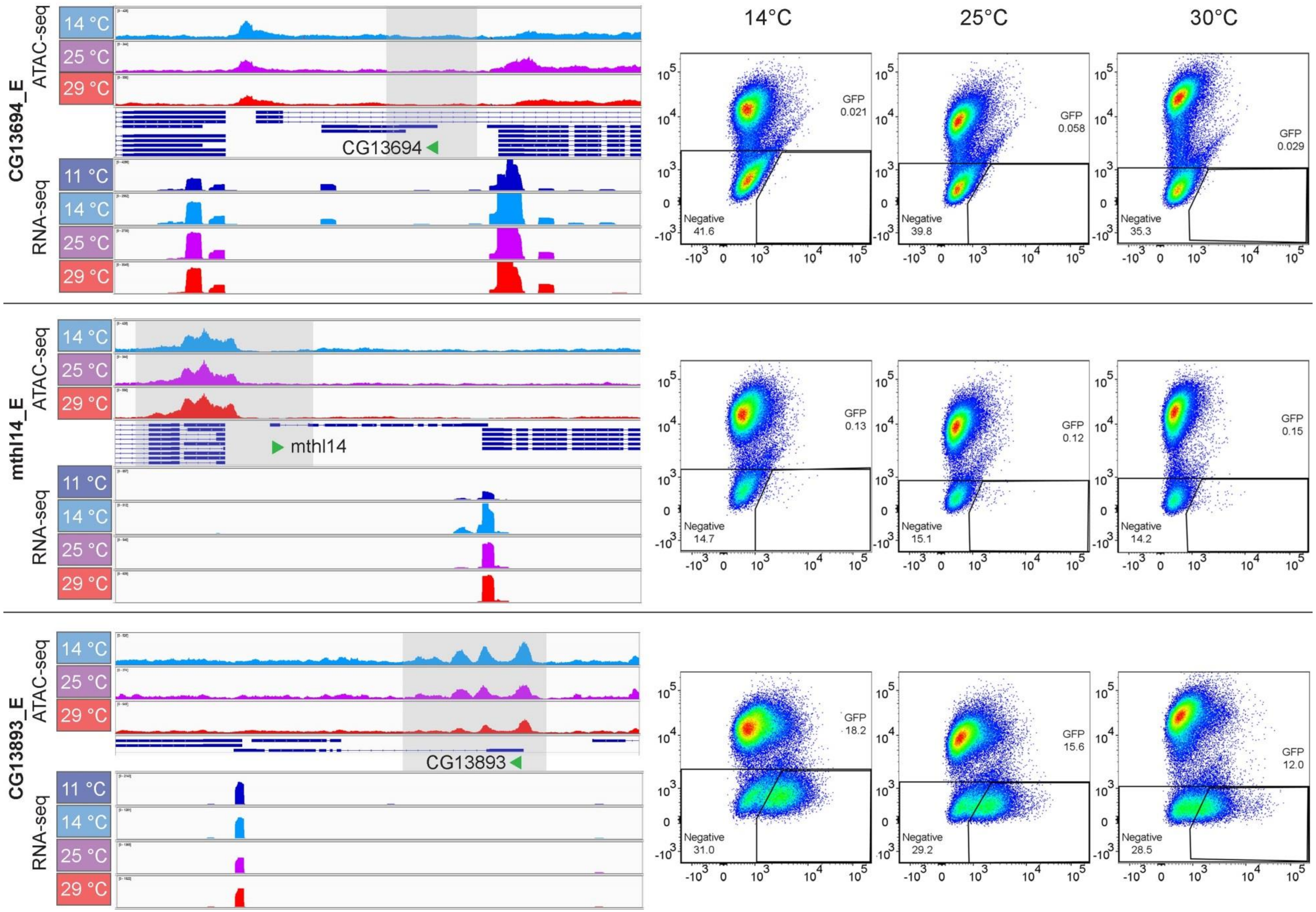

Bai et al., S9 Fig.

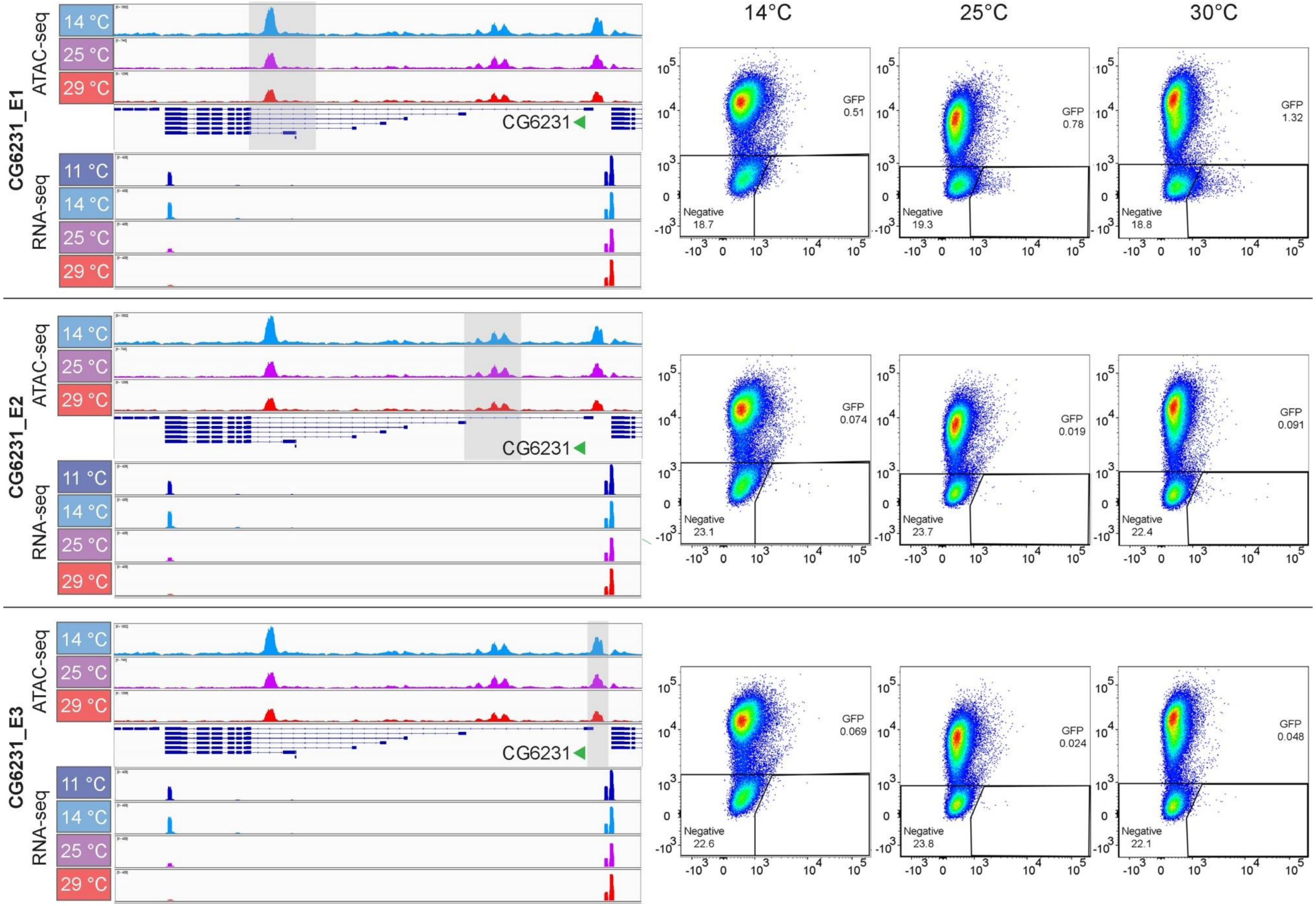

Bai et al., S9 Fig.

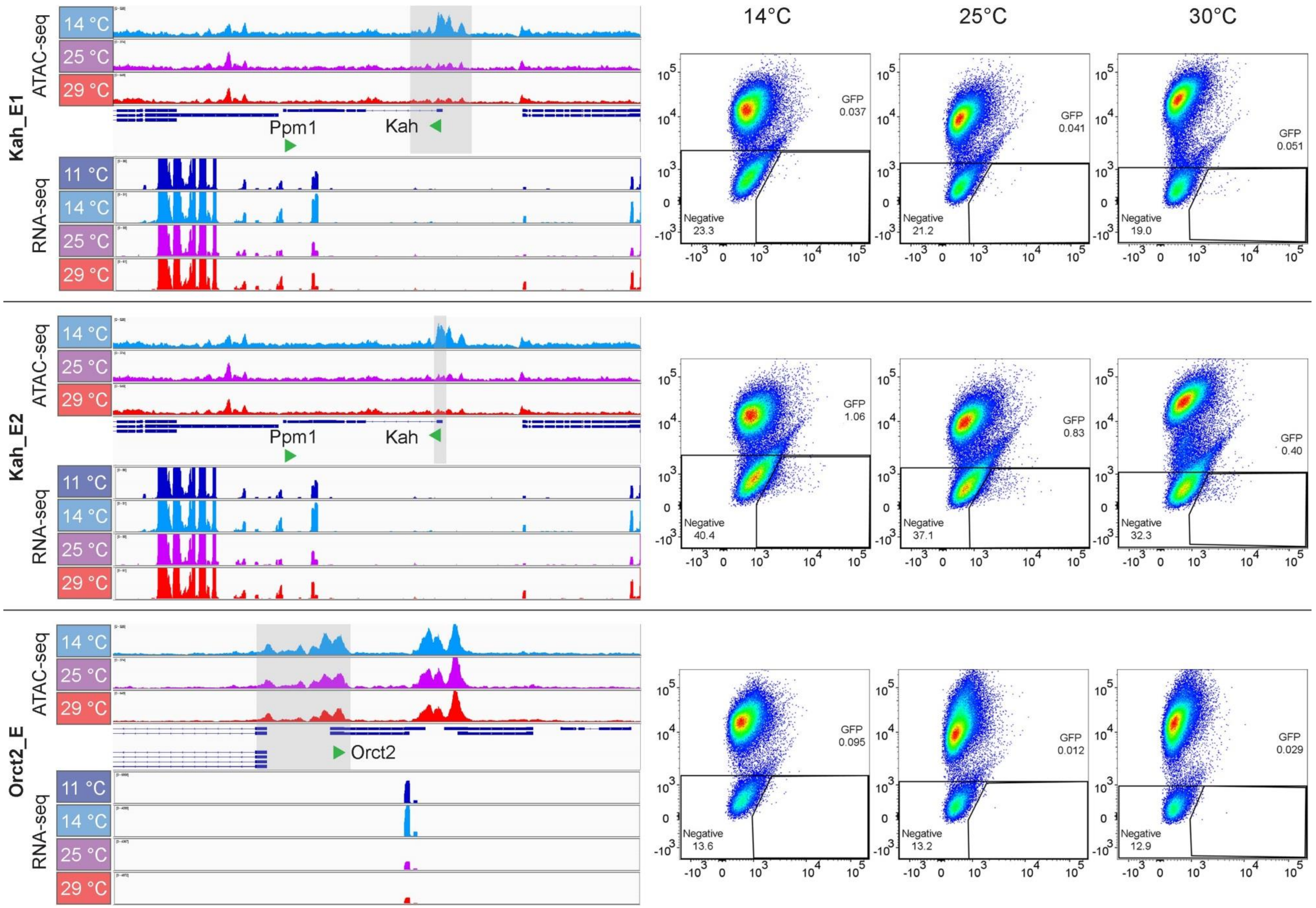

Bai et al., S9 Fig.

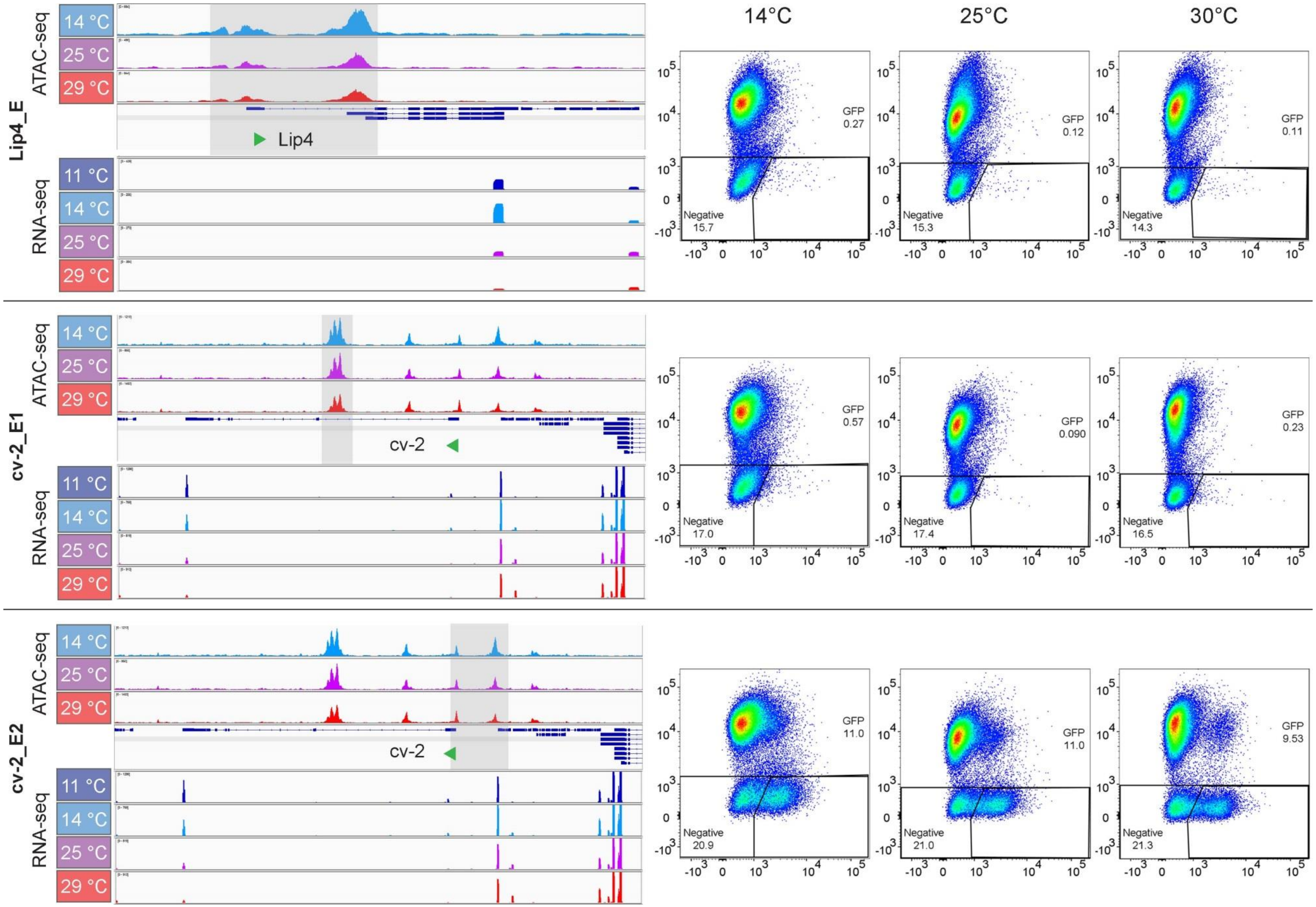

Bai et al., S9 Fig.

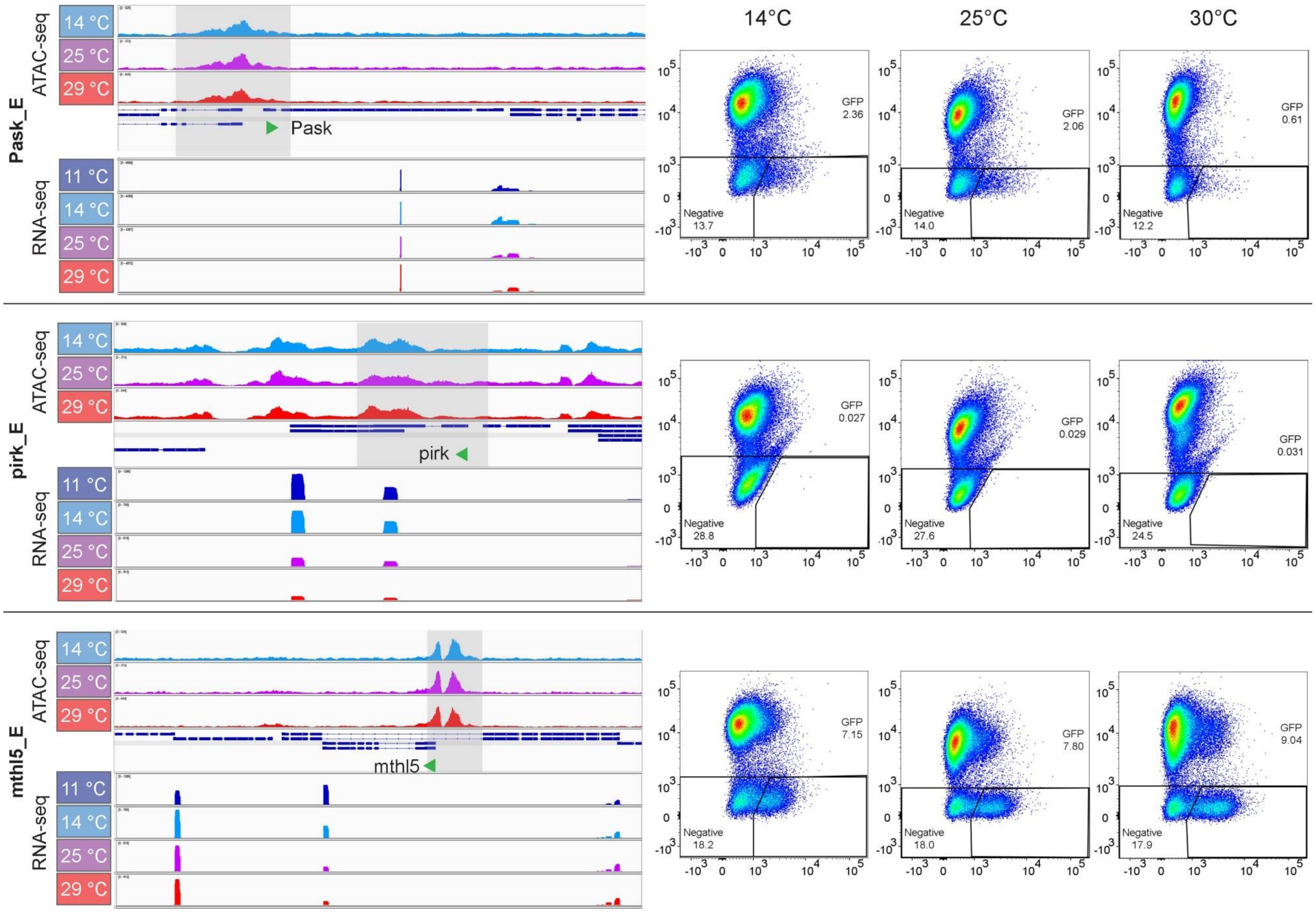

Bai et al., S9 Fig.

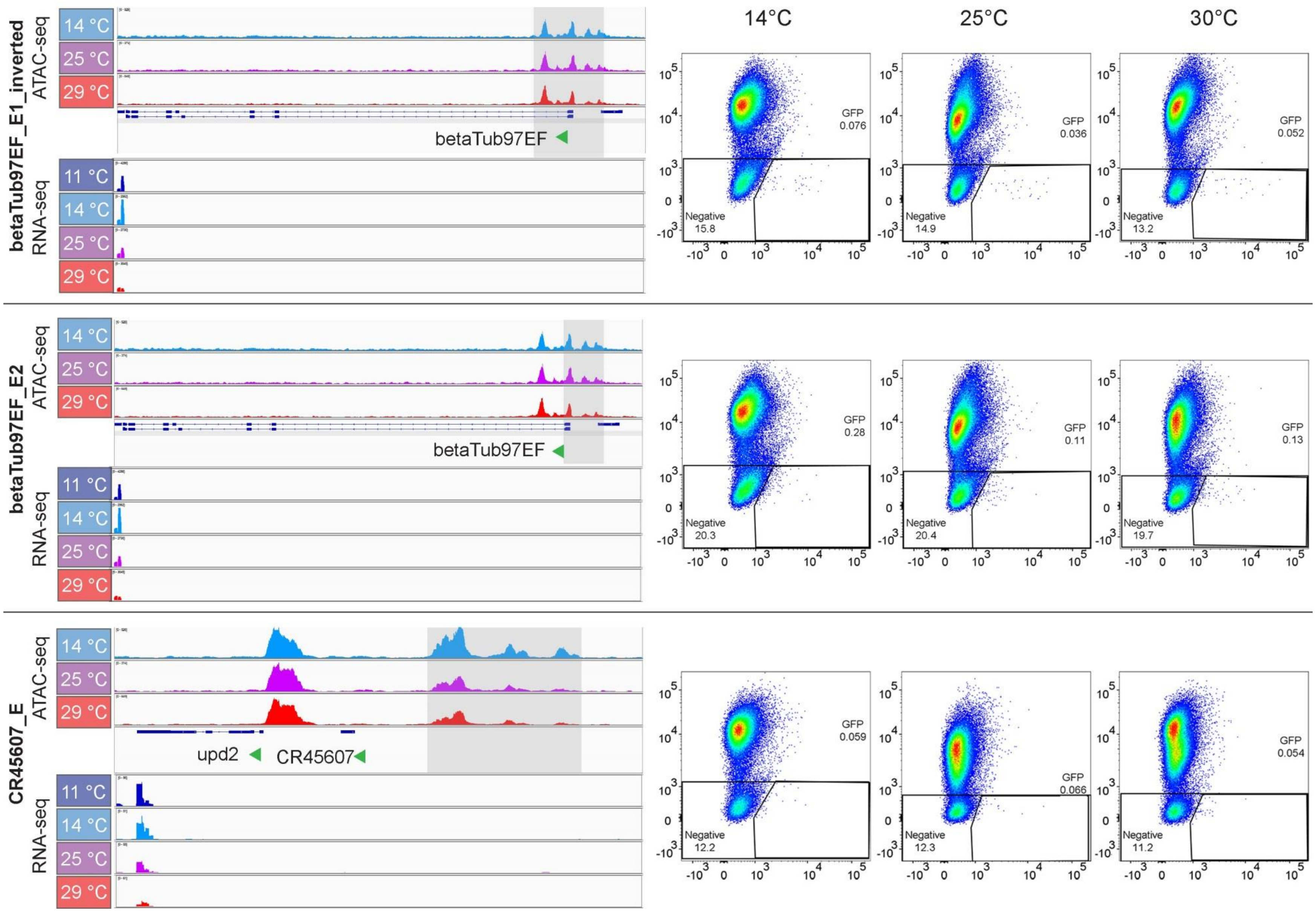

Bai et al., S9 Fig.

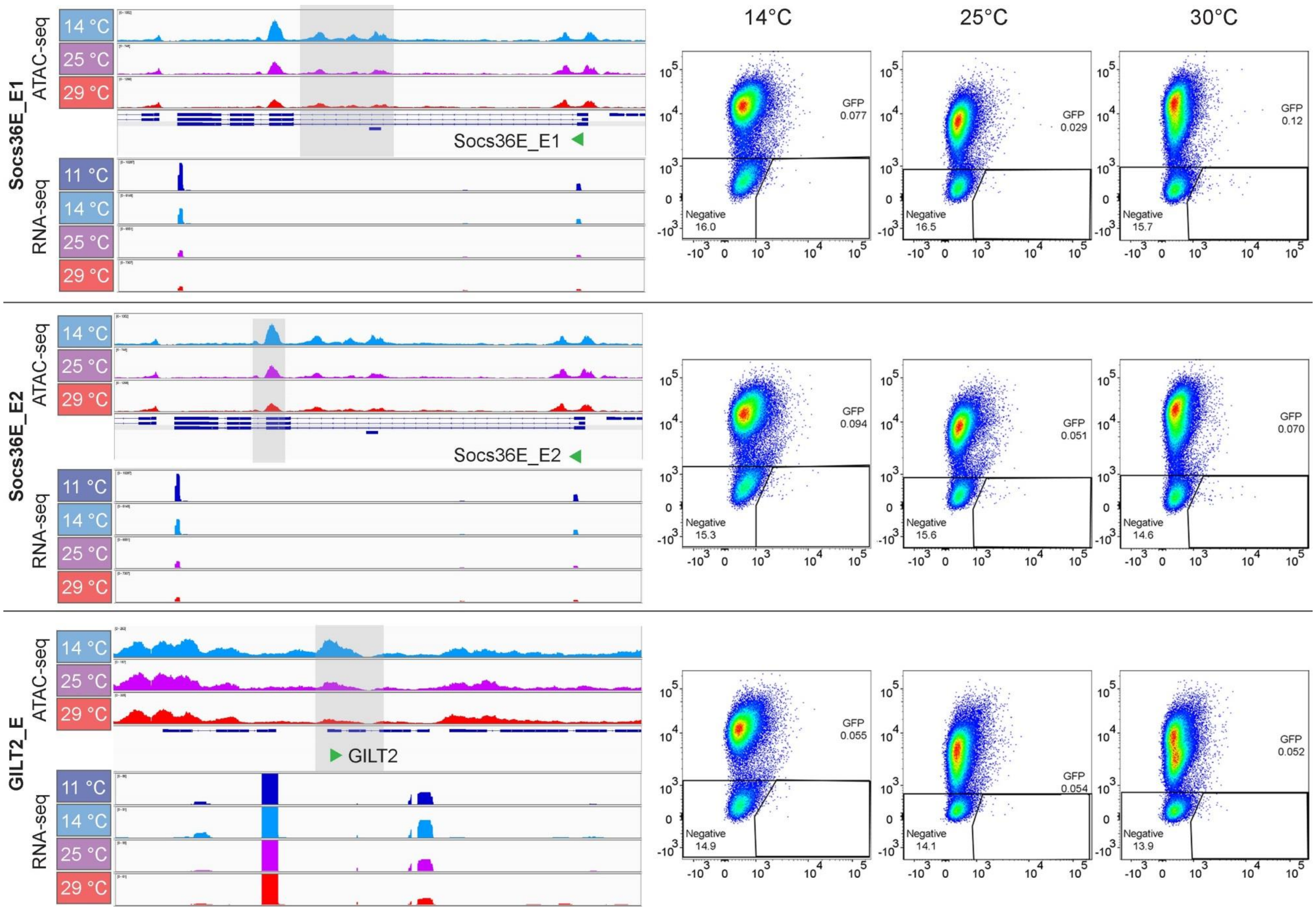

Bai et al., S9 Fig.

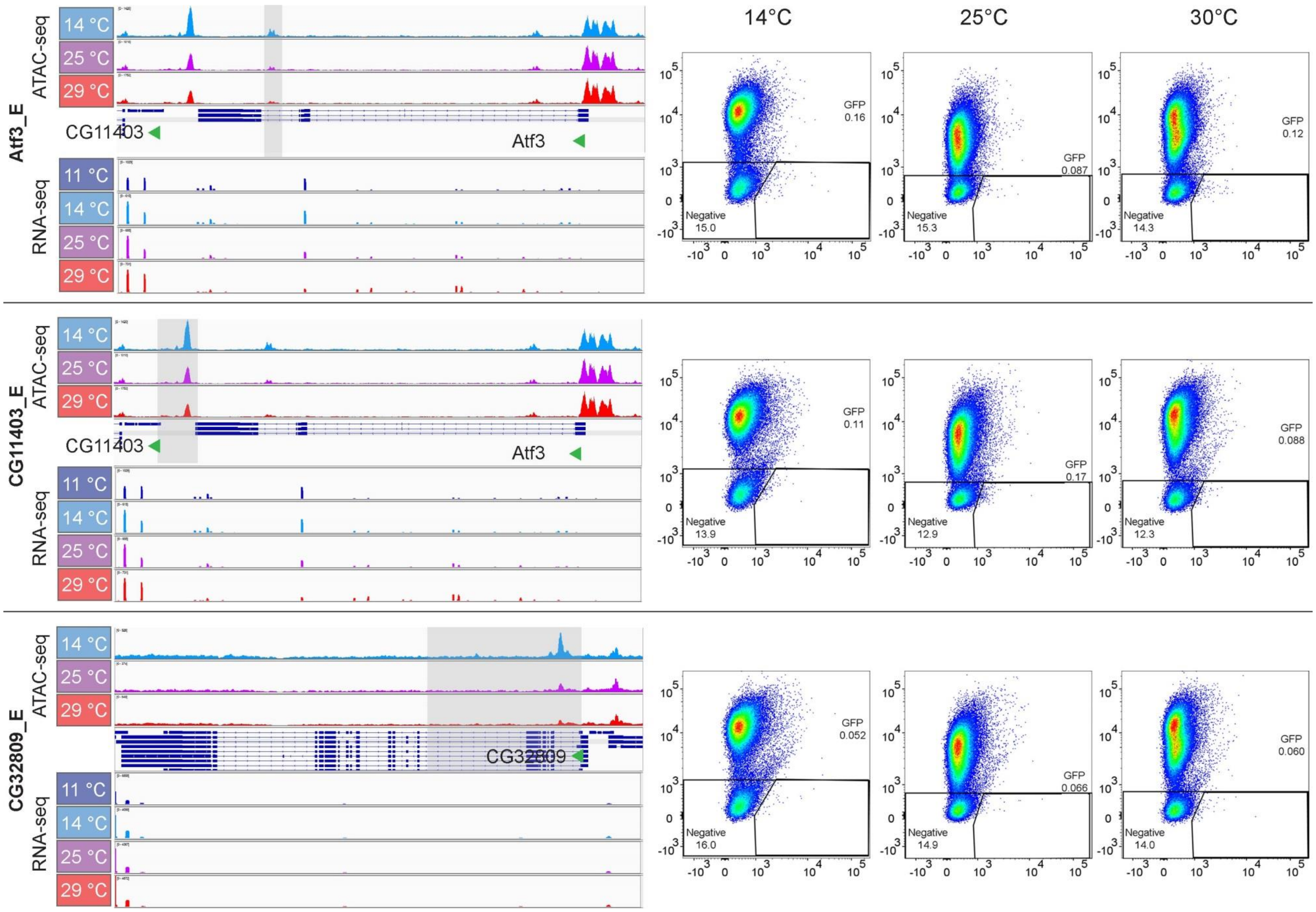

Bai et al., S9 Fig.

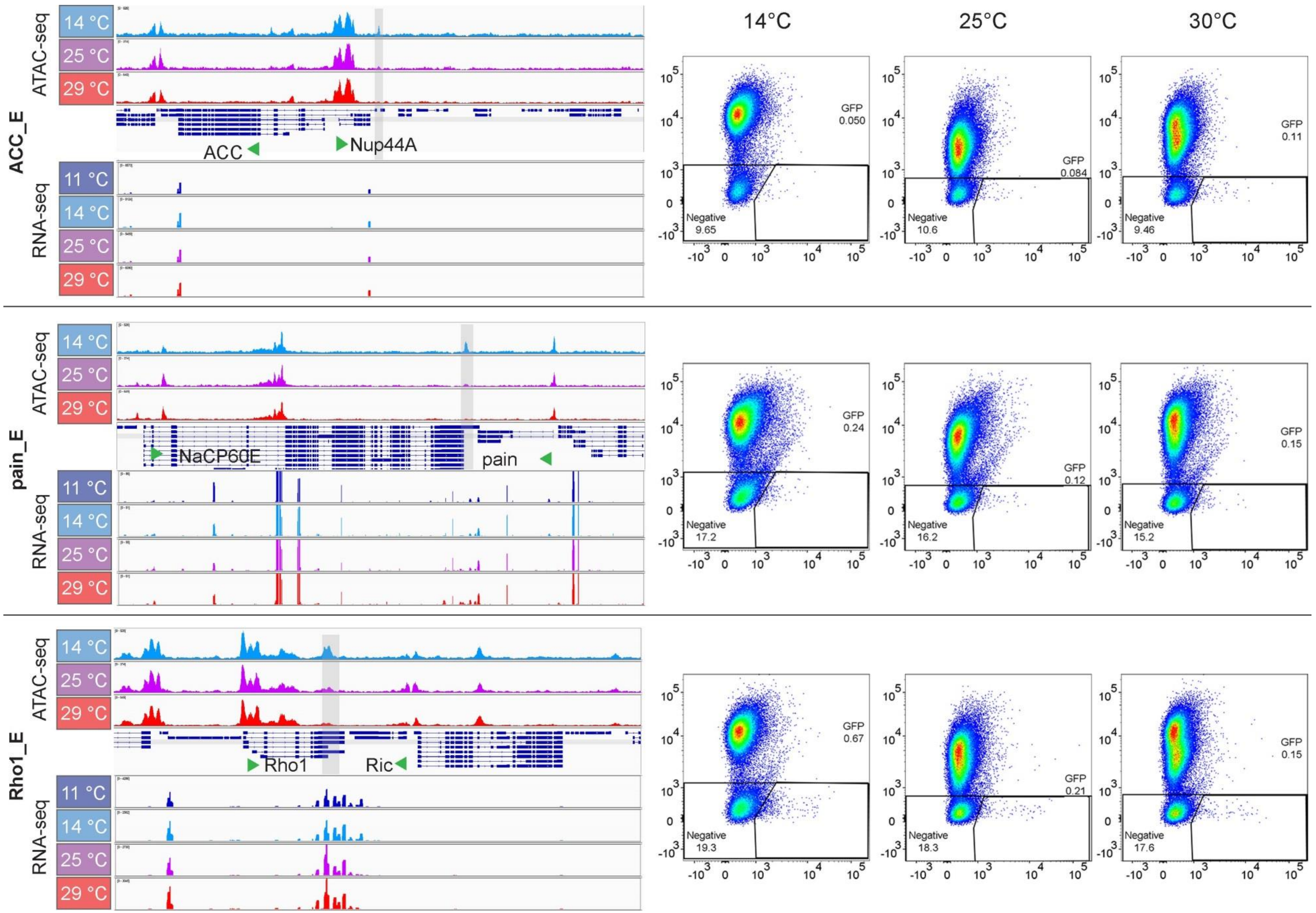

Bai et al., S9 Fig.

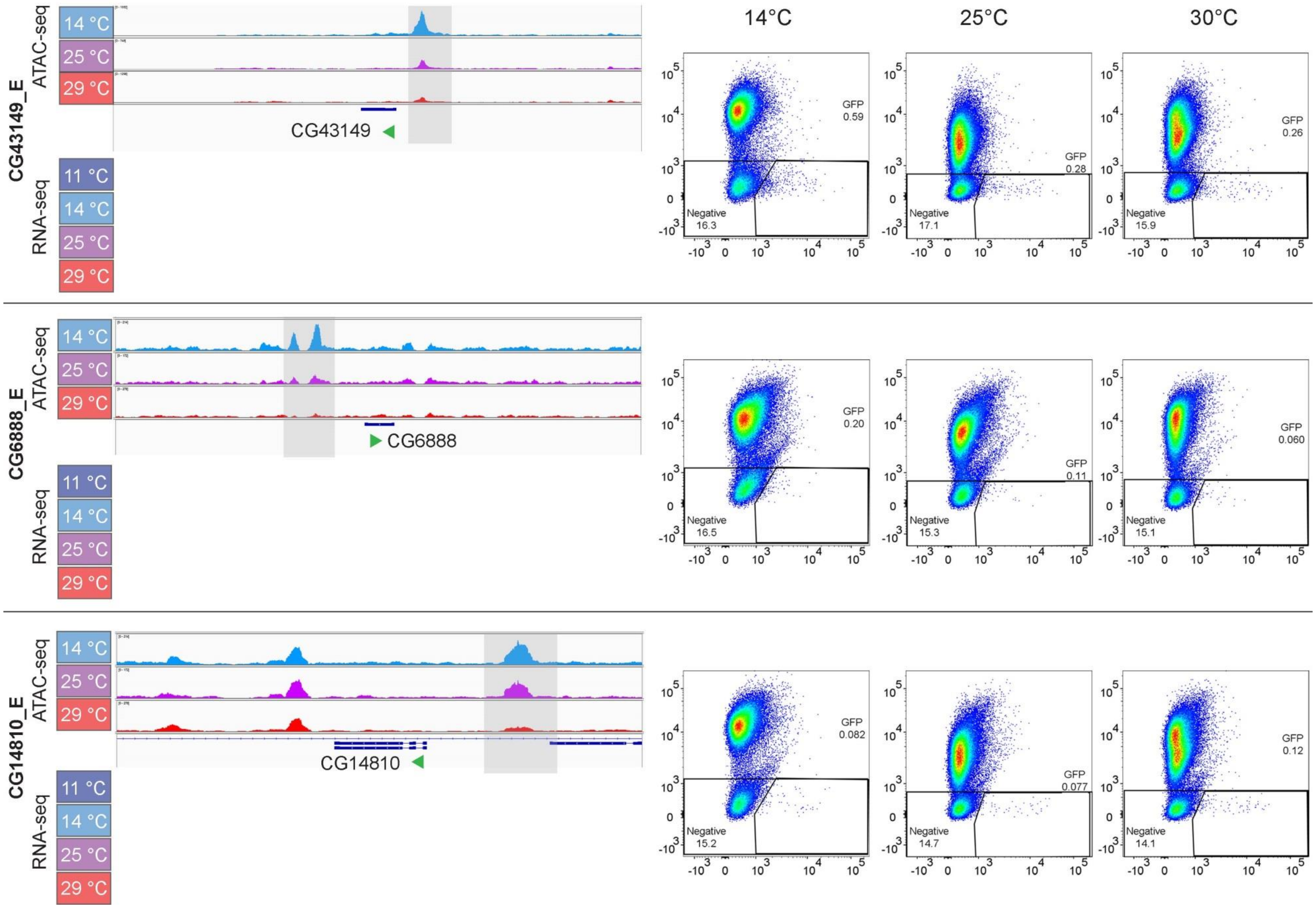

Bai et al., S9 Fig.

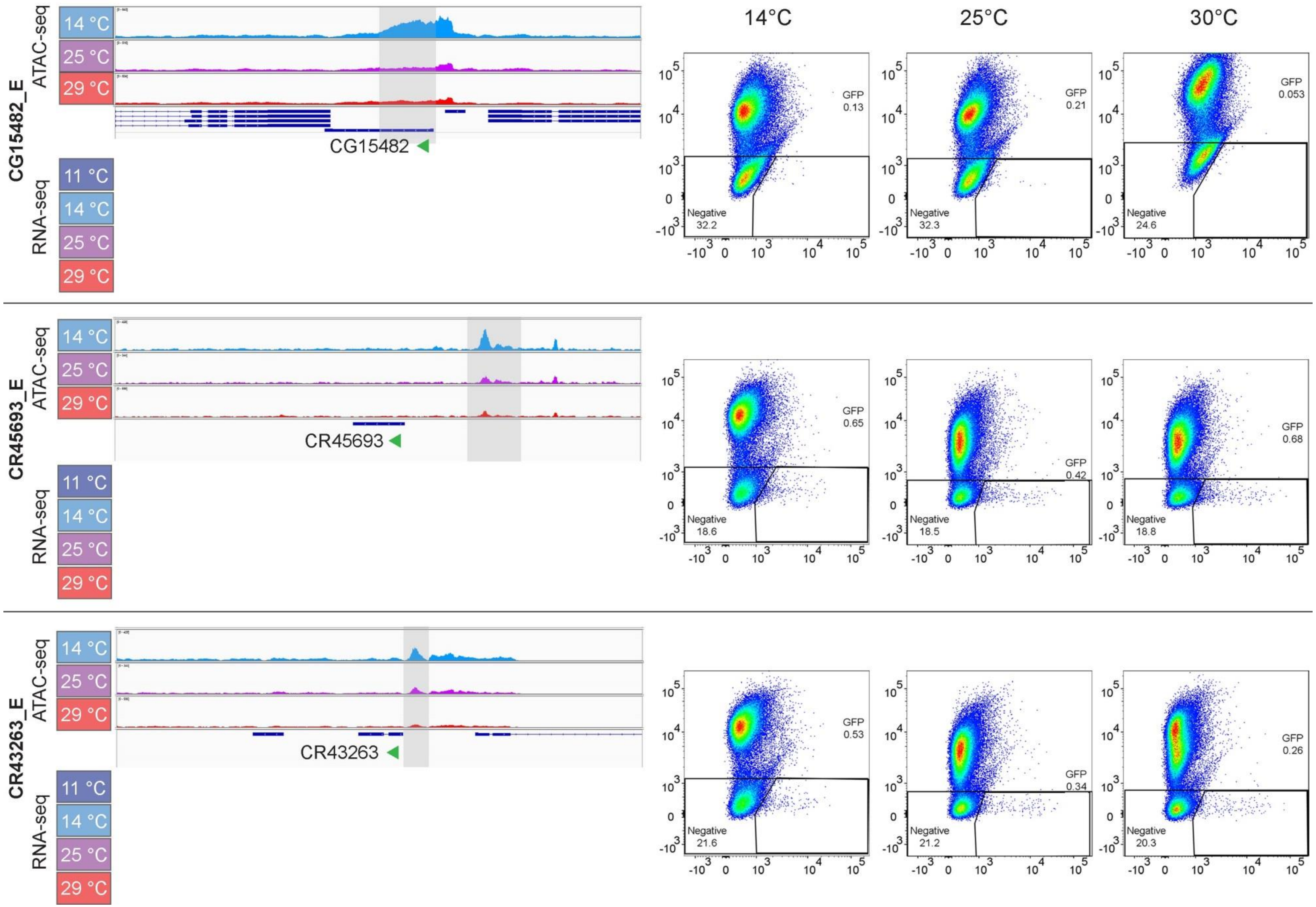

Bai et al., S9 Fig.

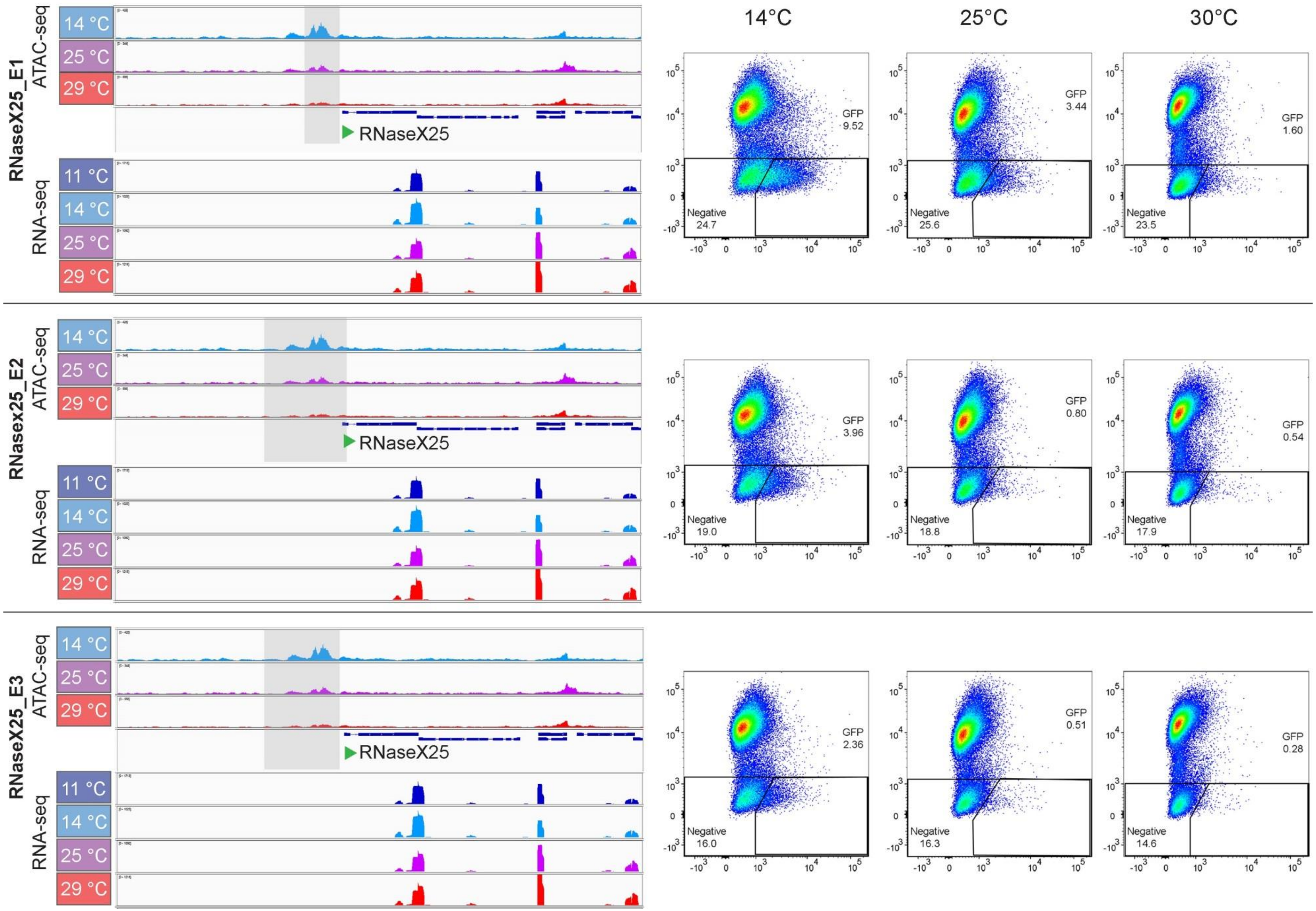

Bai et al., S9 Fig.

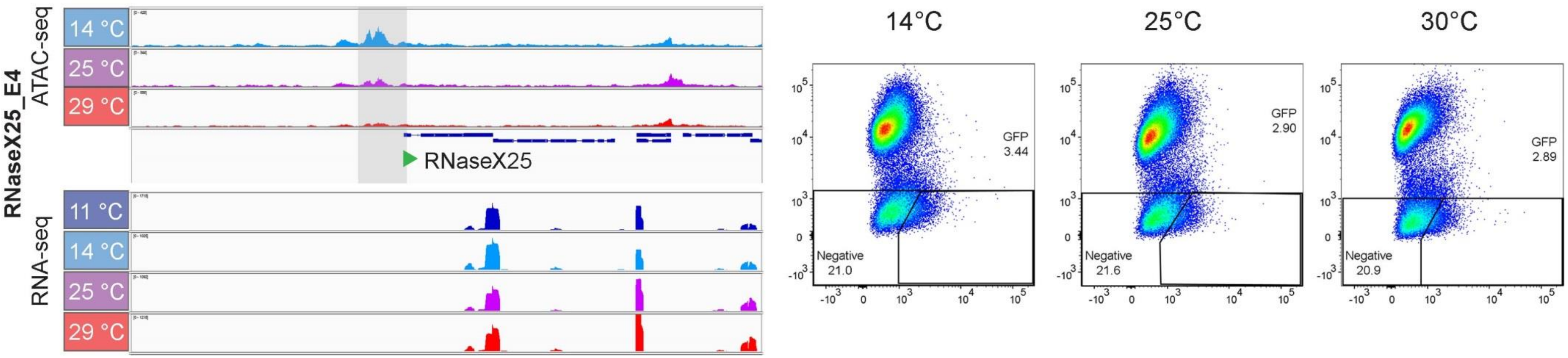

S9 Fig. Assay for temperature-dependent enhancer activity with candidate CREs.

(**A**) Table summarizing location and activity of candidate CREs analyzed after RMCE with SR9rg cells.

(**B**) Data for the analyzed candidate CREs. Candidate CREs (designation in bold on the left side) were selected based on data obtained by ATAC-seq (top three browser tracks) and 3' RNA-seq (bottom three browser tracks). Grey shading represents the region of the analyzed DNA fragment. Green arrowheads indicate the direction of transcription of the proximal gene. Scatter plots on the right side present green (x axis) and red (y axis) fluorescence intensities as determined by flow cytometry with SR9rg cells after RMCE and incubation at the indicated temperatures.
