# Supplementary material for: A cis-regulatory element promoting increased transcription at low temperature in cultured ectothermic Drosophila cells
Source: BMC Genomics. 2021 Oct 28;22:771. doi: 10.1186/s12864-021-08057-4 (PMC8555087; doi:10.1186/s12864-021-08057-4)
Supplement: Supplementary file 19 — Additional file 19: Fig. S10. Dissection of pst_E1 region. (A) Schematic illustration of analyzed truncation series, with transcription start sites (kinked arrows), untranslated regions (grey boxes), coding region (yellow boxes) and introns (white boxes) indicated. (B) Scatter plots of the results obtained by flow cytometry after RMCE with SR9rg cells and incubation at the indicated temperatures. [file 12864_2021_8057_MOESM19_ESM.pdf]

Bai et al., S10 Fig.

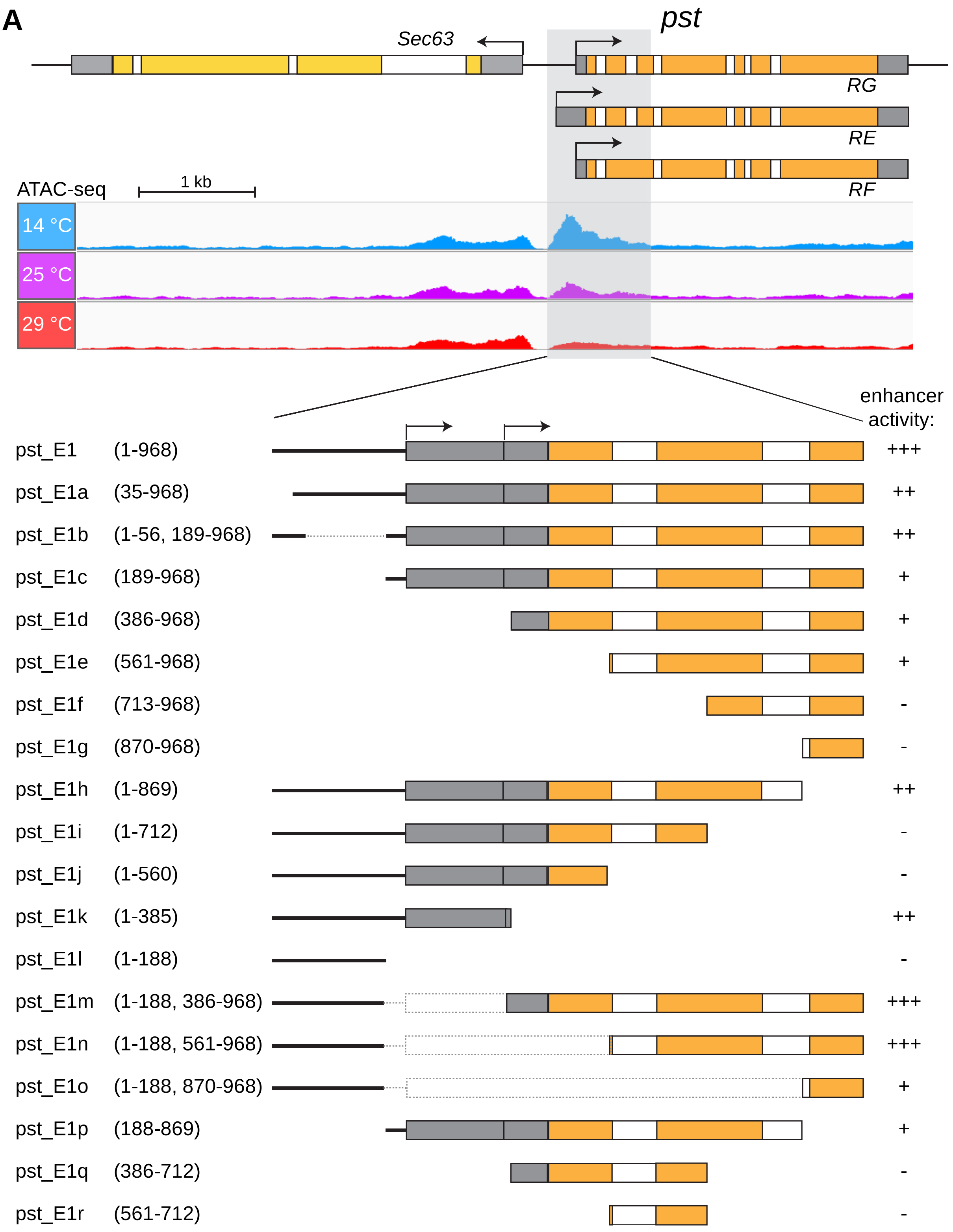

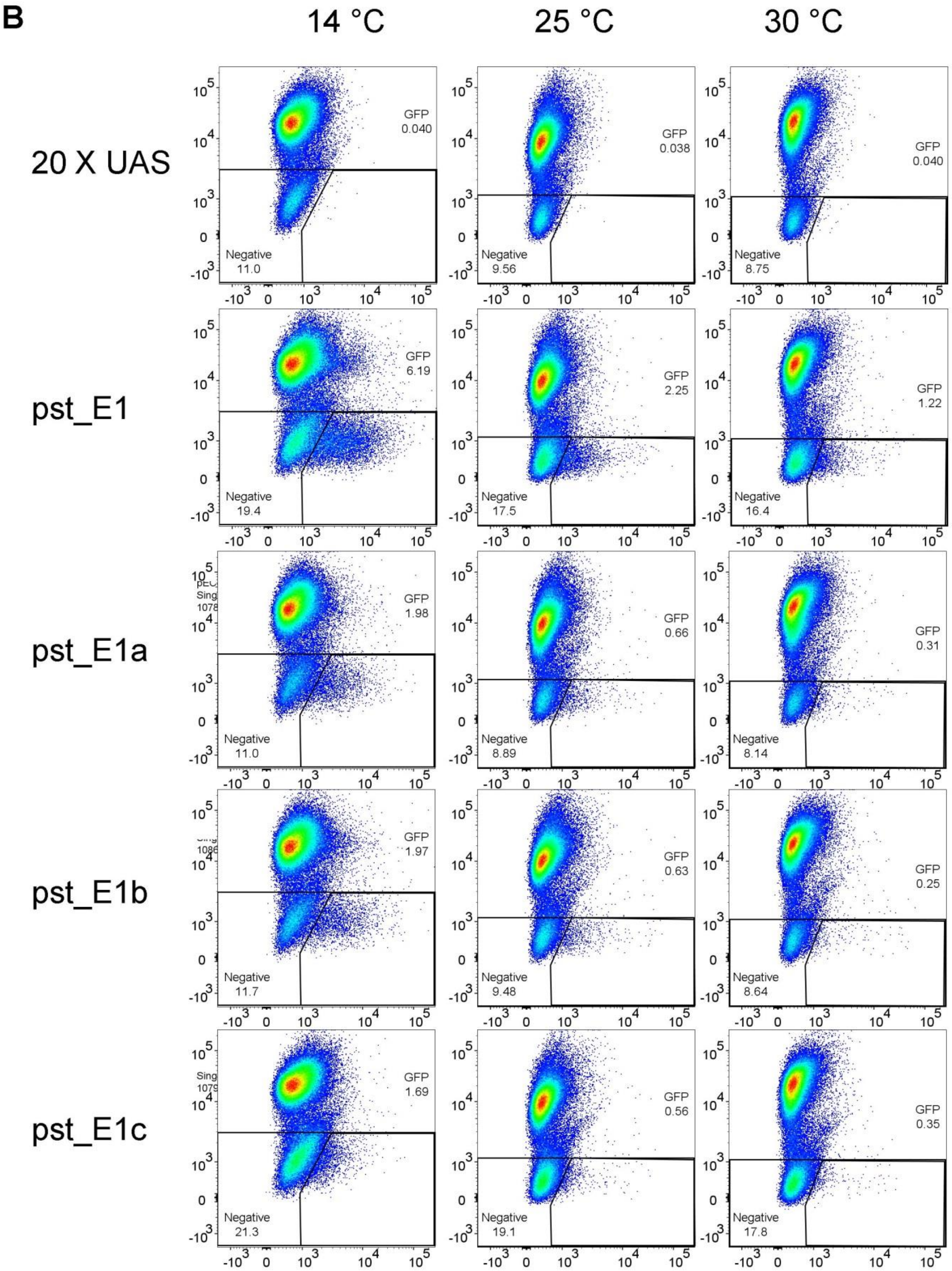
B
14 °C
25 °C
30 °C
20 X UAS
pst_E1
pst_E1a
pst_E1b
pst_E1c
GFP 0.040
Negative 11.0
GFP 0.038
Negative 9.56
GFP 0.040
Negative 8.75
GFP 6.19
Negative 19.4
GFP 2.25
Negative 17.5
GFP 1.22
Negative 16.4
GFP 1.98
Negative 11.0
GFP 0.66
Negative 8.89
GFP 0.31
Negative 8.14
GFP 1.97
Negative 11.7
GFP 0.63
Negative 9.48
GFP 0.25
Negative 8.64
GFP 1.69
Negative 21.3
GFP 0.56
Negative 19.1
GFP 0.35
Negative 17.8

Bai et al., S10 Fig.

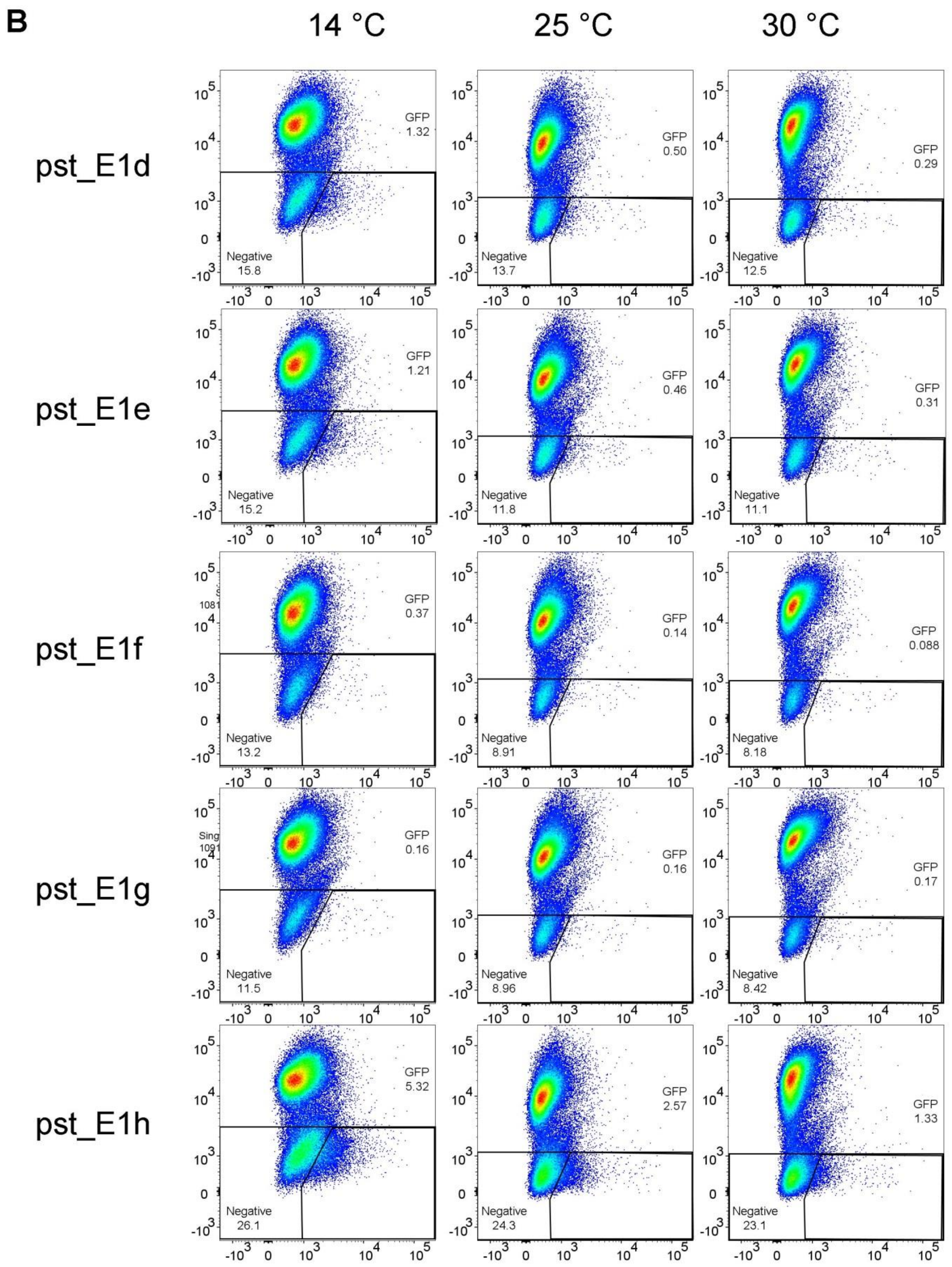

Bai et al., S10 Fig.

B

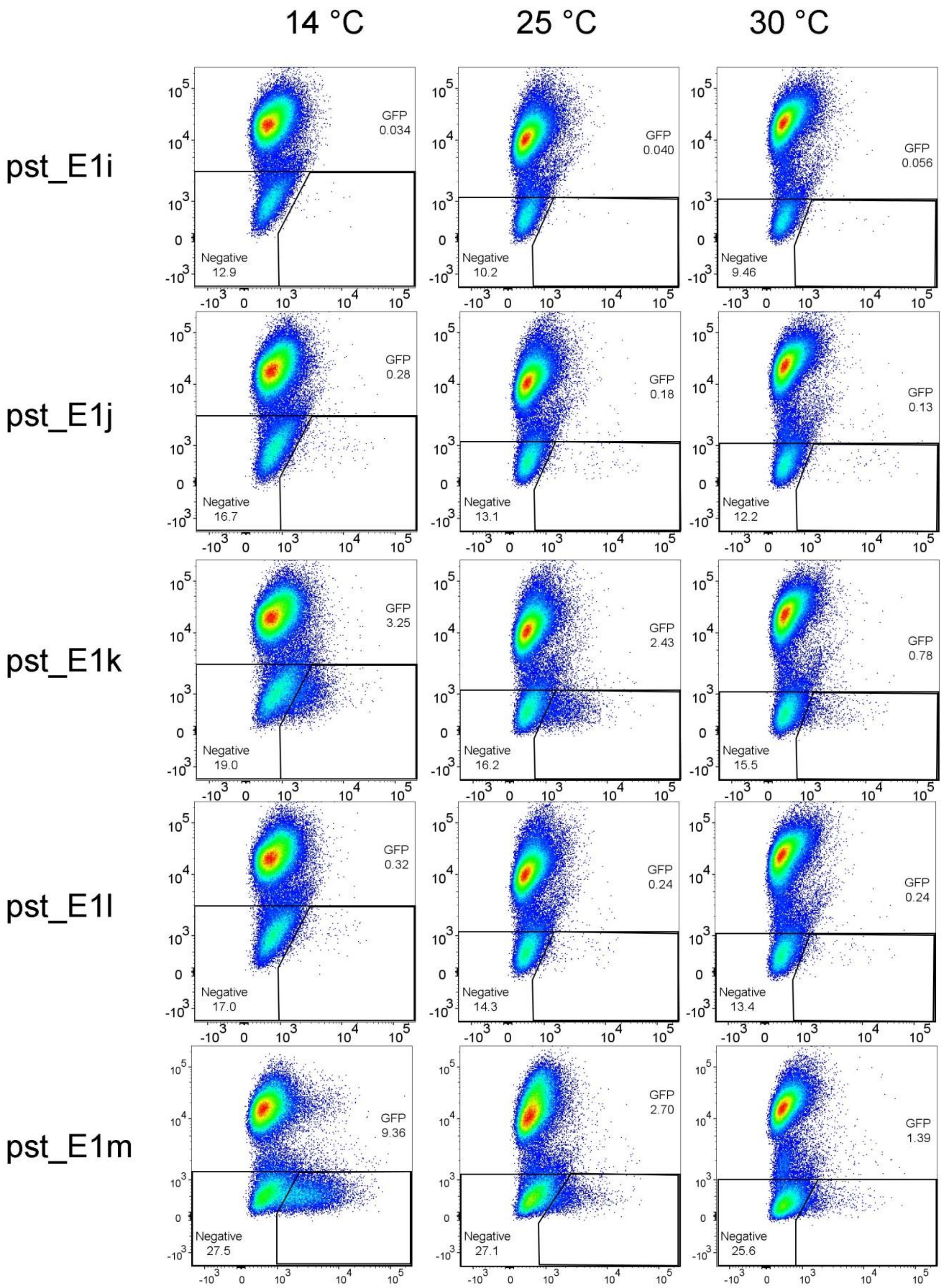

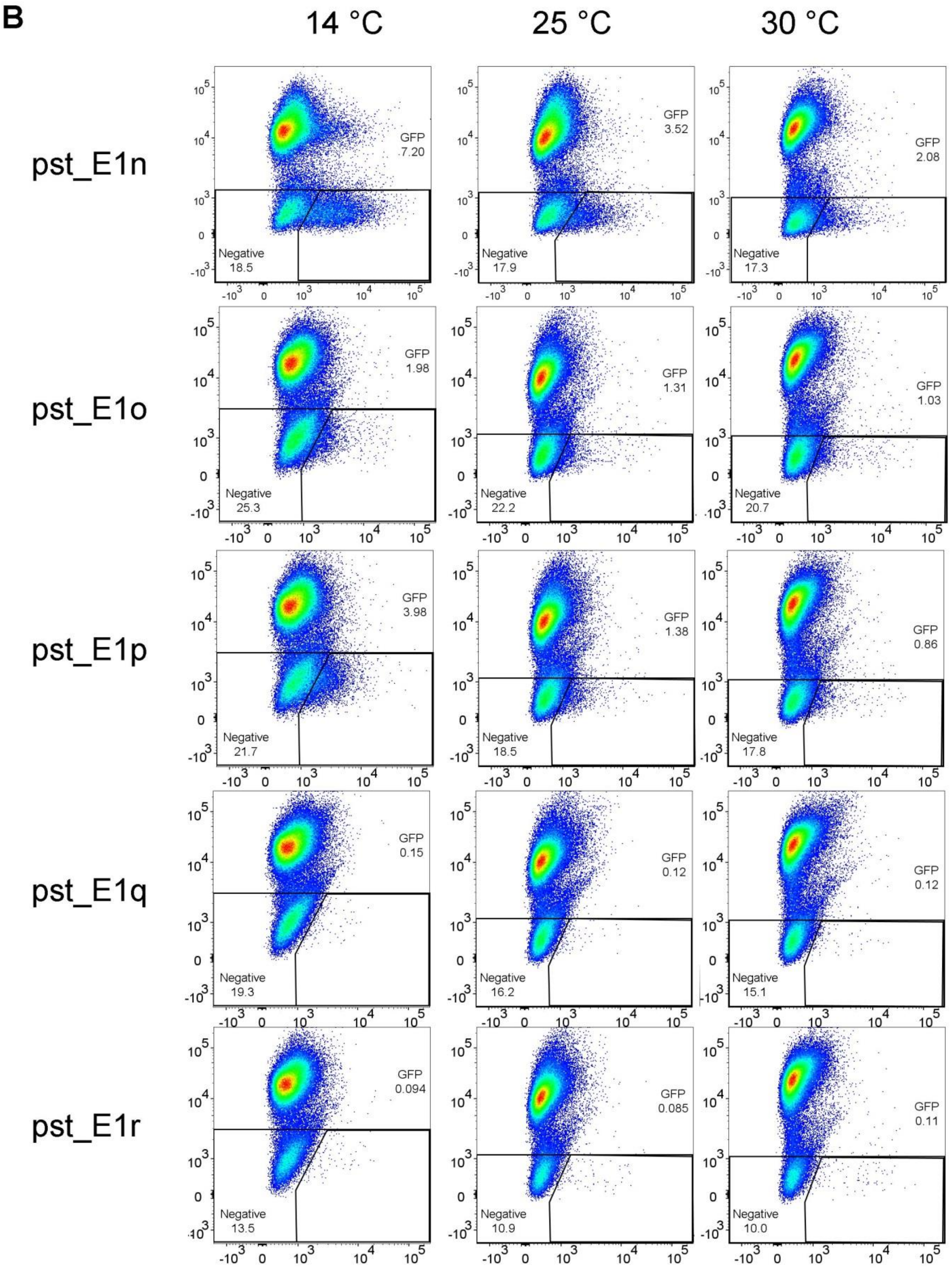

S10 Fig. Dissection of *pst*_E1 region

(**A**) Schematic illustration of analyzed truncation series, with transcription start sites (kinked arrows), untranslated regions (grey boxes), coding region (yellow boxes) and introns (white boxes) indicated.

(**B**) Scatter plots of the results obtained by flow cytometry after RMCE with SR9rg cells and incubation at the indicated temperatures.
